# Monoculture of Leafcutter Ant Gardens

**DOI:** 10.1371/journal.pone.0012668

**Published:** 2010-09-10

**Authors:** Ulrich G. Mueller, Jarrod J. Scott, Heather D. Ishak, Michael Cooper, Andre Rodrigues

**Affiliations:** 1 Section of Integrative Biology, University of Texas at Austin, Austin, Texas, United States of America; 2 Smithsonian Tropical Research Institute, Balboa, Ancon, Republic of Panamá; 3 Department of Bacteriology, University of Wisconsin, Madison, Wisconsin, United States of America; 4 Center for the Study of Social Insects, State University of São Paulo (UNESP), Rio Claro, São Paulo, Brazil; 5 Department of Biological Sciences, Santa Cruz State University (UESC), Ilheus, Bahia, Brazil; University of Arizona, United States of America

## Abstract

**Background:**

Leafcutter ants depend on the cultivation of symbiotic *Attamyces* fungi for food, which are thought to be grown by the ants in single-strain, clonal monoculture throughout the hundreds to thousands of gardens within a leafcutter nest. Monoculture eliminates cultivar-cultivar competition that would select for competitive fungal traits that are detrimental to the ants, whereas polyculture of several fungi could increase nutritional diversity and disease resistance of genetically variable gardens.

**Methodology/Principal Findings:**

Using three experimental approaches, we assessed cultivar diversity within nests of *Atta* leafcutter ants, which are most likely among all fungus-growing ants to cultivate distinct cultivar genotypes per nest because of the nests' enormous sizes (up to 5000 gardens) and extended lifespans (10–20 years). In *Atta texana* and in *A. cephalotes*, we resampled nests over a 5-year period to test for persistence of resident cultivar genotypes within each nest, and we tested for genetic differences between fungi from different nest sectors accessed through excavation. In *A. texana*, we also determined the number of *Attamyces* cells carried as a starter inoculum by a dispersing queens (minimally several thousand *Attamyces* cells), and we tested for genetic differences between *Attamyces* carried by sister queens dispersing from the same nest. Except for mutational variation arising during clonal *Attamyces* propagation, DNA fingerprinting revealed no evidence for fungal polyculture and no genotype turnover during the 5-year surveys.

**Conclusions/Significance:**

*Atta* leafcutter ants can achieve stable, fungal monoculture over many years. Mutational variation emerging within an *Attamyces* monoculture could provide genetic diversity for symbiont choice (gardening biases of the ants favoring specific mutational variants), an analog of artificial selection.

## Introduction

Cooperation and conflict within host-symbiont associations evolve under the constraints of stabilizing and destabilizing mechanisms [1], [2]. One of the key destabilizing mechanisms - competition between symbionts for shared resources supplied by a host – is particularly likely to drive evolution towards non-cooperative, antagonistic host-symbiont interactions. Symbiont-symbiont competition can select for symbiont features that enhance competitive ability at the expense of benefits that the symbionts provide for the host [3], [4]. Hosts are therefore expected to evolve mechanisms that minimize symbiont-symbiont competition [5], [6], [7], for example by associating with only a single symbiont type, by culling diversity of symbionts (including diversity that emerges through mutation within a population of associated symbionts), by allocating symbiont types to different niches within the host (effectively, partitioning the interaction network among symbiont types), or by forcing co-dependency of symbiont types on each other.

Leafcutter ants (genera *Atta* and *Acromyrmex*) are dependent on symbiotic fungi for food, which are thought to be grown by the ants in single-strain, clonal monoculture throughout the multiple gardens within a leafcutter nest. This traditional assumption of fungal monoculture derives primarily from natural-history observations collected 100 years ago [8], [9], [10]. First, gardens of new nests are started from a small pellet of fungal inoculum brought by the foundress queen from her natal nest. Second, nests of most leafcutter species are founded by single queens (monogyny; but see Discussion for polygynous leafcutter ant species), thus precluding mixing of fungi at the nest-founding stage. Third, fungal cultivars are propagated by the ants clonally within nests by planting mycelium taken from mature gardens onto garden substrate of newly prepared garden. These three natural-history observations lead to the long-standing assumption that each leafcutter nest cultivates a monoculture of fungus. Whereas monoculture of leafcutter fungi should be advantageous to fungus-growing ants because monoculture could help stabilize the mutualistic association (absence of cultivar-cultivar competition within the same nest), polyculture within leafcutter nests could be advantageous to the ants because different cultivars may provide the ants with different biochemical or enzymatic benefits [11] or provide genetic diversity that buffers leafcutter nests against diseases of the cultivars [12]–[17]. These advantages and disadvantages of monoculture versus polyculture apply also to other fungus-growing insects [18], [19].

Only a single study has so far tested for monoculture of leafcutter gardens with molecular methods [20]. Using Amplified Fragment Length Polymorphism (AFLP) genotyping, [20] failed to find any genetic diversity within small laboratory gardens of two *Acromyrmex* leafcutter species that had been bottlenecked through an even smaller transitional garden between field collection and establishment in the laboratory. Potential polyculture occurring naturally in the field (e.g., different cultivar genotypes grown in different gardens) may have been lost during the transfer into the laboratory. However, coexistence of two fungal genotypes in a chimaeric garden is thought to be unlikely in these two *Acromyrmex* species because the fungi appear to secrete incompatibility compounds that are distributed by the ants throughout gardens [20], preventing coexistence of two incompatible fungi in the same garden or invasion of incompatible cultivar types into an established garden. Although the cultivated fungi of *Acromyrmex* and *Atta* are closely related and can even be shared through lateral transfer of fungal clones between nests of these two ant genera [21], incompatibility factors between cultivars appear to be absent or are of lesser importance in the leafcutter ant *Atta texana*, which is able to co-cultivate several genotypically diverged fungi in experimentally created, chimaeric laboratory gardens [22].

Among fungus-growing ants, nests of *Atta* leafcutters are thought to be most likely to cultivate several distinct cultivar genotypes per nest because of the enormous sizes of the nests. Mature *Atta* nests have hundreds to thousands of gardens (estimated maximum of 5000 gardens [23]–[27]), whereas *Acromyrmex* leafcutter nests have either one large confluent garden or a small number of gardens in close spatial proximity (generally less than 10 gardens; 23, 28]. *Atta* gardens are discrete units, with an average garden size of about 30×20×20 cm^3^. Because the hundreds of *Atta* gardens can be spaced across an underground volume exceeding 1000 cubic meters, it seems more likely for *Atta* than for *Acromyrmex* that a single nest may cultivate different fungal genotypes in different sectors of its nest. Evolutionary theory of within-host symbiont diversity [3]–[7] and the aforementioned empirical work on laboratory colonies of *Acromyrmex* [20] led to the expectation that leafcutter ants generally cultivate a single cultivar clone per nest. We provide here the first empirical tests of within-nest cultivar diversity in the enormous *Atta* leafcutter nests.

We assessed within-nest cultivar diversity with highly polymorphic microsatellite DNA markers [29], using three experimental approaches. In *Atta cephalotes* and in *A. texana*, we tested for genetic differences between fungi from different nest sectors accessed through excavation. In *A. texana*, we also tested for genetic differences between fungi carried by dispersing queens emanating from the same *Atta* nest during a mating flight. Third, in *A. cephalotes* and *A. texana*, we resampled nests over a 5-year period and tested for persistence of the same clonal genotype within each nest. Lastly, to gauge the population bottleneck experienced by *Attamyces* fungi at the nest-founding stage, we estimated the number of mycelial cells carried by *A. texana* foundress queens in their infrabuccal pellets (a mycelial wad stored in the mouth and used by a foundress queen to start her own garden after dispersal). An estimate of the number of cultivar cells carried by a foundress is critical to assessing whether the ants passage their cultivar through an extreme bottleneck to help reduce evolutionary conflicts among genetically different cultivars, as predicted by theory [5], [6], [7]. DNA fingerprinting revealed no evidence for fungal polyculture within the surveyed *Atta* nests and no genotype turnover during the 5-year surveys, indicating that nests of these *Atta* species can achieve stable, fungal monoculture over many years.

### The leafcutter study systems *Atta* texana and *Atta* cephalotes

Like most fungus-growing ants, leafcutter ants grow fungi in sponge-like, three-dimensional gardens in cavities that are excavated by the ants in the soil, or that are constructed by the ants as thatched chambers at ground level or on trees [23], [28]. Leafcutter fungi are basidiomycetes in the agaric tribe Leucocoprini (fungal anamorph *Attamyces bromatificus*, teleomorph *Leucocoprinus gongylophorus*, Agaricales, Basidiomycota; [30]–[32]). Hundreds of *Attamyces* strains genotyped so far were all polyploid because of the multinucleate cells of *Attamyces* [29]. *Attamyces* fungi appear to be obligate symbionts, as they have not been found so far to grow independently of the ants [33], [34]. However, at least some *Attamyces* fungi are fruiting-competent and can produce spore-bearing mushrooms in laboratory colonies, or, so far known only from *Acromyrmex* leafcutter ants that thatch gardens at ground level, on mounds of field nests ([28], [31], [35], [36]; see Table 3 in [31] for a list of documented fruiting events of leafcutter fungi). The diverse leafcutter ant species are thought to associate with a single *Attamyces* species in a many-to-one co-evolutionary relationship [37]–[39]. Whereas the leafcutter ant clade is estimated to be about 8 million years old, the corresponding clade of *Attamyces* cultivars is less than 3 million years old [38], suggesting that novel *Attamyces* lineages arising within the clade have spread by means of horizontal transfer between ant lineages (so-called cultivar sweeps between leafcutter species [38]). Attine fungi are clonally propagated by the ants within and between nests, but incongruence of phylogenetic topologies between different genes indicates that recombination occurs occasionally over evolutionary time [37]. Coexisting cultivar genotypes may also recombine in experimentally created chimaeric gardens of lab colonies [22], most likely through the exchange of haploid nuclei between coexisting multinucleate (polyploid) cultivar mycelia.

*A. texana* and *A. cephalotes* are leafcutter ants with enormous colonies (more than 2 million workers) and extreme worker polymorphism, but the two species differ in many other respects. *A. cephalotes* has one of the largest distributions of any *Atta* species, ranging throughout lowland tropical rainforest from the Amazon Basin and the Atlantic Coast forests in Brazil (about latitude 15°S), to the Sierra de Los Tuxtlas in southern Mexico (latitude 20°N; [40]). *A. texana* is the northernmost species in the genus, ranging from Western Louisiana across eastern and central Texas to just south of the US-Mexico border [9], [23], [27]. Whereas about 5% of the newly founded *A. texana* nests are polygynous (generally with two queens; [41; U.G. Mueller unpublished]), nest-founding in *A. cephalotes* appears to be strictly monogynous [25, 42, 43; U.G. Mueller unpublished]. *A. cephalotes* constructs mounds with both perennial shallow and deep gardens (shallow gardens are about 0.5 meters deep; [24], [25]), favoring disturbed tropical forest with little seasonal, climatic changes. In contrast, *A. texana* has shallow gardens only in spring and relies throughout the year more on deep gardens (generally 1–4 meters deep; [9, 27, U.G. Mueller unpublished]) to evade summer droughts and winter temperatures in the seasonally variable subtropical habitat.

## Results

Although *Attamyces* genotypes differed between *Atta* nests in each population studied, we found no evidence of *Attamyces* polyculture within each of the surveyed *Atta* nests, with the exception of minor mutational variation that emerges during the continuous clonal propagation of *Attamyces* within nests of fungus-growing ants.

### 1. Genotyping of fungal pellets carried by dispersing queens from the same nest of *Atta texana*

In a large survey of dispersing females collected from three *A. texana* nests in 2006, all *Attamyces* pellets carried by females from each particular nest were genetically identical (44–50 pellets genotyped per nest), with the exception of two possible mutations (two different loci in two fungal pellets from different ant nests; Supporting Information Table S1). For the eleven microsatellite loci screened, this translates into an estimated cellular-division mutation rate of 1.12×10^−3^ per locus (Supporting Information Results S1). Apart from this mutational variation, we find no evidence that *A. texana* females emerging for a mating flight from a single nest carry inocula of different *Attamyces* genotypes. Females dispersing from a particular *A. texana* nest therefore appear to pick inocula from the same *Attamyces* clone as starter cultures for their new nests.

### 2. Resampling of pellet-cultivars carried by females from the same *A. texana* nests

In a longitudinal survey of mating flights of three *A. texana* nests between 2004–2010 (only one of these three nests was also studied under point 1. above), all *Attamyces* pellets carried by females from the same nest were genetically identical (at least three pellets from three females screened per year per nest; Supporting Information Table S1). Within each of the three *A. texana* nests screened, therefore, the *Attamyces* clones chosen by dispersing females for their pellets were genetically stable over a 6-year period.

### 3. Genotyping of fungal gardens excavated from *Atta texana* nests in Texas

We found no evidence of within-nest genetic diversity of *Attamyces* between excavated gardens of seven nests of *A. texana* (Supporting Information Table S2). Because few gardens were sampled per nest in *A. texana* (average of 3.7 gardens sampled/nest, range 2–8 gardens/nest), this result is less conclusive than the corresponding results for *A. cephalotes* (over 70 garden fragments sampled per nest; see next).

### 4. Genotyping of fungal gardens excavated from *Atta cephalotes* nests in Panama

We found no *Attamyces* diversity within each of the six *A. cephalotes* nests (an average of 73 *Attamyces* samples screened per nest, collected from at least three quadrants of each nest, 3–12 gardens for each quadrant, three fragments sampled per garden), except for five putative mutant *Attamyces* strains in five different gardens (Supporting Information Table S3). The cellular-division mutation rate at the microsatellite loci was estimated to range between minimally 4.56×10^−4^ per locus to maximally 1.14×10^−3^ per locus (Supporting Information Results S1).

### 5. Resampling of cultivars from the same *A. cephalotes* nests excavated in 2003 and 2008

The comparison of *Attamyces* genotype profiles did not reveal genotype changes within each of four nests sampled originally in 2003 and again in 2008 (two garden fragments genotyped per nest for each year; Supporting Information Table S4). Each *Attamyces* strain propagated by each of the four *A. cephalotes* nests therefore was clonally stable over a 5-year period.

### 6. Estimating the number of fungal cells in single pellets carried by females of *A. texana*

The average number of colony forming units (CFUs) per pellet was 543.5 (StDev = 355.2, n = 28, range 60–1500; Supporting Information Table S5). The average number of CFUs was higher for pellets from Nest 1 at Brackenridge Field Lab (average = 600.8, StDev = 371.1, n = 20, range 60–1500) than for pellets from Nest A at Hornsby Bend (average = 400.1, StDev = 280.9, n = 8, range 188–940), but this difference was not significant (two-tailed t-test for unequal sample sizes, p = 0.14). Because most CFUs probably derive from aggregates of many *Attamyces* cells, the pellet which a female *A. texana* uses as a starter inoculum for her first garden probably contains a population of minimally several thousands of cultivar cells.

## Discussion

DNA fingerprinting reveals no evidence for the coexistence of diverged cultivar genotypes in single nests of *Atta cephalotes* or of *A. texana*, except for mutational variants that are expected to arise under long-term clonal propagation of *Attamyces* within gardens. Mutational variation appears to arise at estimated mutation rates (10^−3^–10^−4^) that are expected for the kind of di- and tri-nucleotide microsatellite loci used for DNA fingerprinting of *Attamyces* [44]–[47]. Because we fail to find evidence for polyculture of significantly diverged cultivar strains, our study confirms the hypothesized fungal monoculture for the hundreds to thousands of gardens within a single nest of both *A. cephalotes* and *A. texana*.

The finding of monoculture in the two *Atta* species is consistent with the reported monoculture in small laboratory gardens of two *Acromyrmex* species [20]. As in our study, [20] failed to find any genetic variation within single gardens (not even artifactual variation was found in the AFLP fingerprinting screens of [20]). However, the *Acromyrmex* gardens screened in [20] were from laboratory colonies that had been passaged through a small garden stage between collecting and establishment in the laboratory, leaving open the possibility that field nests of *Acromyrmex* may culture different fungi in different gardens of a nest. Our study on *Atta* tested for differences between different gardens in field nests, and establishes monoculture by sampling across the hundreds to thousands of gardens of single *Atta* nests.

Monoculture in leafcutter nests is likely maintained by several mechanisms, including (a) the transgenerational passage of the cultivar through a small bottleneck (our study on *A. texana* pellets estimates a population of several thousand *Attamyces* cells in the starter culture at nest founding); (b) possible weeding of secondary *Attamyces* strains if they were to enter an established garden (*Attamyces* weeding in the form of symbiont choice; [22]); and (c) cultivar-cultivar competition by differential growth or by secretion of incompatibility factors that preclude co-existence of incompatible *Attamyces* strains within a single, chimaeric garden (such incompatibility factors appears to occur in *Acromyrmex* leafcutter ants [20]). The observed minor mutational variation in *Atta* gardens is significant, as any such variation at non-neutral loci provides the raw material for cultivar evolution, either through direct selection on the cultivar in cultivar-cultivar competition [20], [31], ant-mediated selection on the cultivar through symbiont choice (‘artificial selection’) [22], [31], [48], [49], or selection on ant-fungus combinations [31], [50].

Monoculture and long-term persistence of the same fungal clone in field nests of *Atta* leafcutter ants has fundamental implications for the evolution and ecology of the leafcutter ant-fungus mutualism:

(a) Because of the longevity of *Atta* nests (10–20 years), because of the clonal transfer of cultivars between ant generations, and because of the persistence of the same cultivar genotype across many years within a nest documented here for the first time, partner fidelity feedback [2], [51] inherent in long-term ant-fungus co-dependency is a likely mechanism stabilizing the ant-fungus mutualisms. Partner fidelity feedback alone should impede the invasion of non-productive cultivar types into populations of *A. texana*, but a second mechanism, ant-mediated symbiont choice that biases cultivar propagation against inferior cultivar mutants, may also operate in the *Atta* ant-fungus mutualism [2], [51]. However, lab experiments quantifying symbiont choice in *A. texana* suggest that choice may be a comparatively weak mechanism, as workers do not show a strong and consistent cultivation-bias between closely-related *Attamyces* strains presented to the ants in laboratory experiments [22, R. Sen & U.G. Mueller unpublished].

(b) Monoculture of fungi in the long-lived, sessile *Atta* nests is expected to facilitate build-up of specialized diseases, which is the bane of clonally propagated crops in human agriculture (e.g., banana, sugar cane, potato; [52]–[54]). Several integrated defenses against diseases permit such long-term monoculture in *Atta* nests. First, a cast of small *Atta* workers is dedicated largely to the tending and cleaning of garden [55], and these workers monitor gardens intensively, controlling pathogens early during disease outbreaks before diseases can build up to unmanageable levels. Second, *Atta* ants sequester their gardens in underground chambers that shelter gardens against influx of pathogens and that reduce cross-infection between gardens. Sequestration into discrete garden units also permits the ants to respond locally to disease, for example by sealing off an infected garden and thus prevent a pandemic spread of a disease throughout a nest. On several occasions did we encounter such sealed gardens during our excavations of *A. texana*. As a last resort, *Atta* may even move an entire nest to a new location, moving all healthy gardens and leaving diseased gardens behind [56]. Third, the cultivated fungus and the ant farmers secrete antibiotics that help suppress diseases in ant nests [14], [57]–[60]. Fourth, unlike many other fungus-growing ants that can be covered by integumental accretions that contain antibiotic secreting actinomycete bacteria [61], [62], *Atta* species do not have such integumental accretions [58], [59], [63], [64]; however, like other fungus-growing ants, *Atta* gardens contain a great diversity of antibiotic-secreting microbes in the biofilms and matrix of the gardens (the so-called garden microbiome) that may help suppress diseases, as first suggested by [65] and further elaborated by [15], [17], [63]. Because the garden microbiome contains microbes with known antibiotic properties [63], [65], *Atta* ants appear to manage, in addition to the primary cultivars, an array of “auxiliary” microbes providing disease suppression and other services [15], [63], [66].

(c) Because of monoculture in *Atta* colonies, it would seem sufficient to sample only a single chamber in population-genetic studies of *Attamyces*, or sample from infrabuccal pellets carried by extranidal workers. However, detailed studies of within-nest cultivar diversity remain necessary for the massive nests of *Atta laevigata* and *A. capiguara* (which have 5–10 times the number of gardens as the two *Atta* species surveyed here [26]) and for deep gardens of any *Atta* species, which were not sampled in our study. Most importantly, future studies should survey leafcutter species with multiple foundresses (polygyny), such as the desert leafcutter *Acromyrmex versicolor* [67], where several females carrying distinct *Attamyces* genotypes may co-found a common garden. Preliminary investigation of gardens from polygynously-founded *Acro. versicolor* colonies did not indicate co-cultivation of several *Attamyces* strains or recombination between *Attamyces* strains in such polygynous colonies (R. Clark & U.G. Mueller unpublished).

(d) A foundress queen of *A. texana* carries a population of minimally several thousand cultivar cells per infrabuccal pellet as a starter culture for her incipient garden. This could suggest that *Atta* ants do not passage their cultivar through an extreme bottleneck to help reduce evolutionary conflicts among co-cultivated and genetically distinct cultivars, as is typical for many symbioses between macro- and microorganisms [5], [7]. However, it remains possible that the pellet population of several thousand *Attamyces* cells derives from a drastically bottlenecked founder population of a few cultivar cells gathered by a female for her pellet. Females incorporate multiple fragments of substrate into their pellet (some of the substrate appears suffused with mycelium, other substrate appears to be relatively fresh leaf fragments containing chlorophyll), suggesting that foundress queens sample from both mature and from young garden for their pellets, rather than from a single source. Future studies should elucidate how foundress queens amass the mycelium in their pellet and specifically determine (i) whether females gather mycelium from a single garden fragment or from multiple fragments; (ii) whether females gather only a few cells initially then permit growth to thousands of cells within a pellet; and (iii) whether females stress the mycelium to depress population size (and thus potentially eliminate variation) and at the same time test for fitness and viability of the chosen mycelium. Evolutionary theory of cooperation predicts that attine females should exhibit careful partner choice when picking mycelium for the pellet [31], perhaps even screening for honest indicators of cultivar fitness in the chosen garden fragment or during growth in the infrabuccal pocket. Concepts and experimental approaches developed within the context of mate choice and sexual selection may be able to help develop tests of cultivar choice by attine females when they gather mycelium for their pellets [31]. Such experiments on symbiont choice should be possible, for example, by expelling the pellet from a virgin female in a laboratory colony, then monitoring how the female chooses mycelium for a replacement pellet.

### Conclusions

A century after natural-history observations on *Atta* nest-founding first suggested the hypothesis that *Atta* cultivate monocultures in their enormous nests [8], [9], [10], we show here that *Atta* leafcutter ants can indeed achieve stable, fungal monoculture over many years and that mutational variation can arise within a nest's *Attamyces* monoculture. Additional variation may be introduced if novel *Attamyces* strains enter the nest and recombine with the resident strain; recombination appears to occur in rare cases in experimental laboratory colonies of *A. texana* [22, R. Sen, H.D. Ishak, and U.G. Mueller unpublished], but recombination has so far not been observed directly in natural *Atta* nests in the field. Any mutational and recombinational variation within a single *Atta* nest could provide the raw material for *Attamyces* evolution driven by symbiont choice (cultivation biases of the ants favoring or disfavoring *Attamyces* variants coexisting in the same nest), an analog of artificial selection operating in human agriculture.

## Materials and Methods

### 1. Genotyping of fungal pellets carried by dispersing queens from the same nest of *Atta texana*

Unmated females carrying infrabuccal fungal pellets were collected in May just prior to predawn mating flights from the mounds of three *A. texana* nests at Brackenridge Field Laboratory (BFL), University of Texas at Austin (colony UGM050509-01 = BFL1, N30.284444° W97.781944°; colony UGM050509-02 = BFL2, N30.280833° W97.778889°; colony UGM050509-07 = BFL7, N30.282153° W97.779391°) and two *A. texana* nests at Hornsby Bend Environmental Research Center (colony UGM060121-01 = A-colony, N30.232837° W97.651624°; UGM060121-02 = B-colony, N30.232333° W97.653005°). The five *Atta* colonies were chosen because of easy access to the mounds during the mating flights. The five nests were mature with large mounds (>7 m mound diameter), and therefore at least 5–10 years old. Based on field records collected at BFL, nest UGM050509-01 was probably founded between 1994–1996, but the founding dates of the other nests are unknown. Fungal pellets were expelled sterile from the females as described in [63]. Pellets were stored individually at −80°C in 100% ethanol for microsatellite DNA fingerprinting. The main survey of pellet-cultivar diversity in *A. texana* was conducted in May 2006, when three nests were sampled intensively, nest UGM050509-01 (#1 Nest BFL; pellets from 44 females), nest UGM060121-01 (A-Nest Hornsby; pellets from 48 females), and nest UGM060121-02 (B-Nest Hornsby; pellets from 50 females) (Supporting Information Table S1).

### 2. Resampling of pellet-cultivars carried by females from the same *A. texana* nests

The main survey of pellet-cultivar diversity on three nests was conducted in 2006 (nests UGM050509-01, UGM060121-01, and UGM060121-02; see above). For nest UGM050509-01, additional samples were available for the years 2004, 2005, 2007–2010 (one pellet from each of three females per year), yielding for this nest a continuous seven-year record (total of 59 pellets). For nest UGM050509-02 (not part of the 2006 survey), three pellets were genotyped for each of 2004, 2007–2010 (discontinuous six-year record, total of 15 pellets). For nest UGM050509-07 (also not part of the 2006 survey), two, three, and one pellet were available for 2004, 2009, and 2010 (discontinuous six-year record, total of 6 pellets). Table S1 (Supporting Information) summarizes all the sample sizes for the *A. texana* pellets genotyped for the years 2004–2010. As above, fungal pellets were expelled sterile from the females as described in [63]. Pellets were stored individually at −80°C in 100% ethanol for microsatellite DNA fingerprinting.

### 3. Genotyping of fungal gardens excavated from *Atta texana* nests in Texas

Fungal diversity within single nests of *A. texana* was assessed primarily by genotyping the fungal pellets carried by females emerging from nests for a mating flight (see above), but a few nests were also repeat-sampled by excavation as part of a larger population-genetic survey of *A. texana* cultivars [U.G. Mueller unpublished] and as part of a phenological survey of the non-cultivar fungi growing in gardens of *A. texana* [17]. For seven nests, fungal samples from at least two gardens were genotyped (average of 3.7 gardens genotyped per nest, range 2–8 gardens) (Supporting Information Table S2).

### 4. Genotyping of fungal gardens excavated from *Atta cephalotes* nests in Panama

Six nests of the tropical leafcutter ant *Atta cephalotes* were excavated in December 2003 along Pipeline Road, Parque Soberanía, Republic of Panamá. Nest mounds had a diameter of at least 14 meters (Table S3), and nests were therefore at least 5–10 years old. Nests mounds were excavated in the area with the greatest and freshest digging activity of the ants (greatest accumulation of fresh soil excavate dumped outside the nest by the ants), as fungus gardens could be found predictably at a depth of 20–100 cm in this area. Once a garden was located, other gardens were invariably found nearby (within 50–100 cm lateral digging). Unlike other *Atta* species adapted to drier habitats, *A. cephalotes* is a forest-specialized species; most nests are shaded during the day, the top soil on the mound remains relatively moist, and many gardens in an *A. cephalotes* colony occur therefore at shallow depths [23]–[25], [42]. We therefore concentrated our sampling on the topmost gardens, which we generally encountered at depths of 30–60 cm (depths of sampled gardens are listed in column D of Table S3). Because we did not collect gardens from deeper layers, our study cannot rule out fungal genotype differences between surface gardens and deep gardens; however, because most gardens in *A. cephalotes* nests occur at shallow depths [23]–[25], [42], we assume that our sampling regime covered a significant portion of gardens in a given nest. To maximize spatial coverage of gardens in different locations in a given nest, we first divided the nest mound in four quadrants, then attempted to locate gardens in each quadrant, but maximizing the distance between excavated holes (i.e., by placing the hole towards the periphery of the area of fresh ant digging activity). We were able to find gardens in all four quadrants in two nests (Nests 2 and 9; Table S3), but only in three quadrants in the remaining four nests (Nests 6, 8, 12, and 13); for these latter nests with only three successful quadrants, a greater number of gardens were sampled per quadrant. For each nest, we aimed at sampling 25 gardens, located, if possible, in equal proportions in each quadrant. For each garden, we preserved three garden fragments in three separate vials with DMSO-salt buffer [68], collecting from the most distant areas in the garden. This sampling regime (different quadrants, several neighboring gardens per quadrant, three fungal samples per garden from different garden parts) aimed at maximizing the chance of finding genotype differences between cultivars within the large *Atta* nests. Nest information and sample sizes are summarized as follows:

**Nest 2:** N09.1521° W79.7361°, 22 m×14 m mound area, 75 fungal samples from 25 gardens.

**Nest 6:** N09.1381° W79.7361°, 25 m×11.5 m mound area, 76 fungal samples from 25 gardens.

**Nest 8:** N09.1478° W79.7321°, 14 m×14 m mound area, 68 fungal samples from 23 gardens.

**Nest 9:** N09.1597° W79.7399°, 20 m×18 m mound area, 73 fungal samples from 26 gardens.

**Nest 12:** N09.1577° W79.7475°, 15 m×11 m mound area, 72 fungal samples from 24 gardens.

**Nest 13:** N09.1584° W79.7471°, 20 m×40 m mound area, 74 fungal samples from 25 gardens.

The average number of samples per nest was 73.0 garden fragments (range 68–76), for a total of 438 garden fragments between all six nests. Each sample was genotyped at ten microsatellite loci (see *Microsatellite Marker Genotyping* below). In sum, to evaluate genetic differences between fungal samples within *A. cephalotes* nests, we screened an average number of 730 loci per nest in an average of 73 garden fragments per nest (10 loci screened per fragment).

### 5. Resampling of cultivars from the same *A. cephalotes* nests excavated in 2003 and 2008

In June 2008, nearly five years after the first sampling of the six *A. cephalotes* nests in December 2003, it was possible to relocate four of the original six nests at the original collection sites and obtain garden samples for each nest through excavation (Nests 2, 9, 12, and 13; the two remaining nests were inactive at the original mound because they had either migrated or died since 2003). Two fragments of a single garden were collected for each of these four nests and preserved in 100% ethanol. These 2008 samples, and corresponding samples for each nest collected in 2003, were genotyped at 15 loci using the multiplexed microsatellite screen (Supporting Information Table S4).

### 6. Estimating the number of fungal cells in single pellets carried by females of *A. texana*

The study was conducted in May 2006 when a large number of *A. texana* females were collected at mating flights to screen infrabuccal pellets for the presence of microbes other than the cultivar [17], [63]. Methods for the maceration of pellets in buffer and plating are detailed in [63]. In brief, pellets were sterilely expelled from winged female *A. texana* within a few hours after they were collected from mounds on the morning of a mating flight. Pellets were macerated in 1 ml buffer and vortexed, then the entire suspension was plated on Potato Dextrose Agar (PDA; 9.5 cm diameter Petri dish) supplemented with the antibacterial chloramphenicol. Plates were sealed with parafilm and incubated at room temperature (about 20–23°C). The number of colony-forming units (CFUs; single cells or aggregates of multiple cells, each giving rise to a colony growing on the cultivation plate) of the *Attamyces* cultivar growing on each PDA plate was counted two weeks after plating. Twenty pellets were screened from females from Nest 1 (UGM050509-01) at Brackenridge Field Lab, and 8 pellets from females from Nest A (UGM060121-01) at Hornsby Bend Environmental Research Center (Supporting Information Table S5). Because each CFU is comprised of one to many cultivar cells, the count gives a minimum estimate of the number of *Attamyces* cells carried by a female *A. texana* in her pellet. Apart from the number of viable *Attamyces* cells per pellet, observed CFU counts are likely influenced by additional factors, such as (a) the particular plant substrate incorporated in the pellet (different substrates may anchor or protect *Attamyces* cells differently), (b) viability differences between *Attamyces* genotypes on the growth medium, and (c) pellet health or age (e.g., time between collection of females and experimental expulsion of the pellet in the lab). Absolute counts of CFUs per pellet and any differences between samples (e.g., between ant nests; Supporting Information Table S5) therefore need to be interpreted with caution, but the CFUs provide a minimum estimate of the number of cultivar cells carried by female *A. texana*.

### Microsatellite Marker Genotyping

*Attamyces* collections were genotyped with microsatellite markers developed for *Attamyces* cultivars of leafcutter ants [29]. Consistent with the multinucleate nature of *Attamyces* cells found in ultramorphological studies [69], [70], *Attamyces* fungi are genotypically polyploid, with up to 5 alleles per locus per individual [29]. Profiling of an *Attamyces* collection at 10–15 loci therefore yields information on the presence/absence of 70–100 markers. Slightly different microsatellite panels were used in the different genotyping analyses (e.g., panel of 10 loci versus panel of 15 loci), but these genotyping differences do not affect any of the conclusions. See Supporting Information for details of the genotyping methods in each specific analysis.

## Supporting Information

Results S1 Genotyping Methods and Results.
(0.06 MB DOC)

Table S1 *Attamyces* pellet genotyping 2004-2010 for *Atta texana*.
(0.18 MB PDF)

Table S2 Within-nest *Attamyces* diversity for *Atta texana*.
(0.07 MB PDF)

Table S3 Within-nest *Attamyces* diversity for *Atta cephalotes*.
(0.30 MB PDF)

Table S4 *Attamyces* garden genotyping 2004&2008 for *Atta cephalotes*.
(0.06 MB PDF)

Table S5 *Attamyces* colony-forming units per pellet for *Atta texana*.
(0.06 MB PDF)
