# Supplementary material for: Monoculture of Leafcutter Ant Gardens
Source: PLoS One. 2010 Sep 10;5(9):e12668. doi: 10.1371/journal.pone.0012668 (PMC2937030; doi:10.1371/journal.pone.0012668)
Supplement: Results S1 — Genotyping Methods and Results. (0.06 MB DOC) [file pone.0012668.s001.doc]

**Supporting Information, *Monoculture of Leafcutter Ant Gardens***

Ulrich G. Mueller, Jarrod J. Scott, Heather D. Ishak, Michael Cooper, Andre Rodrigues

**SUPPLEMENT S1: MICROSATELLITE DNA GENOTYPING & RESULTS**

**MICROSATELLITE DNA GENOTYPING**

*Attamyces* fungi were genotyped using microsatellite markers originally characterized by [1]. Different panels of loci were used to DNA-fingerprint the fungi (pellets, gardens), using different sets of most-informative loci for the Texas samples (11 or 12 loci, Tables S1 and S2) and Panama samples (10 or 15 loci, Tables S3 and S4). Later genotyping analyses covered more loci because we developed and improved primers for additional loci between the time of the first genotyping screens (2003/2004) and the time of the last genotyping screens (2009/2010). Within each screen, only one microsatellite panel was used. The differences in genotyping methodology between screens (e.g., screening of 10 loci versus 15 loci) therefore do not affect any of the conclusions emerging from each screen.

A small fragment of pure mycelium (free of garden substrate) or fungal staphylae (aggregation of hyphal-tip swellings typical for *Attamyces*) was picked under the microscope with flame-sterilized forceps for DNA extraction, as described in [1]. DNA was extracted from fungal tissue (mycelium or staphylae) by placing it in 100µl of a 10% Chelex (Sigma-Aldrich) solution at 60ºC for 1.5 hrs, followed by 10 minutes at 99ºC. One microliter of this extract was used as template in a 10 μl PCR amplification volume of microsatellite markers.

*Genotyping of fungi from pellets of* Atta texana

Fungi in pellets were genotyped on an ABI-3100 Capillary Genotyper using primers for eleven microsatellite markers originally described in [1] (Table S1 lists the eleven markers used). Six of the eleven markers were multiplexed as follows:

Loci A1030, B12, and C625: common annealing temperature T_m_ = 60 °Celsius.

Loci B150, C101, and C117: common annealing temperature T_m_ = 58 °Celsius.

Five additional loci (A1132, A1151, B319, B430, A128) were amplified and analyzed individually (not multiplexed). A more efficient multiplexing regime was developed later, but this improved method was not available when the *A. texana* pellets were analyzed in 2006 and 2007.

The multiplex PCR reaction contained 1X PCR buffer, 0.3125 mM of each dNTP, 5mM MgCl_2_, 10 μg BSA, 2 nmol of each primer, and 0.25 units of Taq polymerase. For the five markers analyzed individually, the ingredients for the 10µl PCR reaction were the same as for the multiplex reactions, except 0.2 mM of each dNTP and 2.5 mM MgCl_2_ were used. For all amplifications, the temperature profile involved an initial denaturing step of 94ºC for 5 minutes, followed by 35 cycles of 10 seconds denaturation, 15 seconds at the recommended annealing temperature [1], and 25 seconds of extension at 72ºC. The first 10 cycles used a denaturation step at 94ºC, the remaining denaturation steps were at 89ºC. A final extension step at 72ºC was run for 45 minutes. One microliter of the PCR product was added to 1.5 μl of size-standard (lab-made, following the methods of [2]) and 7.5 μl of HiDi formamide (Applied Biosystems), heated to 95ºC for 5 minutes, then cooled to 10ºC and analyzed on an ABI-3100 Genotyper. Microsatellite marker sizes were scored using GeneMarker v1.5 (Softgenetics, State College, PA).

The fungal pellets from 2008-2010 were genotyped with an improved multiplexing protocol with fully optimized primer combinations (Ishak et al. in preparation), and these 2008-2010 samples were compared with earlier samples genotyped anew with the improved protocol. Fungi of leafcutter ants are multinucleate, yielding between 3-5 alleles per locus [1]. Overall, screening of eleven loci yielded information on the presence/absence of 80 alleles.

*Genotyping of fungi from excavated gardens of* Atta cephalotes

Fungi were genotyped marker-by-marker (i.e., not multiplexed) following the basic protocol of [1], with the modification of a M13-tailed priming scheme to minimize expenses of fluorescently labeled primers. The M13-tailed scheme was adapted from [3] and [4]; specifically, the microsatellite forward primer was 5-tailed with the M13 forward sequence and used in conjunction with a 15-fold excess of a fluorescently labeled M13 forward primer during the amplification of microsatellite alleles. While the M13-tailed priming scheme reduced the expenses of purchasing fluorescently labeled microsatellite primers for each locus, the priming scheme also appeared to generate artifacts (e.g., increased stutter) and the priming scheme was abandoned in later microsatellite surveys. Unlike the survey of pellet fungi from *A. texana*, we did not conduct an error analysis through blind rescoring for the *A. cephalotes* fungi; we therefore are unable to fully assess the likelihood that the minimal differences between fungal samples from the same nest are due to PCR artifact (allele dropout), sporadic artifacts associated with M13-tailed priming, or mutation at the microsatellite loci. We assume that the error rate in our allele scoring is comparable (<1%; see next) between the pellet survey on *A. texana* and the garden survey on *A. cephalotes*.

*Scoring of multinucleate (polyploid) microsatellite profiles and quality control*

Leafcutter fungi are polyploid; an individual generally shows 2-4 (range 1-5) alleles per locus [1], consistent with the multinucleate nature of leafcutter-fungus cells found in previous ultramorphological studies [5, 6]. To assess scoring error of these markers, 88 *Attamyces* fungi from *A. texana* with unusual alleles were blindly re-genotyped (starting with de novo extraction of fungal mycelium) as part of a larger population-genetic analysis on *Attamyces* from *A. texana* (U.G. Mueller, A.S. Mikheyev, and S.S. Solomon unpublished); only 0.9% of 5130 alleles scored showed discrepancies between the initial scoring and the blind re-scoring. This low error rate therefore (a) rules out scoring artifacts as an explanation for the consistently multi-allelic microsatellite profiles per locus, and (b) documents the high reliability of our scoring scheme.

**RESULTS**

**1. Genotyping of fungal pellets carried by dispersing queens from the same nest of *Atta texana***

Although *Attamyces* genotypes differed between *Atta* nests, all fungal pellets stemming from a single nest showed identical microsatellite marker profiles (Table S1), with the exception of PCR-artifacts, as follows. Four samples (BFL1 #1-1 2006; BFL1#1-2 2006; BFL1 #1-41 2006; A-36 2006) did not amplify at any of the eleven loci, and these samples were therefore eliminated. In two samples (B-11 2006; B-23 2006), more than 50% of the loci did not amplify because of apparent PCR problems (e.g., insufficient template, PCR inhibitors), and these two samples also showed a high number of ‘missing alleles’ (bolded in Table S1) in the remaining loci; we attribute these ‘missing alleles’ in the remaining loci to PCR-problems, and we therefore do not interpret them as true genetic losses of alleles in these two problem samples. To substantiate this interpretation, we reanalyzed one such sample (A-49 2006) that had initial amplification products at all eleven loci but showed six ‘missing alleles’ (indicated by asterisks in the row of A-49 2006 in Table S1); in the repeat analysis (using the same PCR products but this time increasing the injection time in the ABI capillary genotyper), these ‘missing alleles’ were present (thus making this sample genetically identical to the other samples from that same nest). This indicates that the apparent ‘allele losses’ in the first analysis were due to PCR-problems (e.g., insufficient amplification producing weak peaks). Lastly, sporadic failures occurred occasionally at single loci (indicated as ‘fail’ in Table S1); these failures were generally due to a degraded capillary in the ABI 3100 automated capillary genotyper.

*Putative mutations*

Only two novel alleles appeared in two samples (allele 171 in locus B150 in sample BFL1 #1-30 2006; allele 168 in locus B150 in sample A-37 2006; these two novel alleles are highlighted in orange in Table S1). The novel alleles appeared as strong amplification products, and could be scored unambiguously by two experimenters (MC and UGM). The novel alleles could be due to PCR artifacts, or alternatively genetic mutation. An interpretation of PCR artifacts is supported by the observations that (a) both novel alleles occurred in the same locus (perhaps an error-prone locus); (b) the novel alleles were not one or two mutational steps removed from expected alleles, as would be most likely to occur under a stepwise mutational model of microsatellite repeat evolution; and (c) no expected allele ‘disappeared’ in the allele-profile that could have mutated into the novel allele. PCR-artifact therefore cannot be ruled out with absolute certainty as a possible explanation for the two novel alleles (among 1782 sampled loci; 162 samples, eleven loci screened per sample). On the other hand, mutation cannot be ruled out either. Assuming that (i) the screened fungi descended from the same ancestral fungus carried initially by the foundress queen of the nest (i.e., no genetic variation existed between *Attamyces* cells in the pellet), (ii) back mutations are negligible, and (iii) mutated and unmutated cell lines undergo cellular division at the same rate in a nest, the cellular-division mutation rate can be calculated from the total number of loci screened (1782 sampled loci) and from the proportion of cell lines encountered that showed a mutation at a locus. Assuming that the aforementioned two novel alleles were indeed mutations, the cellular-division mutation rate is 2/1782 = 1.12 x 10^-3^ per locus, which corresponds to the mutation rate expected for a dinucleotide microsatellite locus such as B150 [7-10].

**2. Resampling of pellet-cultivars carried by females from same *A. texana* nests**

In a longitudinal survey of mating flights of three *A. texana* nests between 2004-2010 (only one of these three nests was also studied under point 1. above), all *Attamyces* pellets carried by females from the same nest were genetically identical (screening at least three pellets from three females per nest per year; Supporting Information Table S1). Within each of the three *A. texana* nests screened, therefore, the *Attamyces* clones chosen by dispersing females for their pellets were genetically stable over six years.

**3. Genotyping of fungal gardens excavated from *Atta texana* nests in Texas**

We found no evidence of within-nest genetic diversity of *Attamyces* between excavated gardens of seven nests of *A. texana* (Supporting Information Table S2). Because few gardens were sampled per nest in *A. texana* (average of 3.7 gardens sampled/nest, range 2-8 gardens/nest), this result is less conclusive than the corresponding results for *A. cephalotes* (over 70 garden fragments sampled per nest; see next).

**4. Genotyping of fungal gardens excavated from *Atta cephalotes* nests in Panama**

Between the 438 *Attamyces* samples (10 loci screened per sample), sixteen PCR-amplifications failed completely (indicated as ‘fail’ in Table S3). These PCR-failures occurred sporadically in all loci (range of 0-3 PCR-failures between the 10 loci), and they appeared to be randomly distributed across the 4380 loci analyzed. We attribute these PCR failures to uncontrolled, non-systematic PCR problems (e.g., poor extraction, unique pipetting errors, contamination, or capillary degeneration in the sequencer). Because of the large number of successful amplifications (n=4364), these sixteen failed samples were not reanalyzed. In 28 amplifications, one expected allele did not amplify (allele dropout; highlighted in yellow in Table S3); 64% of these dropouts occurred in locus B150 where the two expected alleles differed in length by 10 base pairs. Locus B150 therefore appears to be particularly prone to allele dropout. In all but one case of allele dropout (97%), the longest expected allele failed to amplify, and allele dropout occurred 20 times (71%) in cases where the expected alleles differed in length by at least eight base pairs. These patterns of allele dropout across loci and allele-sizes suggest that the observed cases of allele dropout are due to PCR biases (failed amplification of the longer allele) rather than mutational loss of one allele (i.e., null allele because of a mutation in a priming site for the longer allele). Reduction in ploidy is also an unlikely explanation for allele dropout, because the maximum number of alleles dropping out in a sample was only two dropouts (observed in two samples) among the ten loci screened per sample, whereas ploidy reduction would presumably show allele dropouts at more than two loci. All cases of ‘missing’ alleles were therefore attributed to PCR artifacts.

*Putative mutations*

Novel alleles appeared in nine amplifications from eight samples collected from two nests (Colonies 6 & 8; highlighted in orange in Table S3). In Colony 6, one novel allele occurred at each of locus B312 and C117, and each of these novel alleles therefore could be the result of an independent mutation. In Colony 8, seven novel alleles occurred at three loci, one novel allele in locus A435, and three novel alleles each at locus A128 and locus A1132. Within each of locus A128 and locus A1132, the novel alleles were identical between samples. The novel alleles within each of locus A128 and locus A1132 therefore could have been derived from the same ancestral mutations. This suggests that there is evidence for only three putative mutations in Colony 8 (one mutation in each of the A128, A435, and A1132 loci). However, Colony 8 is also the nest with the largest number of allele dropouts, suggesting that some systematic PCR problems may have affected amplifications, particularly in the amplifications of the three loci A128, A1132, and B150, which together accounted for 91% of the combined cases of novel alleles and allele dropouts in Nest 8. A complex constellation of mutations and recombination could perhaps also explain the observed allele differences between the fungal samples in Colony 8, but systematic PCR problems or analytical problems (e.g., degraded capillaries in the automated genotyper when analyzing samples from this particular nest) cannot be ruled out as a complete explanations for the minor genotype differences between the samples. In summary, therefore, the most plausible number of putative mutations in the study on *Attamyces* from *Atta cephalotes* is five:

Nest 6: locus B312, mutation from marker 168 to 180, adding six dinucleotide repeats

Nest 6: locus C117, mutation from marker 263 to 266, adding one trinucleotide repeat

Nest 8: locus A128, mutation from marker 222 to 224, adding one dinucleotide repeat

Nest 8: locus A435, mutation from marker 234 to 232, loss of one dinucleotide repeat

Nest 8: locus A1132, mutation from 214 to 208, loss of two trinucleotide repeats

(Note: The mutations could theoretically also have been in the reverse directions, followed by spread of these mutational lineages through each respective colony). Mutations at five loci among 4380 loci screened translates into a cellular-division mutation rate of 1.14 x 10^-3^ (= 5/4380) mutations per microsatellite locus (assuming that each colony is founded with a single ancestral fungal genotype). When considering only the two most plausible mutations where one repeat was added to the microsatellite array (Nest 6, locus C117; Nest 8, locus A128), and ignoring the three other putative mutations as possible PCR artifacts, the cellular-division mutation rate changes to 4.56 x 10^-4^ (= 2/4380) per microsatellite locus. These estimates roughly encompass the mutation rates expected for di- and tri-nucleotide microsatellite loci in fungi [7-10].

Within the aforementioned caveats (screening of only six nests from a single locality in Panama; screening of only superficial gardens per nest; screening of only about 75 garden fragments from about 25 gardens per nest; screening of only 4380 loci in total), each *A. cephalotes* nest appears to cultivate *Attamyces* in monoculture.

**5. Resampling of cultivars from same *A. cephalotes* nests excavated in 2003 and 2008**

Using the novel multiplex method developed by Ishak et al. (in preparation), the comparison of genotype profiles from cultivar collections did not reveal any genotype changes within each *Atta cephalotes* colony between 2003 and 2008 (Table S4). The amplification failure-rate was slightly higher for the 2003 samples than for the 2008 samples (see ‘fail’ in Table S4), which we attribute to gradual DNA degradation in DMSO-salt buffer and storage over 5 years at -80°C, in comparison to the recently-collected and ethanol-preserved samples from 2008. Within each nest, we found no unambiguous differences between the collections obtained 5 years apart. One allele (allele 123, locus C101) was not consistently present in all of the four samples genotyped (two samples for each year), but the presence/absence differences for this marker occurred *within* each pair collected within a year, rather than between years, indicating that this difference affecting one allele is due to PCR artifacts, rather than coexistence of two minimally-differentiated genotypes over a period of five years. For all other alleles at the 15 loci, all markers were identical within each *A. cephalotes* colony sampled in 2003 and again in 2008, indicting that cultivar clones were stable within each colony over the 5-year period.

**6. Estimating the number of fungal cells in single pellets carried by females of *A. texana***

The average number of colony-forming units (CFUs) per pellet was 543.5 (StDev=355.2, n= 28, range 60-1500; Supporting Information Table S5). The average CFUs was higher for pellets from Nest 1 at Brackenridge Field Lab (average=600.8, StDev=371.1, n=20, range 60-1500) than for pellets from Nest A at Hornsby Bend (average=400.1, StDev=280.9, n=8, range 188-940), but this difference was not significant (two-tailed t-test for unequal sample sizes, p=0.14). Because most CFUs probably derive from aggregates of many *Attamyces* cells, the pellet which a female *A. texana* uses as a starter inoculum for her first garden probably contains a population of minimally several thousands of cultivar cells.

**References**

1. Scott JJ, Kweskin M, Cooper M, Mueller UG (2009) Polymorphic microsatellite markers for the symbiotic fungi (*Attamyces*) cultivated by leaf-cutter ants (Attini, Formicidae). Mol Ecol Resour 9: 1391-1394.

2. DeWoody JA, Schupp J, Kenefic L, Busch J, Murfitt L, Keim P (2004) Universal method for producing ROX-labeled size standards suitable for automated genotyping. BioTechniques 37: 348-352.

3. Boutin-Ganache I, Raposo M, Raymond M, Deschepper CFS (2001) M13-tailed primers improve the readability and usability of microsatellite analyses performed with two different allele-sizing methods. Biotechniques 31: 24-28.

4. Symonds VV, Lloyd AM (2003) An analysis of microsatellite loci in *Arabidopsis thaliana.* Genetics 165: 1475-1488.

5. Hervey A, Rogerson CT, Leong I (1977) Studies on fungi cultivated by ants. Brittonia 29: 226-236.

6. Mohali S (1998) Ultrastructural study of the mutualistic fungus of the ant *Atta cephalotes*. Rev Ecol Latinoamericana 5: 1-6.

7. Li,Y-C, Korol AB, Fahima T, Beiles A, Nevo E (2002) Microsatellites: genomic distribution, putative functions and mutational mechanisms: a review. Mol Ecol 11: 2453-2465.

8. Lim S, Notley-McRobb L, Lim M, Carter DA (2004) A comparison of the nature and abundance of microsatellites in 14 fungal genomes. Fungal Genet Biol 41: 1025-1036.

9. Dutech C, Enjalbert J, Fournier E, Delmotte F, Barres B, Carlier J, Tharreau D, Giraud T (2007) Challenges of microsatellite isolation in fungi. Fungal Genet Biol 44: 933-949.

10. Lynch M, Sung W, Morris K, Coffey N, Landry CR, Dopman EB, Dickinson WJ, Okamoto K, Kulkarni S, Hartl DL, *et al.* (2008) A genome-wide view of the spectrum of spontaneous mutations in yeast. Proc Natl Acad Sci USA 105: 9272-9277.
