# Supplementary material for: Monoculture of Leafcutter Ant Gardens
Source: PLoS One. 2010 Sep 10;5(9):e12668. doi: 10.1371/journal.pone.0012668 (PMC2937030; doi:10.1371/journal.pone.0012668)
Supplement: Table S1 — Attamyces pellet genotyping 2004-2010 for Atta texana. (0.18 MB PDF) [file pone.0012668.s002.pdf]

Table S1. Microsatellite marker profiles of *Attamyces* in fungal pellets carried by female *Atta texana*.

Female *A. texana* were collected from nest mounds on the morning of a mating flight. Females from three nests (UGM050509-01, UGM050509-02, UGM050509-07) were collected between 2004-2010. Females from two nests (UGM060121-01, UGM060121-02) were collected only in 2006. Infrabuccal pellets were expelled from females, and mycelium from each pellet was genotyped at 11 microsatellite DNA markers. Markers were scored blind (without knowledge of previous scoring in the repeat genotyping). 1 = marker present. 0 = marker absent. ? = unclear whether marker is present or absent. The label "fail" indicates that the analysis failed for the particular locus (e.g., because of amplification failure or column failure in the automated genotyper). Markers with asterisks did not appear in a first analysis, but appeared in a second analysis using more amplification products to improve marker detection. Markers in bold indicate suspect scorings in problem samples, possibly due to insufficient template or inhibitors in the marker amplification. Disregarding these amplification artifacts, within each nest all markers were identical for the multiple samples screened, with two exceptions (BFL1 #1-30 2006, A-37 2006) that each showed a novel marker (orange highlight).

**#1 Colony BFL**		**A1132 (TCA)$_n$**									**B12 (GA)$_n$**						**C101 (CAG)$_n$(CAA)$_n$**							**C117 (CAG)$_n$(/CAC)$_n$**					**B150 (CT)$_n$**												
**UGM050509-01**	**Year**	**199**	**202**	**205**	**208**	**211**	**214**	**217**	**220**	**223**	**231**	**232**	**234**	**236**	**238**	**240**	**100**	**103**	**106**	**109**	**112**	**115**	**118**	**239**	**242**	**245**	**248**	**251**	**161**	**163**	**165**	**166**	**168**	**171**	**173**	**175**	**177**	**179**	**181**	**184**	**186**
BFL1 #1-18 2004	**2004**	1	0	0	1	0	0	0	0	1	0	0	1	0	1	1	1	0	0	1	1	1	0	1	0	0	1	1	1	0	0	0	0	0	0	1	0	0	1	0	0
BFL1 #1-19 2004		fail	fail	fail	fail	fail	fail	fail	fail	fail	0	0	1	0	1	1	1	0	0	1	1	1	0	1	0	0	1	1	1	0	0	0	0	0	0	1	0	0	1	0	0
BFL1 #1-20 2004		fail	fail	fail	fail	fail	fail	fail	fail	fail	0	0	1	0	1	1	1	0	0	1	1	1	0	1	0	0	1	1	1	0	0	0	0	0	0	1	0	0	1	0	0
BFL1 #1-90 2005	**2005**	1	0	0	1	0	0	0	0	1	0	0	1	0	1	1	1	0	0	1	1	1	0	1	0	0	1	1	1	0	0	0	0	0	0	1	0	0	1	0	0
BFL1 #1-91 2005		1	0	0	1	0	0	0	0	1	0	0	1	0	1	1	1	0	0	1	1	1	0	1	0	0	1	1	1	0	0	0	0	0	0	1	0	0	1	0	0
BFL1 #1-92 2005		1	0	0	1	0	0	0	0	1	0	0	1	0	1	1	1	0	0	1	1	1	0	1	0	0	1	1	1	0	0	0	0	0	0	1	0	0	1	0	0
BFL1 #1-3 2006	**2006**	1	0	0	1	0	0	0	0	1	0	0	1	0	1	1	1	0	0	1	1	1	0	1	0	0	1	1	1	0	0	0	0	0	0	1	0	0	1	0	0
BFL1 #1-4 2006		1	0	0	1	0	0	0	0	1	0	0	1	0	1	1	1	0	0	1	1	1	0	1	0	0	1	1	1	0	0	0	0	0	0	1	0	0	1	0	0
BFL1 #1-5 2006		1	0	0	1	0	0	0	0	1	0	0	1	0	1	1	1	0	0	1	1	1	0	1	0	0	1	1	1	0	0	0	0	0	0	1	0	0	1	0	0
BFL1 #1-6 2006		1	0	0	1	0	0	0	0	1	0	0	1	0	1	1	1	0	0	1	1	1	0	1	0	0	1	1	1	0	0	0	0	0	0	1	0	0	1	0	0
BFL1 #1-7 2006		1	0	0	1	0	0	0	0	1	0	0	1	0	1	1	1	0	0	1	1	1	0	1	0	0	1	1	1	0	0	0	0	0	0	1	0	0	1	0	0
BFL1 #1-8 2006		1	0	0	1	0	0	0	0	1	0	0	1	0	1	1	1	0	0	1	1	1	0	1	0	0	1	1	1	0	0	0	0	0	0	1	0	0	1	0	0
BFL1 #1-9 2006		1	0	0	1	0	0	0	0	1	0	0	1	0	1	1	1	0	0	1	1	1	0	1	0	0	1	1	1	0	0	0	0	0	0	1	0	0	1	0	0
BFL1 #1-10 2006		1	0	0	1	0	0	0	0	1	0	0	1	0	1	1	1	0	0	1	1	1	0	1	0	0	1	1	1	0	0	0	0	0	0	1	0	0	1	0	0
BFL1 #1-11 2006		1	0	0	1	0	0	0	0	1	0	0	1	0	1	1	1	0	0	1	1	1	0	1	0	0	1	1	1	0	0	0	0	0	0	1	0	0	1	0	0
BFL1 #1-12 2006		1	0	0	1	0	0	0	0	1	0	0	1	0	1	1	1	0	0	1	1	1	0	1	0	0	1	1	1	0	0	0	0	0	0	1	0	0	1	0	0
BFL1 #1-13 2006		1	0	0	1	0	0	0	0	1	0	0	1	0	1	1	1	0	0	1	1	1	0	1	0	0	1	1	1	0	0	0	0	0	0	1	0	0	1	0	0
BFL1 #1-14 2006		1	0	0	1	0	0	0	0	1	0	0	1	0	1	1	1	0	0	1	1	1	0	1	0	0	1	1	1	0	0	0	0	0	0	1	0	0	1	0	0
BFL1 #1-15 2006		1	0	0	1	0	0	0	0	1	0	0	1	0	1	1	1	0	0	1	1	1	0	1	0	0	1	1	1	0	0	0	0	0	0	1	0	0	1	0	0
BFL1 #1-16 2006		1	0	0	1	0	0	0	0	1	0	0	1	0	1	1	1	0	0	1	1	1	0	1	0	0	1	1	1	0	0	0	0	0	0	1	0	0	1	0	0
BFL1 #1-17 2006		1	0	0	1	0	0	0	0	1	0	0	1	0	1	1	1	0	0	1	1	1	0	1	0	0	1	1	1	0	0	0	0	0	0	1	0	0	1	0	0
BFL1 #1-18 2006		1	0	0	1	0	0	0	0	1	0	0	1	0	1	1	1	0	0	1	1	1	0	1	0	0	1	1	1	0	0	0	0	0	0	1	0	0	1	0	0
BFL1 #1-19 2006		1	0	0	1	0	0	0	0	1	0	0	1	0	1	1	1	0	0	1	1	1	0	1	0	0	1	1	1	0	0	0	0	0	0	1	0	0	1	0	0
BFL1 #1-20 2006		1	0	0	1	0	0	0	0	1	0	0	1	0	1	1	1	0	0	1	1	1	0	1	0	0	1	1	1	0	0	0	0	0	0	1	0	0	1	0	0
BFL1 #1-21 2006		1	0	0	1	0	0	0	0	1	0	0	1	0	1	1	1	0	0	1	1	1	0	1	0	0	1	1	1	0	0	0	0	0	0	1	0	0	1	0	0
BFL1 #1-22 2006		1	0	0	1	0	0	0	0	1	0	0	1	0	1	1	1	0	0	1	1	1	0	1	0	0	1	1	1	0	0	0	0	0	0	1	0	0	1	0	0
BFL1 #1-23 2006		1	0	0	1	0	0	0	0	1	0	0	1	0	1	1	1	0	0	1	1	1	0	1	0	0	1	1	1	0	0	0	0	0	0	1	0	0	1	0	0
BFL1 #1-24 2006		1	0	0	1	0	0	0	0	1	0	0	1	0	1	1	1	0	0	1	1	1	0	1	0	0	1	1	1	0	0	0	0	0	0	1	0	0	1	0	0
BFL1 #1-25 2006		?	?	?	?	?	?	?	?	?	0	0	1	0	1	1	1	0	0	1	1	1	0	1	0	0	1	1	1	0	0	0	0	0	0	1	0	0	1	0	0
BFL1 #1-26 2006		1	0	0	1	0	0	0	0	1	0	0	1	0	1	1	1	0	0	1	1	1	0	1	0	0	1	1	1	0	0	0	0	0	0	1	0	0	1	0	0
BFL1 #1-27 2006		1	0	0	1	0	0	0	0	1	0	0	1	0	1	1	1	0	0	1	1	1	0	1	0	0	1	1	1	0	0	0	0	0	0	1	0	0	1	0	0
BFL1 #1-28 2006		1	0	0	1	0	0	0	0	1	0	0	1	0	1	1	1	0	0	1	1	1	0	1	0	0	1	1	1	0	0	0	0	0	0	1	0	0	1	0	0
BFL1 #1-29 2006		1	0	0	1	0	0	0	0	1	0	0	1	0	1	1	1	0	0	1	1	1	0	1	0	0	1	1	1	0	0	0	0	0	0	1	0	0	1	0	0
BFL1 #1-30 2006		1	0	0	1	0	0	0	0	1	0	0	1	0	1	1	1	0	0	1	1	1	0	1	0	0	1	1	1	0	0	0	0	**1**	0	1	0	0	1	0	0
BFL1 #1-31 2006		fail	fail	fail	fail	fail	fail	fail	fail	fail	0	0	1	0	1	1	1	0	0	1	1	1	0	1	0	0	1	1	1	0	0	0	0	0	0	1	0	0	1	0	0
BFL1 #1-32 2006		1	0	0	1	0	0	0	0	1	0	0	1	0	1	1	1	0	0	1	1	1	0	1	0	0	1	1	1	0	0	0	0	0	0	1	0	0	1	0	0
BFL1 #1-33 2006		1	0	0	1	0	0	0	0	1	fail	fail	fail	fail	fail	fail	1	0	0	1	1	1	0	1	0	0	1	1	1	0	0	0	0	0	0	1	0	0	1	0	0
BFL1 #1-34 2006		1	0	0	1	0	0	0	0	1	0	0	1	0	1	1	1	0	0	1	1	1	0	1	0	0	1	1	1	0	0	0	0	0	0	1	0	0	1	0	0
BFL1 #1-35 2006		1	0	0	1	0	0	0	0	1	0	0	1	0	1	1	1	0	0	1	1	1	0	1	0	0	1	1	1	0	0	0	0	0	0	1	0	0	1	0	0
BFL1 #1-36 2006		1	0	0	1	0	0	0	0	1	0	0	1	0	1	1	1	0	0	1	1	1	0	1	0	0	1	1	1	0	0	0	0	0	0	1	0	0	1	0	0
BFL1 #1-37 2006		1	0	0	1	0	0	0	0	1	0	0	1	0	1	1	1	0	0	1	1	1	0	1	0	0	1	1	1	0	0	0	0	0	0	1	0	0	1	0	0
BFL1 #1-38 2006		1	0	0	1	0	0	0	0	1	0	0	1	0	1	1	1	0	0	1	1	1	0	1	0	0	1	1	1	0	0	0	0	0	0	1	0	0	1	0	0
BFL1 #1-39 2006		1	0	0	1	0	0	0	0	1	0	0	1	0	1	1	1	0	0	1	1	1	0	1	0	0	1	1	1	0	0	0	0	0	0	1	0	0	1	0	0
BFL1 #1-40 2006		1	0	0	1	0	0	0	0	1	0	0	1	0	1	1	1	0	0	1	1	1	0	1	0	0	1	1	1	0	0	0	0	0	0	1	0	0	1	0	0
BFL1 #1-42 2006		1	0	0	1	0	0	0	0	1	0	0	1	0	1	1	1	0	0	1	1	1	0	1	0	0	1	1	1	0	0	0	0	0	0	1	0	0	1	0	0
BFL1 #1-43 2006		1	0	0	1	0	0	0	0	1	0	0	1	0	1	1	1	0	0	1	1	1	0	1	0	0	1	1	1	0	0	0	0	0	0	1	0	0	1	0	0
BFL1 #1-44 2006		1	0	0	1	0	0	0	0	1	0	0	1	0	1	1	1	0	0	1	1	1	0	1	0	0	1	1	1	0	0	0	0	0	0	1	0	0	1	0	0
BFL1 #1-45 2006		1	0	0	1	0	0	0	0	1	0	0	1	0	1	1	1	0	0	1	1	1	0	1	0	0	1	1	1	0	0	0	0	0	0	1	0	0	1	0	0
BFL1 #1-46 2006		1	0	0	1	0	0	0	0	1	0	0	1	0	1	1	1	0	0	1	1	1	0	1	0	0	1	1	1	0	0	0	0	0	0	1	0	0	1	0	0
BFL1 #1-47 2006		1	0	0	1	0	0	0	0	1	0	0	1	0	1	1	1	0	0	1	1	1	0	1	0	0	1	1	1	0	0	0	0	0	0	1	0	0	1	0	0
BFL1 #1a 2007	**2007**	1	0	0	1	0	0	0	0	1	0	0	1	0	1	1	1	0	0	1	1	1	0	1	0	0	1	1	1	0	0	0	0	0	0	1	0	0	1	0	0
BFL1 #1b 2007		1	0	0	1	0	0	0	0	1	0	0	1	0	1	1	1	0	0	1	1	1	0	1	0	0	1	1	1	0	0	0	0	0	0	1	0	0	1	0	0

BFL1 #1c 2007		1	0	0	1	0	0	0	0	1	0	0	1	0	1	1	1	0	0	1	1	1	0	1	0	0	1	1	1	0	0	0	0	0	0	1	0	0	1	0	0
BFL1 #1a 2008	2008	1	0	0	1	0	0	0	0	1	0	0	1	0	1	1	1	0	0	1	1	1	0	1	0	0	1	1	1	0	0	0	0	0	0	1	0	0	1	0	0
BFL1 #1b 2008		1	0	0	1	0	0	0	0	1	0	0	1	0	1	1	1	0	0	1	1	1	0	1	0	0	1	1	1	0	0	0	0	0	0	1	0	0	1	0	0
BFL1 #1c 2008		1	0	0	1	0	0	0	0	1	0	0	1	0	1	1	1	0	0	1	1	1	0	1	0	0	1	1	1	0	0	0	0	0	0	1	0	0	1	0	0
BFL1 #1a 2009	2009	1	0	0	1	0	0	0	0	1	0	0	1	0	1	1	1	0	0	1	1	1	0	1	0	0	1	1	1	0	0	0	0	0	0	1	0	0	1	0	0
BFL1 #1b 2009		1	0	0	1	0	0	0	0	1	0	0	1	0	1	1	1	0	0	1	1	1	0	1	0	0	1	1	1	0	0	0	0	0	0	1	0	0	1	0	0
BFL1 #1c 2009		1	0	0	1	0	0	0	0	1	0	0	1	0	1	1	1	0	0	1	1	1	0	1	0	0	1	1	1	0	0	0	0	0	0	1	0	0	1	0	0
BFL1 #1a 2010	2010	1	0	0	1	0	0	0	0	1	0	0	1	0	1	1	1	0	0	1	1	1	0	1	0	0	1	1	1	0	0	0	0	0	0	1	0	0	1	0	0
BFL1 #1b 2010		1	0	0	1	0	0	0	0	1	0	0	1	0	1	1	1	0	0	1	1	1	0	1	0	0	1	1	1	0	0	0	0	0	0	1	0	0	1	0	0
BFL1 #1c 2010		1	0	0	1	0	0	0	0	1	0	0	1	0	1	1	1	0	0	1	1	1	0	1	0	0	1	1	1	0	0	0	0	0	0	1	0	0	1	0	0

#2 Colony BFL		A1132 (TCA)$_n$									B12 (GA)$_n$						C101 (CAG)$_n$(CAA)$_n$							C117 (CAG)$_n$(/CAC)$_n$					B150 (CT)$_n$												
**UGM050509-02**	**Year**	**199**	**202**	**205**	**208**	**211**	**214**	**217**	**220**	**223**	**231**	**232**	**234**	**236**	**238**	**240**	**100**	**103**	**106**	**109**	**112**	**115**	**118**	**239**	**242**	**245**	**248**	**251**	**161**	**163**	**165**	**166**	**168**	**171**	**173**	**175**	**177**	**179**	**181**	**184**	**186**
BFL2 #2-81 2004	2004	1	1	0	0	0	0	0	1	0	0	0	1	0	1	0	0	0	0	0	1	1	1	0	0	0	1	1	1	0	0	0	0	0	0	1	0	1	0	0	0
BFL2 #2-82 2004		1	1	0	0	0	0	0	1	0	0	0	1	0	1	0	0	0	0	0	1	1	1	0	0	0	1	1	1	0	0	0	0	0	0	1	0	1	0	0	0
BFL2 #2-84 2004		1	1	0	0	0	0	0	1	0	0	0	1	0	1	0	0	0	0	0	1	1	1	0	0	0	1	1	1	0	0	0	0	0	0	1	0	1	0	0	0
BFL2 #2a 2007	2007	1	1	0	0	0	0	0	1	0	0	0	1	0	1	0	0	0	0	0	1	1	1	0	0	0	1	1	1	0	0	0	0	0	0	1	0	1	0	0	0
BFL2 #2b 2007		1	1	0	0	0	0	0	1	0	0	0	1	0	1	0	0	0	0	0	1	1	1	0	0	0	1	1	1	0	0	0	0	0	0	1	0	1	0	0	0
BFL2 #2c 2007		1	1	0	0	0	0	0	1	0	0	0	1	0	1	0	0	0	0	0	1	1	1	0	0	0	1	1	1	0	0	0	0	0	0	1	0	1	0	0	0
BFL2 #2a 2008	2008	1	1	0	0	0	0	0	1	0	0	0	1	0	1	0	0	0	0	0	1	1	1	0	0	0	1	1	1	0	0	0	0	0	0	1	0	1	0	0	0
BFL2 #2b 2008		1	1	0	0	0	0	0	1	0	0	0	1	0	1	0	0	0	0	0	1	1	1	0	0	0	1	1	1	0	0	0	0	0	0	1	0	1	0	0	0
BFL2 #2c 2008		1	1	0	0	0	0	0	1	0	0	0	1	0	1	0	0	0	0	0	1	1	1	0	0	0	1	1	1	0	0	0	0	0	0	1	0	1	0	0	0
BFL2 #2a 2009	2009	1	1	0	0	0	0	0	1	0	0	0	1	0	1	0	0	0	0	0	1	1	1	0	0	0	1	1	1	0	0	0	0	0	0	1	0	1	0	0	0
BFL2 #2b 2009		1	1	0	0	0	0	0	1	0	0	0	1	0	1	0	0	0	0	0	1	1	1	0	0	0	1	1	1	0	0	0	0	0	0	1	0	1	0	0	0
BFL2 #2c 2009		1	1	0	0	0	0	0	1	0	0	0	1	0	1	0	0	0	0	0	1	1	1	0	0	0	1	1	1	0	0	0	0	0	0	1	0	1	0	0	0
BFL2 #2a 2010	2010	1	1	0	0	0	0	0	1	0	0	0	1	0	1	0	0	0	0	0	1	1	1	0	0	0	1	1	1	0	0	0	0	0	0	1	0	1	0	0	0
BFL2 #2b 2010		1	1	0	0	0	0	0	1	0	0	0	1	0	1	0	0	0	0	0	1	1	1	0	0	0	1	1	1	0	0	0	0	0	0	1	0	1	0	0	0
BFL2 #2c 2010		1	1	0	0	0	0	0	1	0	0	0	1	0	1	0	0	0	0	0	1	1	1	0	0	0	1	1	1	0	0	0	0	0	0	1	0	1	0	0	0

#7 Colony BFL		A1132 (TCA)$_n$									B12 (GA)$_n$						C101 (CAG)$_n$(CAA)$_n$							C117 (CAG)$_n$(/CAC)$_n$					B150 (CT)$_n$												
**UGM050509-07**	**Year**	**199**	**202**	**205**	**208**	**211**	**214**	**217**	**220**	**223**	**231**	**232**	**234**	**236**	**238**	**240**	**100**	**103**	**106**	**109**	**112**	**115**	**118**	**239**	**242**	**245**	**248**	**251**	**161**	**163**	**165**	**166**	**168**	**171**	**173**	**175**	**177**	**179**	**181**	**184**	**186**
BFL7 #2a 2004	2004	1	0	0	1	0	0	0	0	1	0	0	1	0	1	1	1	0	0	1	1	1	0	1	0	0	1	1	1	0	0	0	0	0	0	1	0	0	1	0	0
BFL7 #2b 2004		1	0	0	1	0	0	0	0	1	0	0	1	0	1	1	1	0	0	1	1	1	0	1	0	0	1	1	1	0	0	0	0	0	0	1	0	0	1	0	0
BFL7 #2a 2009	2009	1	0	0	1	0	0	0	0	1	0	0	1	0	1	1	fail	fail	fail	fail	fail	fail	fail	1	0	0	1	1	1	0	0	0	0	0	0	1	0	0	1	0	0
BFL7 #2b 2009		1	0	0	1	0	0	0	0	1	0	0	1	0	1	1	1	0	0	1	1	1	0	1	0	0	1	1	1	0	0	0	0	0	0	1	0	0	1	0	0
BFL7 #2c 2009		1	0	0	1	0	0	0	0	1	0	0	1	0	1	1	1	0	0	1	1	1	0	1	0	0	1	1	1	0	0	0	0	0	0	1	0	0	1	0	0
BFL7 #2a 2010	2010	1	0	0	1	0	0	0	0	1	0	0	1	0	1	1	1	0	0	1	1	1	0	1	0	0	1	1	1	0	0	0	0	0	0	1	0	0	1	0	0

A-Colony Hornsby		A1132 (TCA)$_n$									B12 (GA)$_n$						C101 (CAG)$_n$(CAA)$_n$							C117 (CAG)$_n$(/CAC)$_n$					B150 (CT)$_n$												
**UGM060121-1**	**Year**	**199**	**202**	**205**	**208**	**211**	**214**	**217**	**220**	**223**	**231**	**232**	**234**	**236**	**238**	**240**	**100**	**103**	**106**	**109**	**112**	**115**	**118**	**239**	**242**	**245**	**248**	**251**	**161**	**163**	**165**	**166**	**168**	**171**	**173**	**175**	**177**	**179**	**181**	**184**	**186**
A-1 2006	2006	1	1	0	0	0	0	0	1	0	0	0	1	0	1	0	0	0	0	0	1	1	1	0	0	0	1	1	1	0	0	0	0	0	0	1	0	1	0	0	0
A-2 2006		1	1	0	0	0	0	0	1	0	0	0	1	0	1	0	0	0	0	0	1	1	1	0	0	0	1	1	1	0	0	0	0	0	0	1	0	1	0	0	0
A-3 2006		1	1	0	0	0	0	0	1	0	0	0	1	0	1	0	0	0	0	0	1	1	1	0	0	0	1	1	1	0	0	0	0	0	0	1	0	1	0	0	0
A-20 2006		1	1	0	0	0	0	0	1	0	0	0	1	0	1	0	0	0	0	0	1	1	1	0	0	0	1	1	1	0	0	0	0	0	0	1	0	1	0	0	0
A-21 2006		1	1	0	0	0	0	0	1	0	0	0	1	0	1	0	0	0	0	0	1	1	1	fail	fail	fail	fail	fail	1	0	0	0	0	0	0	1	0	1	0	0	0
A-22 2006		1	1	0	0	0	0	0	1	0	0	0	1	0	1	0	0	0	0	0	1	1	1	0	0	0	1	1	1	0	0	0	0	0	0	1	0	1	0	0	0
A-23 2006		1	1	0	0	0	0	0	1	0	0	0	1	0	1	0	0	0	0	0	1	1	1	0	0	0	1	1	1	0	0	0	0	0	0	1	0	1	0	0	0
A-24 2006		1	1	0	0	0	0	0	1	0	0	0	1	0	1	0	0	0	0	0	1	1	1	fail	fail	fail	fail	fail	1	0	0	0	0	0	0	1	0	1	0	0	0
A-25 2006		1	1	0	0	0	0	0	1	0	0	0	1	0	1	0	0	0	0	0	1	1	1	0	0	0	1	1	1	0	0	0	0	0	0	1	0	1	0	0	0
A-26 2006		1	1	0	0	0	0	0	1	0	0	0	1	0	1	0	0	0	0	0	1	1	1	0	0	0	1	1	1	0	0	0	0	0	0	1	0	1	0	0	0
A-27 2006		1	1	0	0	0	0	0	1	0	0	0	1	0	1	0	0	0	0	0	1	1	1	0	0	0	1	1	1	0	0	0	0	0	0	1	0	1	0	0	0
A-28 2006		1	1	0	0	0	0	0	1	0	0	0	1	0	1	0	0	0	0	0	1	1	1	0	0	0	1	1	1	0	0	0	0	0	0	1	0	1	0	0	0
A-29 2006		1	1	0	0	0	0	0	1	0	0	0	1	0	1	0	0	0	0	0	1	1	1	0	0	0	1	1	1	0	0	0	0	0	0	1	0	1	0	0	0
A-30 2006		1	1	0	0	0	0	0	1	0	0	0	1	0	1	0	0	0	0	0	1	1	1	0	0	0	1	1	1	0	0	0	0	0	0	1	0	1	0	0	0
A-31 2006		1	1	0	0	0	0	0	1	0	0	0	1	0	1	0	0	0	0	0	1	1	1	0	0	0	1	1	1	0	0	0	0	0	0	1	0	1	0	0	0
A-32 2006		1	1	0	0	0	0	0	1	0	0	0	1	0	1	0	0	0	0	0	1	1	1	0	0	0	1	1	1	0	0	0	0	0	0	1	0	1	0	0	0
A-33 2006		1	1	0	0	0	0	0	1	0	0	0	1	0	1	0	0	0	0	0	1	1	1	0	0	0	1	1	1	0	0	0	0	0	0	1	0	1	0	0	0
A-34 2006		1	1	0	0	0	0	0	1	0	0	0	1	0	1	0	0	0	0	0	1	1	1	0	0	0	1	1	1	0	0	0	0	0	0	1	0	1	0	0	0
A-35 2006		1	1	0	0	0	0	0	1	0	0	0	1	0	1	0	0	0	0	0	1	1	1	0	0	0	1	1	1	0	0	0	0	0	0	1	0	1	0	0	0
A-37 2006		1	1	0	0	0	0	0	1	0	0	0	1	0	1	0	0	0	0	0	1	1	1	0	0	0	1	1	1	0	0	0	1	0	0	1	0	1	0	0	0

A-38 2006		1	1	0	0	0	0	0	1	0	0	0	1	0	1	0	0	0	0	0	1	1	1	0	0	0	1	1	1	0	0	0	0	0	0	1	0	1	0	0	0
A-39 2006		1	1	0	0	0	0	0	1	0	0	0	1	0	1	0	0	0	0	0	1	1	1	0	0	0	1	1	1	0	0	0	0	0	0	1	0	1	0	0	0
A-40 2006		1	1	0	0	0	0	0	1	0	0	0	1	0	1	0	0	0	0	0	1	1	1	0	0	0	1	1	1	0	0	0	0	0	0	1	0	1	0	0	0
A-41 2006		1	1	0	0	0	0	0	1	0	0	0	1	0	1	0	0	0	0	0	1	1	1	0	0	0	1	1	1	0	0	0	0	0	0	1	0	1	0	0	0
A-42 2006		1	1	0	0	0	0	0	1	0	0	0	1	0	1	0	0	0	0	0	1	1	1	0	0	0	1	1	1	0	0	0	0	0	0	1	0	1	0	0	0
A-43 2006		1	1	0	0	0	0	0	1	0	0	0	1	0	1	0	0	0	0	0	1	1	1	0	0	0	1	1	1	0	0	0	0	0	0	1	0	1	0	0	0
A-44 2006		1	1	0	0	0	0	0	1	0	0	0	1	0	1	0	0	0	0	0	1	1	1	0	0	0	1	1	1	0	0	0	0	0	0	1	0	1	0	0	0
A-45 2006		1	1	0	0	0	0	0	1	0	0	0	1	0	1	0	0	0	0	0	1	1	1	0	0	0	1	1	1	0	0	0	0	0	0	1	0	1	0	0	0
A-46 2006		1	1	0	0	0	0	0	1	0	0	0	1	0	1	0	0	0	0	0	1	1	1	0	0	0	1	1	1	0	0	0	0	0	0	1	0	1	0	0	0
A-47 2006		1	1	0	0	0	0	0	1	0	0	0	1	0	1	0	0	0	0	0	1	1	1	0	0	0	1	1	1	0	0	0	0	0	0	1	0	1	0	0	0
A-48 2006		1	1	0	0	0	0	0	1	0	0	0	1	0	1	0	0	0	0	0	1	1	1	0	0	0	1	1	1	0	0	0	0	0	0	1	0	1	0	0	0
A-49 2006		1	1	0	0	0	0	0	1*	0	0	0	1*	0	1	0	0	0	0	0	1	1*	1*	0	0	0	1	1	1*	0	0	0	0	0	0	1	0	1	0	0	0
A-50 2006		1	1	0	0	0	0	0	1	0	0	0	1	0	1	0	0	0	0	0	1	1	1	0	0	0	1	1	1	0	0	0	0	0	0	1	0	1	0	0	0
A-51 2006		1	1	0	0	0	0	0	1	0	0	0	1	0	1	0	0	0	0	0	1	1	1	0	0	0	1	1	1	0	0	0	0	0	0	1	0	1	0	0	0
A-52 2006		1	1	0	0	0	0	0	1	0	0	0	1	0	1	0	0	0	0	0	1	1	1	0	0	0	1	1	1	0	0	0	0	0	0	1	0	1	0	0	0
A-53 2006		1	1	0	0	0	0	0	1	0	0	0	1	0	1	0	0	0	0	0	1	1	1	0	0	0	1	1	1	0	0	0	0	0	0	1	0	1	0	0	0
A-54 2006		1	1	0	0	0	0	0	1	0	0	0	1	0	1	0	0	0	0	0	1	1	1	0	0	0	1	1	1	0	0	0	0	0	0	1	0	1	0	0	0
A-55 2006		1	1	0	0	0	0	0	1	0	0	0	1	0	1	0	0	0	0	0	1	1	1	0	0	0	1	1	1	0	0	0	0	0	0	1	0	1	0	0	0
A-56 2006		1	1	0	0	0	0	0	1	0	0	0	1	0	1	0	0	0	0	0	1	1	1	0	0	0	1	1	1	0	0	0	0	0	0	1	0	1	0	0	0
A-57 2006		1	1	0	0	0	0	0	1	0	0	0	1	0	1	0	0	0	0	0	1	1	1	0	0	0	1	1	1	0	0	0	0	0	0	1	0	1	0	0	0
A-58 2006		1	1	0	0	0	0	0	1	0	0	0	1	0	1	0	0	0	0	0	1	1	1	0	0	0	1	1	1	0	0	0	0	0	0	1	0	1	0	0	0
A-59 2006		1	1	0	0	0	0	0	1	0	0	0	1	0	1	0	0	0	0	0	1	1	1	0	0	0	1	1	1	0	0	0	0	0	0	1	0	1	0	0	0
A-60 2006		1	1	0	0	0	0	0	1	0	0	0	1	0	1	0	0	0	0	0	1	1	1	0	0	0	1	1	1	0	0	0	0	0	0	1	0	1	0	0	0
A-61 2006		1	1	0	0	0	0	0	1	0	0	0	1	0	1	0	0	0	0	0	1	1	1	0	0	0	1	1	1	0	0	0	0	0	0	1	0	1	0	0	0
A-62 2006		1	1	0	0	0	0	0	1	0	0	0	1	0	1	0	0	0	0	0	1	1	1	0	0	0	1	1	1	0	0	0	0	0	0	1	0	1	0	0	0
A-63 2006		1	1	0	0	0	0	0	1	0	0	0	1	0	1	0	0	0	0	0	1	1	1	0	0	0	1	1	1	0	0	0	0	0	0	1	0	1	0	0	0
A-64 2006		1	1	0	0	0	0	0	1	0	0	0	1	0	1	0	0	0	0	0	1	1	1	0	0	0	1	1	1	0	0	0	0	0	0	1	0	1	0	0	0
A-65 2006		1	1	0	0	0	0	0	1	0	0	0	1	0	1	0	0	0	0	0	1	1	1	0	0	0	1	1	1	0	0	0	0	0	0	1	0	1	0	0	0

**B-Colony Hornsby**		**A1132 $(TCA)_n$**									**B12 $(GA)_n$**						**C101 $(CAG)_n(CAA)_n$**							**C117 $(CAG)_n(/CAC)_n$**					**B150 $(CT)_n$**												
**UGM060121-02**	**Year**	**199**	**202**	**205**	**208**	**211**	**214**	**217**	**220**	**223**	**231**	**232**	**234**	**236**	**238**	**240**	**100**	**103**	**106**	**109**	**112**	**115**	**118**	**239**	**242**	**245**	**248**	**251**	**161**	**163**	**165**	**166**	**168**	**171**	**173**	**175**	**177**	**179**	**181**	**184**	**186**
B-1 2006	**2006**	1	0	0	1	0	1	1	0	0	1	0	1	0	1	0	0	0	1	0	1	1	0	1	0	0	1	1	1	0	0	0	0	0	0	1	0	0	1	0	1
B-2 2006		fail	fail	fail	fail	fail	fail	fail	fail	fail	1	0	1	0	1	0	0	0	1	0	1	1	0	1	0	0	1	1	1	0	0	0	0	0	0	1	0	0	1	0	1
B-3 2006		1	0	0	1	0	1	1	0	0	1	0	1	0	1	0	0	0	1	0	1	1	0	1	0	0	1	1	1	0	0	0	0	0	0	1	0	0	1	0	1
B-4 2006		1	0	0	1	0	1	1	0	0	1	0	1	0	1	0	0	0	1	0	1	1	0	1	0	0	1	1	1	0	0	0	0	0	0	1	0	0	1	0	1
B-5 2006		1	0	0	1	0	1	1	0	0	1	0	1	0	1	0	0	0	1	0	1	1	0	1	0	0	1	1	1	0	0	0	0	0	0	1	0	0	1	0	1
B-6 2006		1	0	0	1	0	1	1	0	0	1	0	1	0	1	0	0	0	1	0	1	1	0	1	0	0	1	1	1	0	0	0	0	0	0	1	0	0	1	0	1
B-7 2006		1	0	0	1	0	1	1	0	0	1	0	1	0	1	0	0	0	1	0	1	1	0	1	0	0	1	1	1	0	0	0	0	0	0	1	0	0	1	0	1
B-8 2006		1	0	0	1	0	1	1	0	0	1	0	1	0	1	0	0	0	1	0	1	1	0	1	0	0	1	1	1	0	0	0	0	0	0	1	0	0	1	0	1
B-9 2006		1	0	0	1	0	1	1	0	0	1	0	1	0	1	0	fail	fail	fail	fail	fail	fail	fail	fail	fail	fail	fail	fail	fail	fail	fail	fail	fail	fail	fail	fail	fail	fail	fail	fail	fail
B-10 2006		1	0	0	1	0	1	1	0	0	1	0	1	0	1	0	0	0	1	0	1	1	0	1	0	0	1	1	1	0	0	0	0	0	0	1	0	0	1	0	1
B-11 2006		fail	fail	fail	fail	fail	fail	fail	fail	fail	fail	fail	fail	fail	fail	fail	fail	fail	fail	fail	fail	fail	fail	fail	fail	fail	fail	fail	1	0	0	0	0	0	0	1	0	0	**0**	0	1
B-12 2006		1	0	0	1	0	1	1	0	0	1	0	1	0	1	0	0	0	1	0	1	1	0	1	0	0	1	1	1	0	0	0	0	0	0	1	0	0	1	0	1
B-13 2006		1	0	0	1	0	1	1	0	0	1	0	1	0	1	0	0	0	1	0	1	1	0	1	0	0	1	1	1	0	0	0	0	0	0	1	0	0	1	0	1
B-14 2006		1	0	0	1	0	1	1	0	0	1	0	1	0	1	0	0	0	1	0	1	1	0	1	0	0	1	1	1	0	0	0	0	0	0	1	0	0	1	0	1
B-15 2006		1	0	0	1	0	1	1	0	0	1	0	1	0	1	0	0	0	1	0	1	1	0	1	0	0	1	1	1	0	0	0	0	0	0	1	0	0	1	0	1
B-20 2006		1	0	0	1	0	1	1	0	0	1	0	1	0	1	0	0	0	1	0	1	1	0	1	0	0	1	1	1	0	0	0	0	0	0	1	0	0	1	0	1
B-21 2006		1	0	0	1	0	1	1	0	0	1	0	1	0	1	0	0	0	1	0	1	1	0	1	0	0	1	1	1	0	0	0	0	0	0	1	0	0	1	0	1
B-22 2006		1	0	0	1	0	1	1	0	0	1	0	1	0	1	0	0	0	1	0	1	1	0	1	0	0	1	1	1	0	0	0	0	0	0	1	0	0	1	0	1
B-23 2006		fail	fail	fail	fail	fail	fail	fail	fail	fail	fail	fail	fail	fail	fail	fail	fail	fail	fail	fail	fail	fail	fail	fail	fail	fail	fail	fail	1	0	0	0	0	0	0	**0**	0	**1**	1	0	**0**
B-24 2006		1	0	0	1	0	1	1	0	0	1	0	1	0	1	0	0	0	1	0	1	1	0	1	0	0	1	1	1	0	0	0	0	0	0	1	0	0	1	0	1
B-25 2006		1	0	0	1	0	1	1	0	0	1	0	1	0	1	0	0	0	1	0	1	1	0	1	0	0	1	1	1	0	0	0	0	0	0	1	0	0	1	0	1
B-26 2006		1	0	0	1	0	1	1	0	0	1	0	1	0	1	0	0	0	1	0	1	1	0	1	0	0	1	1	1	0	0	0	0	0	0	1	0	0	1	0	1
B-27 2006		1	0	0	1	0	1	1	0	0	1	0	1	0	1	0	0	0	1	0	1	1	0	1	0	0	1	1	1	0	0	0	0	0	0	1	0	0	1	0	1
B-28 2006		1	0	0	1	0	1	1	0	0	1	0	1	0	1	0	0	0	1	0	1	1	0	1	0	0	1	1	1	0	0	0	0	0	0	1	0	0	1	0	1
B-29 2006		1	0	0	1	0	1	1	0	0	1	0	1	0	1	0	0	0	1	0	1	1	0	1	0	0	1	1	1	0	0	0	0	0	0	1	0	0	1	0	1
B-30 2006		1	0	0	1	0	1	1	0	0	1	0	1	0	1	0	0	0	1	0	1	1	0	1	0	0	1	1	1	0	0	0	0	0	0	1	0	0	1	0	1
B-31 2006		1	0	0	1	0	1	1	0	0	1	0	1	0	1	0	0	0	1	0	1	1	0	1	0	0	1	1	1	0	0	0	0	0	0	1	0	0	1	0	1
B-32 2006		1	0	0	1	0	1	1	0	0	1	0	1	0	1	0	0	0	1	0	1	1	0	1	0	0	1	1	1	0	0	0	0	0	0	1	0	0	1	0	1
B-33 2006		1	0	0	1	0	1	1	0	0	1	0	1	0	1	0	0	0	1	0	1	1	0	1	0	0	1	1	1	0	0	0	0	0	0	1	0	0	1	0	1
B-34 2006		1	0	0	1	0	1	1	0	0	1	0	1	0	1	0	0	0	1	0	1	1	0	1	0	0	1	1	1	0	0	0	0	0	0	1	0	0	1	0	1
B-35 2006		1	0	0	1	0	1	1	0	0	1	0	1	0	1	0	0	0	1	0	1	1	0	1	0	0	1	1	1	0	0	0	0	0	0	1	0	0	1	0	1
B-36 2006		1	0	0	1	0	1	1	0	0	1	0	1	0	1	0	0	0	1	0	1	1	0	1	0	0	1	1	1	0	0	0	0	0	0	1	0	0	1	0	1
B-37 2006		1	0	0	1	0	1	1	0	0	1	0	1	0	1	0	0	0	1	0	1	1	0	1	0	0	1	1	1	0	0	0	0	0	0	1	0	0	1	0	1

B-38 2006		1	0	0	1	0	1	1	0	0	1	0	1	0	1	0	0	0	1	0	1	1	0	1	0	0	1	1	1	0	0	0	0	0	0	1	0	0	1	0	1
B-39 2006		1	0	0	1	0	1	1	0	0	1	0	1	0	1	0	0	0	1	0	1	1	0	1	0	0	1	1	1	0	0	0	0	0	0	1	0	0	1	0	1
B-40 2006		1	0	0	1	0	1	1	0	0	1	0	1	0	1	0	0	0	1	0	1	1	0	1	0	0	1	1	1	0	0	0	0	0	0	1	0	0	1	0	1
B-41 2006		1	0	0	1	0	1	1	0	0	1	0	1	0	1	0	0	0	1	0	1	1	0	1	0	0	1	1	1	0	0	0	0	0	0	1	0	0	1	0	1
B-42 2006		1	0	0	1	0	1	1	0	0	1	0	1	0	1	0	0	0	1	0	1	1	0	1	0	0	1	1	1	0	0	0	0	0	0	1	0	0	1	0	1
B-43 2006		1	0	0	1	0	1	1	0	0	1	0	1	0	1	0	0	0	1	0	1	1	0	1	0	0	1	1	1	0	0	0	0	0	0	1	0	0	1	0	1
B-44 2006		1	0	0	1	0	1	1	0	0	1	0	1	0	1	0	0	0	1	0	1	1	0	1	0	0	1	1	1	0	0	0	0	0	0	1	0	0	1	0	1
B-45 2006		1	0	0	1	0	1	1	0	0	1	0	1	0	1	0	0	0	1	0	1	1	0	1	0	0	1	1	1	0	0	0	0	0	0	1	0	0	1	0	1
B-46 2006		1	0	0	1	0	1	1	0	0	1	0	1	0	1	0	0	0	1	0	1	1	0	1	0	0	1	1	1	0	0	0	0	0	0	1	0	0	1	0	1
B-47 2006		1	0	0	1	0	1	1	0	0	1	0	1	0	1	0	0	0	1	0	1	1	0	1	0	0	1	1	1	0	0	0	0	0	0	1	0	0	1	0	1
B-48 2006		1	0	0	1	0	1	1	0	0	1	0	1	0	1	0	0	0	1	0	1	1	0	1	0	0	1	1	1	0	0	0	0	0	0	1	0	0	1	0	1
B-49 2006		1	0	0	1	0	1	1	0	0	1	0	1	0	1	0	0	0	1	0	1	1	0	1	0	0	1	1	1	0	0	0	0	0	0	1	0	0	1	0	1
B-50 2006		1	0	0	1	0	1	1	0	0	1	0	1	0	1	0	0	0	1	0	1	1	0	1	0	0	1	1	1	0	0	0	0	0	0	1	0	0	1	0	1
B-51 2006		1	0	0	1	0	1	1	0	0	1	0	1	0	1	0	0	0	1	0	1	1	0	1	0	0	1	1	1	0	0	0	0	0	0	1	0	0	1	0	1
B-52 2006		1	0	0	1	0	1	1	0	0	1	0	1	0	1	0	0	0	1	0	1	1	0	1	0	0	1	1	1	0	0	0	0	0	0	1	0	0	1	0	1
B-53 2006		1	0	0	1	0	1	1	0	0	1	0	1	0	1	0	0	0	1	0	1	1	0	1	0	0	1	1	1	0	0	0	0	0	0	1	0	0	1	0	1
B-54 2006		1	0	0	1	0	1	1	0	0	1	0	1	0	1	0	0	0	1	0	1	1	0	1	0	0	1	1	1	0	0	0	0	0	0	1	0	0	1	0	1

#1 Colony BFL	A1030 $(AC)_n(CT)_n$					C625 $(TCA)_n$				B430 $(TC)_n$											A128 $(AC)_n$							A1151 $(CA)_n$										B319 $(AG)_n$		
UGM050509-01	210	212	214	216	218	168	171	174	177	145	147	149	151	153	155	157	159	161	163	165	202	204	206	208	210	213	215	152	154	155	157	159	161	163	165	167	169	195	197	199
BFL1 #1-18 2004	1	0	1	0	0	1	1	0	1	0	1	0	1	0	1	0	1	0	0	0	1	1	0	1	1	0	1	1	0	0	0	0	1	0	1	1	0	0	0	1
BFL1 #1-19 2004	1	0	1	0	0	1	1	0	1	0	1	0	1	0	1	0	1	0	0	0	1	1	0	1	1	0	1	1	0	0	0	0	1	0	1	1	0	0	0	1
BFL1 #1-20 2004	1	0	1	0	0	1	1	0	1	fail	fail	fail	fail	fail	fail	fail	fail	fail	fail	fail	fail	fail	fail	fail	fail	fail	fail	1	0	0	0	0	1	0	1	1	0	0	0	1
BFL1 #1-90 2005	1	0	1	0	0	1	1	0	1	0	1	0	1	0	1	0	1	0	0	0	1	1	0	1	1	0	1	1	0	0	0	0	1	0	1	1	0	0	0	1
BFL1 #1-91 2005	1	0	1	0	0	1	1	0	1	0	1	0	1	0	1	0	1	0	0	0	1	1	0	1	1	0	1	1	0	0	0	0	1	0	1	1	0	0	0	1
BFL1 #1-92 2005	1	0	1	0	0	1	1	0	1	0	1	0	1	0	1	0	1	0	0	0	1	1	0	1	1	0	1	1	0	0	0	0	1	0	1	1	0	0	0	1
BFL1 #1-3 2006	1	0	1	0	0	1	1	0	1	0	1	0	1	0	1	0	1	0	0	0	1	1	0	1	1	0	1	1	0	0	0	0	1	0	1	1	0	0	0	1
BFL1 #1-4 2006	1	0	1	0	0	1	1	0	1	0	1	0	1	0	1	0	1	0	0	0	1	1	0	1	1	0	1	1	0	0	0	0	1	0	1	1	0	0	0	1
BFL1 #1-5 2006	1	0	1	0	0	1	1	0	1	0	1	0	1	0	1	0	1	0	0	0	1	1	0	1	1	0	1	1	0	0	0	0	1	0	1	1	0	0	0	1
BFL1 #1-6 2006	1	0	1	0	0	1	1	0	1	fail	fail	fail	fail	fail	fail	fail	fail	fail	fail	fail	1	1	0	1	1	0	1	1	0	0	0	0	1	0	1	1	0	0	0	1
BFL1 #1-7 2006	1	0	1	0	0	1	1	0	1	0	1	0	1	0	1	0	1	0	0	0	1	1	0	1	1	0	1	1	0	0	0	0	1	0	1	1	0	0	0	1
BFL1 #1-8 2006	1	0	1	0	0	1	1	0	1	0	1	0	1	0	1	0	1	0	0	0	1	1	0	1	1	0	1	1	0	0	0	0	1	0	1	1	0	0	0	1
BFL1 #1-9 2006	1	0	1	0	0	1	1	0	1	0	1	0	1	0	1	0	1	0	0	0	1	1	0	1	1	0	1	1	0	0	0	0	1	0	1	1	0	0	0	1
BFL1 #1-10 2006	1	0	1	0	0	1	1	0	1	0	1	0	1	0	1	0	1	0	0	0	1	1	0	1	1	0	1	1	0	0	0	0	1	0	1	1	0	0	0	1
BFL1 #1-11 2006	1	0	1	0	0	1	1	0	1	0	1	0	1	0	1	0	1	0	0	0	1	1	0	1	1	0	1	1	0	0	0	0	1	0	1	1	0	0	0	1
BFL1 #1-12 2006	1	0	1	0	0	1	1	0	1	0	1	0	1	0	1	0	1	0	0	0	1	1	0	1	1	0	1	1	0	0	0	0	1	0	1	1	0	0	0	1
BFL1 #1-13 2006	1	0	1	0	0	1	1	0	1	0	1	0	1	0	1	0	1	0	0	0	1	1	0	1	1	0	1	1	0	0	0	0	1	0	1	1	0	0	0	1
BFL1 #1-14 2006	1	0	1	0	0	1	1	0	1	0	1	0	1	0	1	0	1	0	0	0	1	1	0	1	1	0	1	1	0	0	0	0	1	0	1	1	0	0	0	1
BFL1 #1-15 2006	1	0	1	0	0	1	1	0	1	0	1	0	1	0	1	0	1	0	0	0	1	1	0	1	1	0	1	1	0	0	0	0	1	0	1	1	0	0	0	1
BFL1 #1-16 2006	1	0	1	0	0	1	1	0	1	0	1	0	1	0	1	0	1	0	0	0	1	1	0	1	1	0	1	1	0	0	0	0	1	0	1	1	0	0	0	1
BFL1 #1-17 2006	1	0	1	0	0	1	1	0	1	0	1	0	1	0	1	0	1	0	0	0	1	1	0	1	1	0	1	1	0	0	0	0	1	0	1	1	0	0	0	1
BFL1 #1-18 2006	1	0	1	0	0	1	1	0	1	0	1	0	1	0	1	0	1	0	0	0	1	1	0	1	1	0	1	1	0	0	0	0	1	0	1	1	0	0	0	1
BFL1 #1-19 2006	1	0	1	0	0	1	1	0	1	0	1	0	1	0	1	0	1	0	0	0	1	1	0	1	1	0	1	1	0	0	0	0	1	0	1	1	0	0	0	1
BFL1 #1-20 2006	1	0	1	0	0	1	1	0	1	0	1	0	1	0	1	0	1	0	0	0	1	1	0	1	1	0	1	1	0	0	0	0	1	0	1	1	0	0	0	1
BFL1 #1-21 2006	1	0	1	0	0	1	1	0	1	fail	fail	fail	fail	fail	fail	fail	fail	fail	fail	fail	1	1	0	1	1	0	1	1	0	0	0	0	1	0	1	1	0	fail	fail	fail
BFL1 #1-22 2006	1	0	1	0	0	1	1	0	1	0	1	0	1	0	1	0	1	0	0	0	1	1	0	1	1	0	1	1	0	0	0	0	1	0	1	1	0	0	0	1
BFL1 #1-23 2006	1	0	1	0	0	1	1	0	1	0	1	0	1	0	1	0	1	0	0	0	1	1	0	1	1	0	1	1	0	0	0	0	1	0	1	1	0	0	0	1
BFL1 #1-24 2006	1	0	1	0	0	1	1	0	1	0	1	0	1	0	1	0	1	0	0	0	1	1	0	1	1	0	1	1	0	0	0	0	1	0	1	1	0	0	0	1
BFL1 #1-25 2006	1	0	1	0	0	1	1	0	1	fail	fail	fail	fail	fail	fail	fail	fail	fail	fail	fail	1	1	0	1	1	0	1	1	0	0	0	0	1	0	1	1	0	0	0	1
BFL1 #1-26 2006	1	0	1	0	0	1	1	0	1	0	1	0	1	0	1	0	1	0	0	0	1	1	0	1	1	0	1	1	0	0	0	0	1	0	1	1	0	0	0	1
BFL1 #1-27 2006	1	0	1	0	0	1	1	0	1	0	1	0	1	0	1	0	1	0	0	0	1	1	0	1	1	0	1	1	0	0	0	0	1	0	1	1	0	0	0	1
BFL1 #1-28 2006	1	0	1	0	0	1	1	0	1	0	1	0	1	0	1	0	1	0	0	0	1	1	0	1	1	0	1	1	0	0	0	0	1	0	1	1	0	0	0	1
BFL1 #1-29 2006	1	0	1	0	0	1	1	0	1	0	1	0	1	0	1	0	1	0	0	0	1	1	0	1	1	0	1	1	0	0	0	0	1	0	1	1	0	0	0	1
BFL1 #1-30 2006	1	0	1	0	0	1	1	0	1	0	1	0	1	0	1	0	1	0	0	0	1	1	0	1	1	0	1	1	0	0	0	0	1	0	1	1	0	0	0	1
BFL1 #1-31 2006	1	0	1	0	0	1	1	0	1	0	1	0	1	0	1	0	1	0	0	0	fail	fail	fail	fail	fail	fail	fail	1	0	0	0	0	1	0	1	1	0	0	0	1
BFL1 #1-32 2006	1	0	1	0	0	1	1	0	1	0	1	0	1	0	1	0	1	0	0	0	1	1	0	1	1	0	1	1	0	0	0	0	1	0	1	1	0	0	0	1
BFL1 #1-33 2006	fail	fail	fail	fail	fail	fail	fail	fail	fail	0	1	0	1	0	1	0	1	0	0	0	1	1	0	1	1	0	1	1	0	0	0	0	1	0	1	1	0	0	0	1
BFL1 #1-34 2006	1	0	1	0	0	1	1	0	1	0	1	0	1	0	1	0	1	0	0	0	1	1	0	1	1	0	1	1	0	0	0	0	1	0	1	1	0	0	0	1
BFL1 #1-35 2006	1	0	1	0	0	1	1	0	1	0	1	0	1	0	1	0	1	0	0	0	1	1	0	1	1	0	1	1	0	0	0	0	1	0	1	1	0	0	0	1
BFL1 #1-36 2006	1	0	1	0	0	1	1	0	1	0	1	0	1	0	1	0	1	0	0	0	1	1	0	1	1	0	1	1	0	0	0	0	1	0	1	1	0	0	0	1
BFL1 #1-37 2006	1	0	1	0	0	1	1	0	1	0	1	0	1	0	1	0	1	0	0	0	1	1	0	1	1	0	1	1	0	0	0	0	1	0	1	1	0	0	0	1
BFL1 #1-38 2006	1	0	1	0	0	1	1	0	1	0	1	0	1	0	1	0	1	0	0	0	1	1	0	1	1	0	1	1	0	0	0	0	1	0	1	1	0	0	0	1
BFL1 #1-39 2006	1	0	1	0	0	1	1	0	1	0	1	0	1	0	1	0	1	0	0	0	1	1	0	1	1	0	1	1	0	0	0	0	1	0	1	1	0	0	0	1
BFL1 #1-40 2006	1	0	1	0	0	1	1	0	1	0	1	0	1	0	1	0	1	0	0	0	1	1	0	1	1	0	1	1	0	0	0	0	1	0	1	1	0	0	0	1
BFL1 #1-42 2006	1	0	1	0	0	1	1	0	1	0	1	0	1	0	1	0	1	0	0	0	1	1	0	1	1	0	1	1	0	0	0	0	1	0	1	1	0	0	0	1
BFL1 #1-43 2006	1	0	1	0	0	1	1	0	1	0	1	0	1	0	1	0	1	0	0	0	1	1	0	1	1	0	1	1	0	0	0	0	1	0	1	1	0	0	0	1
BFL1 #1-44 2006	1	0	1	0	0	1	1	0	1	0	1	0	1	0	1	0	1	0	0	0	1	1	0	1	1	0	1	1	0	0	0	0	1	0	1	1	0	0	0	1
BFL1 #1-45 2006	1	0	1	0	0	1	1	0	1	0	1	0	1	0	1	0	1	0	0	0	1	1	0	1	1	0	1	1	0	0	0	0	1	0	1	1	0	0	0	1
BFL1 #1-46 2006	1	0	1	0	0	1	1	0	1	0	1	0	1	0	1	0	1	0	0	0	1	1	0	1	1	0	1	1	0	0	0	0	1	0	1	1	0	0	0	1
BFL1 #1-47 2006	1	0	1	0	0	1	1	0	1	0	1	0	1	0	1	0	1	0	0	0	1	0	0	1	1	0	1	1	0	0	0	0	1	0	1	1	0	0	0	1
BFL1 #1a 2007	1	0	1	0	0	1	1	0	1	0	1	0	1	0	1	0	1	0	0	0	1	1	0	1	1	0	1	1	0	0	0	0	1	0	1	1	0	0	0	1
BFL1 #1b 2007	1	0	1	0	0	1	1	0	1	0	1	0	1	0	1	0	1	0	0	0	1	1	0	1	1	0	1	1	0	0	0	0	1	0	1	1	0	0	0	1

BFL1 #1c 2007	1	0	1	0	0	1	1	0	1	0	1	0	1	0	1	0	1	0	0	0	1	1	0	1	1	0	1	1	0	0	0	0	1	0	1	1	0	0	0	1
BFL1 #1a 2008	1	0	1	0	0	1	1	0	1	0	1	0	1	0	1	0	1	0	0	0	1	1	0	1	1	0	1	1	0	0	0	0	1	0	1	1	0	0	0	1
BFL1 #1b 2008	1	0	1	0	0	1	1	0	1	0	1	0	1	0	1	0	1	0	0	0	1	1	0	1	1	0	1	1	0	0	0	0	1	0	1	1	0	0	0	1
BFL1 #1c 2008	1	0	1	0	0	1	1	0	1	0	1	0	1	0	1	0	1	0	0	0	1	1	0	1	1	0	1	1	0	0	0	0	1	0	1	1	0	0	0	1
BFL1 #1a 2009	1	0	1	0	0	1	1	0	1	0	1	0	1	0	1	0	1	0	0	0	1	1	0	1	1	0	1	1	0	0	0	0	1	0	1	1	0	0	0	1
BFL1 #1b 2009	1	0	1	0	0	1	1	0	1	0	1	0	1	0	1	0	1	0	0	0	1	1	0	1	1	0	1	1	0	0	0	0	1	0	1	1	0	0	0	1
BFL1 #1c 2009	1	0	1	0	0	1	1	0	1	0	1	0	1	0	1	0	1	0	0	0	1	1	0	1	1	0	1	1	0	0	0	0	1	0	1	1	0	0	0	1
BFL1 #1a 2010	1	0	1	0	0	1	1	0	1	0	1	0	1	0	1	0	1	0	0	0	1	1	0	1	1	0	1	1	0	0	0	0	1	0	1	1	0	0	0	1
BFL1 #1b 2010	1	0	1	0	0	1	1	0	1	0	1	0	1	0	1	0	1	0	0	0	1	1	0	1	1	0	1	1	0	0	0	0	1	0	1	1	0	0	0	1
BFL1 #1c 2010	1	0	1	0	0	1	1	0	1	0	1	0	1	0	1	0	1	0	0	0	1	1	0	1	1	0	1	1	0	0	0	0	1	0	1	1	0	0	0	1

#2 Colony BFL	A1030 $(AC)_n(CT)_n$					C625 $(TCA)_n$				B430 $(TC)_n$											A128 $(AC)_n$							A1151 $(CA)_n$										B319 $(AG)_n$		
UGM050509-02	210	212	214	216	218	168	171	174	177	145	147	149	151	153	155	157	159	161	163	165	202	204	206	208	210	213	215	152	154	155	157	159	161	163	165	167	169	195	197	199
BFL2 #2-81 2004	0	0	1	0	0	1	0	0	0	0	0	0	1	0	0	1	0	0	0	1	0	1	1	0	0	0	0	0	0	0	0	0	0	1	0	1	0	1	0	0
BFL2 #2-82 2004	0	0	1	0	0	1	0	0	0	0	0	0	1	0	0	1	0	0	0	1	0	1	1	0	0	0	0	0	0	0	0	0	0	1	0	1	0	1	0	0
BFL2 #2-84 2004	0	0	1	0	0	1	0	0	0	0	0	0	1	0	0	1	0	0	0	1	0	1	1	0	0	0	0	0	0	0	0	0	0	1	0	1	0	1	0	0
BFL2 #2a 2007	0	0	1	0	0	1	0	0	0	0	0	0	1	0	0	1	0	0	0	1	0	1	1	0	0	0	0	0	0	0	0	0	0	1	0	1	0	1	0	0
BFL2 #2b 2007	0	0	1	0	0	1	0	0	0	0	0	0	1	0	0	1	0	0	0	1	0	1	1	0	0	0	0	0	0	0	0	0	0	1	0	1	0	1	0	0
BFL2 #2c 2007	0	0	1	0	0	1	0	0	0	0	0	0	1	0	0	1	0	0	0	1	0	1	1	0	0	0	0	0	0	0	0	0	0	1	0	1	0	1	0	0
BFL2 #2a 2008	0	0	1	0	0	1	0	0	0	0	0	0	1	0	0	1	0	0	0	1	0	1	1	0	0	0	0	0	0	0	0	0	0	1	0	1	0	1	0	0
BFL2 #2b 2008	0	0	1	0	0	1	0	0	0	0	0	0	1	0	0	1	0	0	0	1	0	1	1	0	0	0	0	0	0	0	0	0	0	1	0	1	0	1	0	0
BFL2 #2c 2008	0	0	1	0	0	1	0	0	0	0	0	0	1	0	0	1	0	0	0	1	0	1	1	0	0	0	0	0	0	0	0	0	0	1	0	1	0	1	0	0
BFL2 #2a 2009	0	0	1	0	0	1	0	0	0	0	0	0	1	0	0	1	0	0	0	1	0	1	1	0	0	0	0	0	0	0	0	0	0	1	0	1	0	1	0	0
BFL2 #2b 2009	0	0	1	0	0	1	0	0	0	0	0	0	1	0	0	1	0	0	0	1	0	1	1	0	0	0	0	0	0	0	0	0	0	1	0	1	0	1	0	0
BFL2 #2c 2009	0	0	1	0	0	1	0	0	0	0	0	0	1	0	0	1	0	0	0	1	0	1	1	0	0	0	0	0	0	0	0	0	0	1	0	1	0	1	0	0
BFL2 #2a 2010	0	0	1	0	0	1	0	0	0	0	0	0	1	0	0	1	0	0	0	1	0	1	1	0	0	0	0	0	0	0	0	0	0	1	0	1	0	1	0	0
BFL2 #2b 2010	0	0	1	0	0	1	0	0	0	0	0	0	1	0	0	1	0	0	0	1	0	1	1	0	0	0	0	0	0	0	0	0	0	1	0	1	0	1	0	0
BFL2 #2c 2010	0	0	1	0	0	1	0	0	0	0	0	0	1	0	0	1	0	0	0	1	0	1	1	0	0	0	0	0	0	0	0	0	0	1	0	1	0	1	0	0

#7 Colony BFL	A1030 $(AC)_n(CT)_n$					C625 $(TCA)_n$				B430 $(TC)_n$											A128 $(AC)_n$							A1151 $(CA)_n$										B319 $(AG)_n$		
UGM050509-07	210	212	214	216	218	168	171	174	177	145	147	149	151	153	155	157	159	161	163	165	202	204	206	208	210	213	215	152	154	155	157	159	161	163	165	167	169	195	197	199
BFL7 #2a 2004	1	0	1	0	0	1	1	0	1	0	1	0	1	0	1	0	1	0	0	0	1	1	0	1	1	0	1	1	0	0	0	0	1	0	1	1	0	0	0	1
BFL7 #2b 2004	1	0	1	0	0	1	1	0	1	0	1	0	1	0	1	0	1	0	0	0	1	1	0	1	1	0	1	1	0	0	0	0	1	0	1	1	0	0	0	1
BFL7 #2a 2009	1	0	1	0	0	1	1	0	1	0	1	0	1	0	1	0	1	0	0	0	1	1	0	1	1	0	1	1	0	0	0	0	1	0	1	1	0	0	0	1
BFL7 #2b 2009	1	0	1	0	0	1	1	0	1	0	1	0	1	0	1	0	1	0	0	0	1	1	0	1	1	0	1	1	0	0	0	0	1	0	1	1	0	0	0	1
BFL7 #2c 2009	1	0	1	0	0	1	1	0	1	0	1	0	1	0	1	0	1	0	0	0	1	1	0	1	1	0	1	1	0	0	0	0	1	0	1	1	0	0	0	1
BFL7 #2a 2010	1	0	1	0	0	1	1	0	1	fail	fail	fail	fail	fail	fail	fail	fail	fail	fail	fail	fail	fail	fail	fail	fail	fail	fail	fail	fail	fail	fail	fail	fail	fail	fail	fail	fail	0	0	1

A-Colony Hornsby	A1030 $(AC)_n(CT)_n$					C625 $(TCA)_n$				B430 $(TC)_n$											A128 $(AC)_n$							A1151 $(CA)_n$										B319 $(AG)_n$		
UGM060121-1	210	212	214	216	218	168	171	174	177	145	147	149	151	153	155	157	159	161	163	165	202	204	206	208	210	213	215	152	154	155	157	159	161	163	165	167	169	195	197	199
A-1 2006	0	0	0	1	0	1	0	0	0	0	0	0	1	0	0	1	0	0	0	1	0	1	1	0	0	0	0	0	0	0	0	0	0	1	0	1	0	1	0	0
A-2 2006	0	0	0	1	0	1	0	0	0	0	0	0	1	0	0	1	0	0	0	1	0	1	1	0	0	0	0	0	0	0	0	0	0	1	0	1	0	1	0	0
A-3 2006	0	0	0	1	0	1	0	0	0	fail	fail	fail	fail	fail	fail	fail	fail	fail	fail	fail	fail	fail	fail	fail	fail	fail	fail	0	0	0	0	0	0	1	0	1	0	1	0	0
A-20 2006	0	0	0	1	0	1	0	0	0	0	0	0	1	0	0	1	0	0	0	1	0	1	1	0	0	0	0	fail	fail	fail	fail	fail	fail	fail	fail	fail	fail	fail	fail	fail
A-21 2006	0	0	0	1	0	1	0	0	0	0	0	0	1	0	0	1	0	0	0	1	0	1	1	0	0	0	0	0	0	0	0	0	0	1	0	1	0	1	0	0
A-22 2006	0	0	0	1	0	1	0	0	0	0	0	0	1	0	0	1	0	0	0	1	0	1	1	0	0	0	0	0	0	0	0	0	0	1	0	1	0	1	0	0
A-23 2006	0	0	0	1	0	1	0	0	0	0	0	0	1	0	0	1	0	0	0	1	0	1	1	0	0	0	0	0	0	0	0	0	0	1	0	1	0	1	0	0
A-24 2006	0	0	0	1	0	1	0	0	0	0	0	0	1	0	0	1	0	0	0	1	0	1	1	0	0	0	0	0	0	0	0	0	0	1	0	1	0	1	0	0
A-25 2006	0	0	0	1	0	1	0	0	0	0	0	0	1	0	0	1	0	0	0	1	0	1	1	0	0	0	0	0	0	0	0	0	0	1	0	1	0	1	0	0
A-26 2006	0	0	0	1	0	1	0	0	0	0	0	0	1	0	0	1	0	0	0	1	0	1	1	0	0	0	0	0	0	0	0	0	0	1	0	1	0	1	0	0
A-27 2006	0	0	0	1	0	1	0	0	0	0	0	0	1	0	0	1	0	0	0	1	0	1	1	0	0	0	0	0	0	0	0	0	0	1	0	1	0	1	0	0
A-28 2006	0	0	0	1	0	1	0	0	0	0	0	0	1	0	0	1	0	0	0	1	0	1	1	0	0	0	0	0	0	0	0	0	0	1	0	1	0	1	0	0
A-29 2006	0	0	0	1	0	1	0	0	0	0	0	0	1	0	0	1	0	0	0	1	0	1	1	0	0	0	0	0	0	0	0	0	0	1	0	1	0	1	0	0
A-30 2006	0	0	0	1	0	1	0	0	0	0	0	0	1	0	0	1	0	0	0	1	0	1	1	0	0	0	0	0	0	0	0	0	0	1	0	1	0	1	0	0
A-31 2006	0	0	0	1	0	1	0	0	0	0	0	0	1	0	0	1	0	0	0	1	0	1	1	0	0	0	0	0	0	0	0	0	0	1	0	1	0	1	0	0
A-32 2006	0	0	0	1	0	1	0	0	0	0	0	0	1	0	0	1	0	0	0	1	0	1	1	0	0	0	0	0	0	0	0	0	0	1	0	1	0	1	0	0
A-33 2006	0	0	0	1	0	1	0	0	0	0	0	0	1	0	0	1	0	0	0	1	0	1	1	0	0	0	0	0	0	0	0	0	0	1	0	1	0	1	0	0
A-34 2006	0	0	0	1	0	1	0	0	0	0	0	0	1	0	0	1	0	0	0	1	0	1	1	0	0	0	0	0	0	0	0	0	0	1	0	1	0	1	0	0
A-35 2006	0	0	0	1	0	1	0	0	0	0	0	0	1	0	0	1	0	0	0	1	0	1	1	0	0	0	0	0	0	0	0	0	0	1	0	1	0	1	0	0
A-37 2006	0	0	0	1	0	1	0	0	0	0	0	0	1	0	0	1	0	0	0	1	0	1	1	0	0	0	0	0	0	0	0	0	0	1	0	1	0	1	0	0

A-38 2006	0	0	0	1	0	1	0	0	0	0	0	0	1	0	0	1	0	0	0	1	0	1	1	0	0	0	0	0	0	0	0	0	0	1	0	1	0	1	0	0
A-39 2006	0	0	0	1	0	1	0	0	0	0	0	0	1	0	0	1	0	0	0	1	0	1	1	0	0	0	0	0	0	0	0	0	0	1	0	1	0	1	0	0
A-40 2006	0	0	0	1	0	1	0	0	0	0	0	0	1	0	0	1	0	0	0	1	0	1	1	0	0	0	0	0	0	0	0	0	0	1	0	1	0	1	0	0
A-41 2006	0	0	0	1	0	1	0	0	0	0	0	0	1	0	0	1	0	0	0	1	0	1	1	0	0	0	0	0	0	0	0	0	0	1	0	1	0	1	0	0
A-42 2006	0	0	0	1	0	1	0	0	0	0	0	0	1	0	0	1	0	0	0	1	0	1	1	0	0	0	0	0	0	0	0	0	0	1	0	1	0	1	0	0
A-43 2006	0	0	0	1	0	1	0	0	0	0	0	0	1	0	0	1	0	0	0	1	0	1	1	0	0	0	0	0	0	0	0	0	0	1	0	1	0	1	0	0
A-44 2006	0	0	0	1	0	1	0	0	0	0	0	0	1	0	0	1	0	0	0	1	0	1	1	0	0	0	0	0	0	0	0	0	0	1	0	1	0	1	0	0
A-45 2006	0	0	0	1	0	1	0	0	0	0	0	0	1	0	0	1	0	0	0	1	0	1	1	0	0	0	0	0	0	0	0	0	0	1	0	1	0	1	0	0
A-46 2006	0	0	0	1	0	1	0	0	0	0	0	0	1	0	0	1	0	0	0	1	0	1	1	0	0	0	0	0	0	0	0	0	0	1	0	1	0	1	0	0
A-47 2006	0	0	0	1	0	1	0	0	0	0	0	0	1	0	0	1	0	0	0	1	0	1	1	0	0	0	0	0	0	0	0	0	0	1	0	1	0	1	0	0
A-48 2006	0	0	0	1	0	1	0	0	0	0	0	0	1	0	0	1	0	0	0	1	0	1	1	0	0	0	0	0	0	0	0	0	0	1	0	1	0	1	0	0
A-49 2006	0	0	0	1*	0	1	0	0	0	0	0	0	1	0	0	1	0	0	0	1	0	1	1	0	0	0	0	0	0	0	0	0	0	1	0	1	0	1	0	0
A-50 2006	0	0	0	1	0	1	0	0	0	0	0	0	1	0	0	1	0	0	0	1	0	1	1	0	0	0	0	0	0	0	0	0	0	1	0	1	0	1	0	0
A-51 2006	0	0	0	1	0	1	0	0	0	0	0	0	1	0	0	1	0	0	0	1	0	1	1	0	0	0	0	0	0	0	0	0	0	1	0	1	0	1	0	0
A-52 2006	0	0	0	1	0	1	0	0	0	0	0	0	1	0	0	1	0	0	0	1	0	1	1	0	0	0	0	0	0	0	0	0	0	1	0	1	0	1	0	0
A-53 2006	0	0	0	1	0	1	0	0	0	0	0	0	1	0	0	1	0	0	0	1	0	1	1	0	0	0	0	0	0	0	0	0	0	1	0	1	0	1	0	0
A-54 2006	0	0	0	1	0	1	0	0	0	0	0	0	1	0	0	1	0	0	0	1	0	1	1	0	0	0	0	0	0	0	0	0	0	1	0	1	0	1	0	0
A-55 2006	0	0	0	1	0	1	0	0	0	0	0	0	1	0	0	1	0	0	0	1	0	1	1	0	0	0	0	0	0	0	0	0	0	1	0	1	0	1	0	0
A-56 2006	0	0	0	1	0	1	0	0	0	0	0	0	1	0	0	1	0	0	0	1	0	1	1	0	0	0	0	0	0	0	0	0	0	1	0	1	0	1	0	0
A-57 2006	0	0	0	1	0	1	0	0	0	0	0	0	1	0	0	1	0	0	0	1	0	1	1	0	0	0	0	0	0	0	0	0	0	1	0	1	0	1	0	0
A-58 2006	0	0	0	1	0	1	0	0	0	0	0	0	1	0	0	1	0	0	0	1	0	1	1	0	0	0	0	0	0	0	0	0	0	1	0	1	0	1	0	0
A-59 2006	0	0	0	1	0	1	0	0	0	0	0	0	1	0	0	1	0	0	0	1	0	1	1	0	0	0	0	0	0	0	0	0	0	1	0	1	0	1	0	0
A-60 2006	0	0	0	1	0	1	0	0	0	0	0	0	1	0	0	1	0	0	0	1	0	1	1	0	0	0	0	0	0	0	0	0	0	1	0	1	0	1	0	0
A-61 2006	0	0	0	1	0	1	0	0	0	0	0	0	1	0	0	1	0	0	0	1	0	1	1	0	0	0	0	0	0	0	0	0	0	1	0	1	0	1	0	0
A-62 2006	0	0	0	1	0	1	0	0	0	0	0	0	1	0	0	1	0	0	0	1	0	1	1	0	0	0	0	0	0	0	0	0	0	1	0	1	0	1	0	0
A-63 2006	0	0	0	1	0	1	0	0	0	0	0	0	1	0	0	1	0	0	0	1	0	1	1	0	0	0	0	0	0	0	0	0	0	1	0	1	0	1	0	0
A-64 2006	0	0	0	1	0	1	0	0	0	0	0	0	1	0	0	1	0	0	0	1	0	1	1	0	0	0	0	0	0	0	0	0	0	1	0	1	0	1	0	0
A-65 2006	0	0	0	1	0	1	0	0	0	0	0	0	1	0	0	1	0	0	0	1	0	1	1	0	0	0	0	0	0	0	0	0	0	1	0	1	0	1	0	0

**B-Colony Hornsby**	**A1030 (AC)$_n$(CT)$_n$**					**C625 (TCA)$_n$**				**B430 (TC)$_n$**											**A128 (AC)$_n$**							**A1151 (CA)$_n$**										**B319 (AG)$_n$**		
**UGM060121-02**	**210**	**212**	**214**	**216**	**218**	**168**	**171**	**174**	**177**	**145**	**147**	**149**	**151**	**153**	**155**	**157**	**159**	**161**	**163**	**165**	**202**	**204**	**206**	**208**	**210**	**213**	**215**	**152**	**154**	**155**	**157**	**159**	**161**	**163**	**165**	**167**	**169**	**195**	**197**	**199**
B-1 2006	1	1	0	0	1	0	0	1	1	1	1	0	0	0	0	1	0	1	1	0	0	0	1	1	0	0	0	0	0	0	0	0	0	1	1	1	1	0	1	0
B-2 2006	1	1	0	0	1	0	0	1	1	1	1	0	0	0	0	1	0	1	1	0	0	0	1	1	0	0	0	0	0	0	0	0	0	1	1	1	1	0	1	0
B-3 2006	1	1	0	0	1	0	0	1	1	1	1	0	0	0	0	1	0	1	1	0	0	0	1	1	0	0	0	0	0	0	0	0	0	1	1	1	1	0	1	0
B-4 2006	1	1	0	0	1	0	0	1	1	1	1	0	0	0	0	1	0	1	1	0	fail	fail	fail	fail	fail	fail	fail	0	0	0	0	0	0	1	1	1	1	0	1	0
B-5 2006	1	1	0	0	1	0	0	1	1	1	1	0	0	0	0	1	0	1	1	0	0	0	1	1	0	0	0	0	0	0	0	0	0	1	1	1	1	0	1	0
B-6 2006	1	1	0	0	1	0	0	1	1	1	1	0	0	0	0	1	0	1	1	0	0	0	1	1	0	0	0	0	0	0	0	0	0	1	1	1	1	0	1	0
B-7 2006	1	1	0	0	1	0	0	1	1	1	1	0	0	0	0	1	0	1	1	0	0	0	1	1	0	0	0	0	0	0	0	0	0	1	1	1	1	0	1	0
B-8 2006	1	1	0	0	1	0	0	1	1	1	1	0	0	0	0	1	0	1	1	0	0	0	1	1	0	0	0	0	0	0	0	0	0	1	1	1	1	0	1	0
B-9 2006	1	1	0	0	1	0	0	1	1	1	1	0	0	0	0	1	0	1	1	0	0	0	1	1	0	0	0	0	0	0	0	0	0	1	1	1	1	0	1	0
B-10 2006	1	1	0	0	1	0	0	1	1	1	1	0	0	0	0	1	0	1	1	0	0	0	1	1	0	0	0	0	0	0	0	0	0	1	1	1	1	0	1	0
B-11 2006	fail	fail	fail	fail	fail	fail	fail	fail	fail	1	**0**	0	0	0	0	**0**	0	**0**	**0**	0	0	0	1	1	0	0	0	0	0	0	0	0	0	1	1	**0**	**0**	fail	fail	fail
B-12 2006	1	1	0	0	1	0	0	1	1	1	1	0	0	0	0	1	0	1	1	0	0	0	1	1	0	0	0	0	0	0	0	0	0	1	1	1	1	0	1	0
B-13 2006	1	1	0	0	1	0	0	1	1	1	1	0	0	0	0	1	0	1	1	0	0	0	1	1	0	0	0	0	0	0	0	0	0	1	1	1	1	0	1	0
B-14 2006	1	1	0	0	1	0	0	1	1	1	1	0	0	0	0	1	0	1	1	0	0	0	1	1	0	0	0	0	0	0	0	0	0	1	1	1	1	0	1	0
B-15 2006	1	1	0	0	1	0	0	1	1	1	1	0	0	0	0	1	0	1	1	0	0	0	1	1	0	0	0	0	0	0	0	0	0	1	1	1	1	0	1	0
B-20 2006	1	1	0	0	1	0	0	1	1	1	1	0	0	0	0	1	0	1	1	0	0	0	1	1	0	0	0	0	0	0	0	0	0	1	1	1	1	0	1	0
B-21 2006	1	1	0	0	1	0	0	1	1	1	1	0	0	0	0	1	0	1	1	0	0	0	1	1	0	0	0	0	0	0	0	0	0	1	1	1	1	0	1	0
B-22 2006	1	1	0	0	1	0	0	1	1	1	1	0	0	0	0	1	0	1	1	0	0	0	1	1	0	0	0	0	0	0	0	0	0	1	1	1	1	0	1	0
B-23 2006	1	1	0	0	**0**	fail	fail	fail	fail	**0**	1	0	0	0	0	**0**	0	1	**0**	0	0	0	1	**0**	0	0	0	0	0	0	0	0	0	1	1	**0**	1	fail	fail	fail
B-24 2006	1	1	0	0	1	0	0	1	1	1	1	0	0	0	0	1	0	1	1	0	0	0	1	1	0	0	0	0	0	0	0	0	0	1	1	1	1	0	1	0
B-25 2006	1	1	0	0	1	0	0	1	1	1	1	0	0	0	0	1	0	1	1	0	0	0	1	1	0	0	0	0	0	0	0	0	0	1	1	1	1	0	1	0
B-26 2006	1	1	0	0	1	0	0	1	1	1	1	0	0	0	0	1	0	1	1	0	0	0	1	1	0	0	0	0	0	0	0	0	0	1	1	1	1	0	1	0
B-27 2006	1	1	0	0	1	0	0	1	1	1	1	0	0	0	0	1	0	1	1	0	0	0	1	1	0	0	0	0	0	0	0	0	0	1	1	1	1	0	1	0
B-28 2006	1	1	0	0	1	0	0	1	1	1	1	0	0	0	0	1	0	1	1	0	0	0	1	1	0	0	0	0	0	0	0	0	0	1	1	1	1	0	1	0
B-29 2006	1	1	0	0	1	0	0	1	1	1	1	0	0	0	0	1	0	1	1	0	0	0	1	1	0	0	0	0	0	0	0	0	0	1	1	1	1	0	1	0
B-30 2006	1	1	0	0	1	0	0	1	1	1	1	0	0	0	0	1	0	1	1	0	0	0	1	1	0	0	0	0	0	0	0	0	0	1	1	1	1	0	1	0
B-31 2006	1	1	0	0	1	0	0	1	1	1	1	0	0	0	0	1	0	1	1	0	0	0	1	1	0	0	0	0	0	0	0	0	0	1	1	1	1	0	1	0
B-32 2006	1	1	0	0	1	0	0	1	1	1	1	0	0	0	0	1	0	1	1	0	0	0	1	1	0	0	0	0	0	0	0	0	0	1	1	1	1	0	1	0
B-33 2006	1	1	0	0	1	0	0	1	1	1	1	0	0	0	0	1	0	1	1	0	0	0	1	1	0	0	0	0	0	0	0	0	0	1	1	1	1	0	1	0
B-34 2006	1	1	0	0	1	0	0	1	1	1	1	0	0	0	0	1	0	1	1	0	0	0	1	1	0	0	0	0	0	0	0	0	0	1	1	1	1	0	1	0
B-35 2006	1	1	0	0	1	0	0	1	1	1	1	0	0	0	0	1	0	1	1	0	0	0	1	1	0	0	0	0	0	0	0	0	0	1	1	1	1	0	1	0
B-36 2006	1	1	0	0	1	0	0	1	1	1	1	0	0	0	0	1	0	1	1	0	0	0	1	1	0	0	0	0	0	0	0	0	0	1	1	1	1	0	1	0
B-37 2006	1	1	0	0	1	0	0	1	1	1	1	0	0	0	0	1	0	1	1	0	0	0	1	1	0	0	0	0	0	0	0	0	0	1	1	1	1	0	1	0

B-38 2006	1	1	0	0	1	0	0	1	1	1	1	0	0	0	0	1	0	1	1	0	0	0	1	1	0	0	0	0	0	0	0	0	0	1	1	1	1	0	1	0
B-39 2006	1	1	0	0	1	0	0	1	1	1	1	0	0	0	0	1	0	1	1	0	0	0	1	1	0	0	0	0	0	0	0	0	0	1	1	1	1	0	1	0
B-40 2006	1	1	0	0	1	0	0	1	1	1	1	0	0	0	0	1	0	1	1	0	0	0	1	1	0	0	0	0	0	0	0	0	0	1	1	1	1	0	1	0
B-41 2006	1	1	0	0	1	0	0	1	1	1	1	0	0	0	0	1	0	1	1	0	0	0	1	1	0	0	0	0	0	0	0	0	0	1	1	1	1	0	1	0
B-42 2006	1	1	0	0	1	0	0	1	1	1	1	0	0	0	0	1	0	1	1	0	0	0	1	1	0	0	0	0	0	0	0	0	0	1	1	1	1	0	1	0
B-43 2006	1	1	0	0	1	0	0	1	1	1	1	0	0	0	0	1	0	1	1	0	0	0	1	1	0	0	0	0	0	0	0	0	0	1	1	1	1	0	1	0
B-44 2006	1	1	0	0	1	0	0	1	1	1	1	0	0	0	0	1	0	1	1	0	0	0	1	1	0	0	0	0	0	0	0	0	0	1	1	1	1	0	1	0
B-45 2006	1	1	0	0	1	0	0	1	1	1	1	0	0	0	0	1	0	1	1	0	0	0	1	1	0	0	0	0	0	0	0	0	0	1	1	1	1	0	1	0
B-46 2006	1	1	0	0	1	0	0	1	1	1	1	0	0	0	0	1	0	1	1	0	0	0	1	1	0	0	0	0	0	0	0	0	0	1	1	1	1	0	1	0
B-47 2006	1	1	0	0	1	0	0	1	1	1	1	0	0	0	0	1	0	1	1	0	0	0	1	1	0	0	0	0	0	0	0	0	0	1	1	1	1	0	1	0
B-48 2006	1	1	0	0	1	0	0	1	1	1	1	0	0	0	0	1	0	1	1	0	0	0	1	1	0	0	0	0	0	0	0	0	0	1	1	1	1	0	1	0
B-49 2006	1	1	0	0	1	0	0	1	1	1	1	0	0	0	0	1	0	1	1	0	0	0	1	1	0	0	0	0	0	0	0	0	0	1	1	1	1	0	1	0
B-50 2006	1	1	0	0	1	0	0	1	1	1	1	0	0	0	0	1	0	1	1	0	0	0	1	1	0	0	0	0	0	0	0	0	0	1	1	1	1	0	1	0
B-51 2006	1	1	0	0	1	0	0	1	1	1	1	0	0	0	0	1	0	1	1	0	0	0	1	1	0	0	0	0	0	0	0	0	0	1	1	1	1	0	1	0
B-52 2006	1	1	0	0	1	0	0	1	1	1	1	0	0	0	0	1	0	1	1	0	0	0	1	1	0	0	0	0	0	0	0	0	0	1	1	1	1	0	1	0
B-53 2006	1	1	0	0	1	0	0	1	1	1	1	0	0	0	0	1	0	1	1	0	0	0	1	1	0	0	0	0	0	0	0	0	0	1	1	1	1	0	1	0
B-54 2006	1	1	0	0	1	0	0	1	1	1	1	0	0	0	0	1	0	1	1	0	0	0	1	1	0	0	0	0	0	0	0	0	0	1	1	1	1	0	1	0
