# Supplementary material for: Monoculture of Leafcutter Ant Gardens
Source: PLoS One. 2010 Sep 10;5(9):e12668. doi: 10.1371/journal.pone.0012668 (PMC2937030; doi:10.1371/journal.pone.0012668)
Supplement: Table S2 — Within-nest Attamyces diversity for Atta texana. (0.07 MB PDF) [file pone.0012668.s003.pdf]

Table S2. Microsatellite marker profiles of *Attamyces* in nests of *Atta texana* from Texas and Louisiana.

Different gardens were accessed for each nest through excavation; in all but the last two nests, gardens were excavated at different times (see Collection Date) and in different sectors of each nest.

For each collection, mycelium from one garden chamber was genotyped at 12 microsatellite DNA markers. Markers were scored blind without knowledge of previous scoring in the repeat genotyping.

The label "stut" indicates that a stutter band is sometimes erroneously scored at this marker size. One such stutter band was accidentally scored in marker 214 of locus A1030 (highlighted in yellow).

Apart from this single accidental scoring of a stutter band, all markers scored were identical for the multiple *Attamyces* collection within each nest.

1 = marker present. 0 = marker absent. The label "fail" indicates that the genotyping analysis failed for the particular locus and was not repeated.

Nest ID	Collection Code	Collection Date	Chamber	A1132 (TCA repeat)									B12 (GA repeat)						C101 (CAG/CAA repeat)							C117 (CAG/CAC repeat)					C126 (CTG)		A1030 (AC/CT repeat)					C625 (TCA repeat)			
				**199**	**202**	**205**	**208**	**211**	**214**	**217**	**220**	**223**	**231**	**232**	**234**	**236**	**238**	**240**	**100**	**103**	**106**	**109**	**112**	**115**	**118**	**239**	**242**	**245**	**248**	**251**	**244**	**247**	**210**	**212**	**214**	**216**	**218**	**168**	**171**	**174**	**177**
				stut					stut														stut					stut							stut			stut			stut
UGM060121-01	UGM060121-01	21. Jan. 2006	Chamber 1	1	1	0	0	0	0	0	1	0	0	0	1	0	1	0	0	0	0	0	1	1	1	0	0	0	1	1	1	0	0	0	0	1	0	1	0	0	0
UGM060121-01	UGM060327-01	27. Mar. 2006	Chamber 2	1	1	0	0	0	0	0	1	0	0	0	1	0	1	0	0	0	0	0	1	1	1	0	0	0	1	1	1	0	0	0	0	1	0	1	0	0	0
UGM060121-01	UGM060622-01	22. Jun. 2006	Chamber 3	1	1	0	0	0	0	0	1	0	fail	fail	fail	fail	fail	fail	0	0	0	0	1	1	1	0	0	0	1	1	1	0	0	0	0	1	0	1	0	0	0
UGM060121-01	AR061007-01	07. Oct. 2006	Chamber 4	1	1	0	0	0	0	0	1	0	0	0	1	0	1	0	0	0	0	0	1	1	1	0	0	0	1	1	1	0	0	0	0	1	0	1	0	0	0
UGM051218-02	UGM051218-02	18. Dec. 2005	Chamber 1	1	1	0	0	0	0	0	1	0	0	0	1	0	1	0	0	0	0	0	1	1	1	0	0	0	1	1	1	0	0	0	0	1	0	1	0	0	0
UGM051218-02	UGM060504-01A	04. May 2006	Chamber 2	1	1	0	0	0	0	0	1	0	0	0	1	0	1	0	0	0	0	0	1	1	1	0	0	0	1	1	1	0	0	0	1	1	0	1	0	0	0
UGM051218-02	UGM060504-01B	04. May 2006	Chamber 3	1	1	0	0	0	0	0	1	0	0	0	1	0	1	0	0	0	0	0	1	1	1	0	0	0	1	1	1	0	0	0	0	1	0	1	0	0	0
UGM051218-02	UGM060504-01C	04. May 2006	Chamber 4	1	1	0	0	0	0	0	1	0	0	0	1	0	1	0	0	0	0	0	1	1	1	0	0	0	1	1	1	0	0	0	0	1	0	1	0	0	0
UGM051218-02	UGM060621-01A	21. Jun. 2006	Chamber 5	1	1	0	0	0	0	0	1	0	0	0	1	0	1	0	0	0	0	0	1	1	1	0	0	0	1	1	1	0	0	0	0	1	0	1	0	0	0
UGM051218-02	UGM060621-01B	21. Jun. 2006	Chamber 6	1	1	0	0	0	0	0	1	0	0	0	1	0	1	0	0	0	0	0	1	1	1	0	0	0	1	1	1	0	0	0	0	1	0	1	0	0	0
UGM051218-02	UGM060621-01C	21. Jun. 2006	Chamber 7	1	1	0	0	0	0	0	1	0	0	0	1	0	1	0	0	0	0	0	1	1	1	0	0	0	1	1	1	0	0	0	0	1	0	1	0	0	0
UGM051218-02	AR060930-01	30. Sep. 2006	Chamber 8	1	1	0	0	0	0	0	1	0	0	0	1	0	1	0	0	0	0	0	1	1	1	0	0	0	1	1	1	0	0	0	0	1	0	1	0	0	0
AR060123-01	AR060123-01	23. Jan. 2006	Chamber 1	1	0	0	1	0	0	0	0	1	0	0	1	0	1	1	0	0	0	1	1	1	0	1	0	0	1	1	1	1	1	0	1	0	0	1	1	0	1
AR060123-01	AR060327-03	27. Mar. 2006	Chamber 2	1	0	0	1	0	0	0	0	1	0	0	1	0	1	1	0	0	0	1	1	1	0	1	0	0	1	1	1	1	1	0	1	0	0	1	1	0	1
AR060123-01	UGM060622-02	22. Jun. 2006	Chamber 3	1	0	0	1	0	0	0	0	1	0	0	1	0	1	1	0	0	0	1	1	1	0	1	0	0	1	1	1	1	1	0	1	0	0	1	1	0	1
UGM060121-02	UGM060121-02	21. Jan. 2006	Chamber 1	1	0	0	1	0	1	1	0	0	1	0	1	0	1	0	0	0	1	0	1	1	0	1	0	0	1	1	1	0	1	1	0	0	1	0	0	1	1
UGM060121-02	UGM060121-02a	21. Jan. 2006	Chamber 2	1	0	0	1	0	1	1	0	0	1	0	1	0	1	0	0	0	1	0	1	1	0	1	0	0	1	1	1	0	1	1	0	0	1	0	0	1	1
UGM060121-02	UGM060327-02	27. Mar. 2006	Chamber 3	1	0	0	1	0	1	1	0	0	1	0	1	0	1	0	0	0	1	0	1	1	0	1	0	0	1	1	1	0	1	1	0	0	1	0	0	1	1
UGM060121-02	UGM060621-02	21. Jun. 2006	Chamber 4	1	0	0	1	0	1	1	0	0	1	0	1	0	1	0	0	0	1	0	1	1	0	1	0	0	1	1	1	0	1	1	0	0	1	0	0	1	1
UGM060121-02	UGM061004-01	04. Oct. 2006	Chamber 5	1	0	0	1	0	1	1	0	0	1	0	1	0	1	0	0	0	1	0	1	1	0	1	0	0	1	1	1	0	1	1	0	0	1	0	0	1	1
UGM060511-01	UGM060511-01	11. May 2006	Chamber 1	1	0	0	1	0	0	0	0	1	0	0	1	0	1	1	1	0	0	1	1	1	0	1	0	0	1	1	1	1	1	1	0	0	0	1	1	0	1
UGM060511-01	UGM070517-01	17. May 2007	Chamber 2	1	0	0	1	0	0	0	0	1	0	0	1	0	1	1	1	0	0	1	1	1	0	1	0	0	1	1	1	1	1	1	0	0	0	1	1	0	1
UGM061223-01	UGM061223-01A	23. Dec. 2006	Chamber 1	1	1	0	1	0	0	0	1	0	0	0	1	0	1	1	0	0	0	1	1	1	0	1	0	0	1	0	1	1	0	1	0	0	0	1	1	0	1
UGM061223-01	UGM061223-01B	23. Dec. 2006	Chamber 2	1	1	0	1	0	0	0	1	0	0	0	1	0	1	1	0	0	0	1	1	1	0	1	0	0	1	0	1	1	0	1	0	0	0	1	1	0	1
UGM060517-01	UGM060517-01A	17. May 2006	Chamber 1	1	1	0	0	0	0	0	1	0	0	0	1	0	1	0	0	0	0	0	1	1	1	0	0	0	1	1	1	0	0	0	0	1	0	1	0	0	0
UGM060517-01	UGM060517-01B	17. May 2006	Chamber 2	fail	fail	fail	fail	fail	fail	fail	fail	fail	0	0	1	0	1	0	0	0	0	0	1	1	1	0	0	0	1	1	1	0	fail	fail	fail	fail	fail	1	0	0	0

B430 (TC repeat)											A128 (AC repeat)							B150 (CT repeat)													A1151 (CA repeat)										B319 (AG repeat)			
145	147	149	151	153	155	157	159	161	163	165	202	204	206	208	210	213	215	161	163	165	166	168	171	173	175	177	179	181	184	186	152	154	155	157	159	161	163	165	167	169	195	197	199	201
				stut	stut						stut	stut		stut		stut								stut														stut						
0	0	0	1	0	0	1	0	0	0	1	0	1	1	0	0	0	0	1	0	0	0	0	0	0	1	0	1	0	0	0	fail	fail	fail	fail	fail	fail	fail	fail	fail	fail	1	0	0	0
0	0	0	1	0	0	1	0	0	0	1	0	1	1	0	0	0	0	1	0	0	0	0	0	0	1	0	1	0	0	0	0	0	0	0	0	0	1	0	1	0	1	0	0	0
0	0	0	1	0	0	1	0	0	0	1	0	1	1	0	0	0	0	1	0	0	0	0	0	0	1	0	1	0	0	0	0	0	0	0	0	0	1	0	1	0	1	0	0	0
0	0	0	1	0	0	1	0	0	0	1	0	1	1	0	0	0	0	1	0	0	0	0	0	0	1	0	1	0	0	0	0	0	0	0	0	0	1	0	1	0	1	0	0	0
0	0	0	1	0	0	1	0	0	0	1	0	1	1	0	0	0	0	1	0	0	0	0	0	0	1	0	1	0	0	0	0	0	0	0	0	0	1	0	1	0	1	0	0	0
0	0	0	1	0	0	1	0	0	0	1	0	1	1	0	0	0	0	1	0	0	0	0	0	0	1	0	1	0	0	0	0	0	0	0	0	0	1	0	1	0	1	0	0	0
0	0	0	1	0	0	1	0	0	0	1	0	1	1	0	0	0	0	1	0	0	0	0	0	0	1	0	1	0	0	0	0	0	0	0	0	0	1	0	1	0	1	0	0	0
0	0	0	1	0	0	1	0	0	0	1	0	1	1	0	0	0	0	1	0	0	0	0	0	0	1	0	1	0	0	0	0	0	0	0	0	0	1	0	1	0	1	0	0	0
0	0	0	1	0	0	1	0	0	0	1	0	1	1	0	0	0	0	1	0	0	0	0	0	0	1	0	1	0	0	0	0	0	0	0	0	0	1	0	1	0	1	0	0	0
0	0	0	1	0	0	1	0	0	0	1	0	1	1	0	0	0	0	1	0	0	0	0	0	0	1	0	1	0	0	0	0	0	0	0	0	0	1	0	1	0	1	0	0	0
0	0	0	1	0	0	1	0	0	0	1	0	1	1	0	0	0	0	1	0	0	0	0	0	0	1	0	1	0	0	0	0	0	0	0	0	0	1	0	1	0	1	0	0	0
0	0	0	1	0	0	1	0	0	0	1	0	1	1	0	0	0	0	1	0	0	0	0	0	0	1	0	1	0	0	0	0	0	0	0	0	0	1	0	1	0	1	0	0	0
0	1	0	1	0	1	1	0	0	0	0	1	1	0	1	1	0	1	1	0	0	0	0	0	0	1	0	0	1	0	0	1	1	0	0	0	1	0	1	1	0	0	0	1	0
0	1	0	1	0	1	1	0	0	0	0	1	1	0	1	1	0	1	fail	fail	fail	fail	fail	fail	fail	fail	fail	fail	fail	fail	fail	fail	fail	fail	fail	fail	fail	fail	fail	fail	fail	0	0	1	0
0	1	0	1	0	1	1	0	0	0	0	1	1	0	1	1	0	1	1	0	0	0	0	0	0	1	0	0	1	0	0	1	1	0	0	0	1	0	1	1	0	0	0	1	0
1	1	0	0	0	0	1	0	1	1	0	0	1	1	1	0	0	0	1	0	0	0	0	0	0	1	0	0	1	0	1	0	0	0	0	0	0	1	1	1	1	0	1	0	0
fail	fail	fail	fail	fail	fail	fail	fail	fail	fail	fail	fail	fail	fail	fail	fail	fail	fail	fail	fail	fail	fail	fail	fail	fail	fail	fail	fail	fail	fail	fail	fail	fail	fail	fail	fail	fail	fail	fail	fail	fail	0	1	0	0
1	1	0	0	0	0	1	0	1	1	0	0	1	1	1	0	0	0	1	0	0	0	0	0	0	1	0	0	1	0	1	0	0	0	0	0	0	1	1	1	1	0	1	0	0
1	1	0	0	0	0	1	0	1	1	0	0	1	1	1	0	0	0	1	0	0	0	0	0	0	1	0	0	1	0	1	0	0	0	0	0	0	1	1	1	1	0	1	0	0
1	1	0	0	0	0	1	0	1	1	0	0	1	1	1	0	0	0	1	0	0	0	0	0	0	1	0	0	1	0	1	0	0	0	0	0	0	1	1	1	1	0	1	0	0
0	1	0	1	0	1	1	0	0	0	0	1	0	0	1	1	0	0	1	0	0	0	0	0	0	1	0	0	1	0	0	1	0	0	0	0	1	0	1	1	0	0	0	0	1
0	1	0	1	0	1	1	0	0	0	0	1	0	0	1	1	0	0	1	0	0	0	0	0	0	1	0	0	1	0	0	1	0	0	0	0	1	0	1	1	0	0	0	0	1
0	1	0	1	0	1	1	0	0	0	0	1	1	0	1	1	0	0	1	0	0	0	0	0	0	1	0	1	1	0	0	1	1	0	0	0	1	1	1	1	0	1	0	1	0
0	1	0	1	0	1	1	0	0	0	0	1	1	0	1	1	0	0	1	0	0	0	0	0	0	1	0	1	1	0	0	1	1	0	0	0	1	1	1	1	0	1	0	1	0
0	0	0	1	0	0	1	0	0	0	0	0	1	1	0	0	0	0	1	0	0	0	0	0	0	1	0	1	0	0	0	0	0	0	0	0	0	1	0	1	0	1	0	0	0
0	0	0	1	0	0	1	0	0	0	0	0	1	1	0	0	0	0	1	0	0	0	0	0	0	1	0	1	0	0	0	0	0	0	0	0	0	1	0	1	0	1	0	0	0
