# Supplementary material for: Monoculture of Leafcutter Ant Gardens
Source: PLoS One. 2010 Sep 10;5(9):e12668. doi: 10.1371/journal.pone.0012668 (PMC2937030; doi:10.1371/journal.pone.0012668)
Supplement: Table S3 — Within-nest Attamyces diversity for Atta cephalotes. (0.30 MB PDF) [file pone.0012668.s004.pdf]

Table S3. Microsatellite marker profiles of *Attamyces* from gardens of six *Atta cephalotes* colonies excavated in 2003.
Information on the *Attamyces* genotyping for each colony of *A. cephalotes* is presented on separate sheets in this file.

LEGEND (for the following six sheets: Colony 2, Colony 6, Colony 8, Colony 9, Colony 12, Colony 13)

**fail**	**= PCR for locus failed completely**
**yellow**	**= allele dropout, attributed to PCR artifact**
**orange**	**= possible mutation**

Brief Methods (details of methods are in main text and the supplemental information):
The mound of each nest was divided into four quadrants (listed in column A in the following Excel sheets for each of the six excavated nests).
Excavations aimed to access gardens from each quadrant, but in some quadrants no gardens could be found.
Between 3-12 gardens (listed in column B) were sampled per quadrant, depending on the success of the excavation in a given quadrant.
For each garden, three garden-fragments (listed in column C) were preserved for microsatellite genoypting.
Depth of each excavated garden is listed in column D.
Microsatellite DNA genotyping information is listed in columns E-N for the 10 loci screened.

Table S3: Colony 2

GPS: N09º 09' 07.7" = N09.1521°
W79º 44' 09.8" = W79.7361°

Mound diameter: 22m x 14m

Nest Quadrant	Garden ID	Sample Name	Excavation Depth (cm)	A128 $(AC)_n$	A435 $(GT)_n$	A1132 $(TCA)_n$	B12 $(GA)_n$	B150 $(CT)_n$	B312 $(GA)_n$	B430 $(TC)_n$	C117 $(CAG)_n(CAC)_n$	C1133 $(TGC)_n$-A-$(GGA)_n$	D115 $(TCA)_n$-TCG-$(TCA)_n$
Quadrant I		2IA		222, 224	234	202, 214	232	178, 182	168, 176	168, 174	264	233, 245	251, 266
	I-A	2IA	40-50	222, 224	234	202, 214	232	178, 182, 186	168, 176	168, 174	264	233, 245	251, 266
		2IA		222, 224	234	202, 214	232	178, 182, 186	168, 176	168, 174	264	233, 245	251, 266
		2IB		222, 224	234	202, 214	232	178, 182, 186	168, 176	168, 174	264	233, 245	251, 266
	I-B	2IB	40-50	222, 224	234	202, 214	232	178, 182, 186	168, 176	168, 174	264	233, 245	251, 266
		2IB		222, 224	234	202, 214	232	178, 182, 186	168, 176	168, 174	264	233, 245	251, 266
		2IC		222, 224	234	202, 214	232	178, 182, 186	168, 176	168, 174	264	233, 245	251, 266
	I-C	2IC	30-40	222, 224	234	202, 214	232	178, 182, 186	168, 176	168, 174	264	233, 245	251, 266
		2IC		222, 224	234	202, 214	232	178, 182, 186	168, 176	168, 174	264	233, 245	251, 266
		2ID		222, 224	234	202, 214	232	178, 182, 186	168, 176	168, 174	264	233, 245	251, 266
	I-D	2ID	30-40	222, 224	234	202, 214	232	178, 182, 186	168, 176	168, 174	264	233, 245	251, 266
		2ID		222, 224	234	202, 214	232	178, 182, 186	168, 176	168, 174	264	233, 245	251, 266
		2IE		222, 224	234	202, 214	232	178, 182, 186	168, 176	168, 174	264	233, 245	251, 266
	I-E	2IE	20-30	222, 224	234	202, 214	232	178, 182, 186	168, 176	168, 174	264	233, 245	251, 266
		2IE		222, 224	234	202, 214	232	178, 182, 186	168, 176	168, 174	264	233, 245	251, 266
		2IF		222, 224	234	202, 214	232	178, 182, 186	168, 176	168, 174	264	233, 245	251, 266
	I-F	2IF	70-80	222, 224	234	202, 214	232	178, 182, 186	168, 176	168, 174	264	233, 245	251, 266
		2IF		222, 224	234	202, 214	232	178, 182, 186	168, 176	168, 174	264	233, 245	251, 266
		2IG		222, 224	234	202, 214	232	178, 182, 186	168, 176	168, 174	264	233, 245	251, 266
	I-G	2IG	50-60	222, 224	234	202, 214	232	178, 182, 186	168, 176	168, 174	264	233, 245	251, 266
		2IG		222, 224	234	202, 214	232	178, 182, 186	168, 176	168, 174	264	233, 245	251, 266
		2IH		222, 224	234	202, 214	232	178, 182, 186	168, 176	168, 174	264	233, 245	251, 266
	I-H	2IH	50-60	222, 224	234	202, 214	232	178, 182, 186	168, 176	168, 174	264	233, 245	251, 266
		2IH		222, 224	234	202, 214	232	178, 182, 186	168, 176	168, 174	264	233, 245	251, 266
		2I_I		222, 224	234	202, 214	232	178, 182, 186	168, 176	168, 174	264	233, 245	251, 266
	I-I	2I_I	40-50	222, 224	234	202, 214	232	178, 182, 186	168, 176	168, 174	264	233, 245	251, 266
		2I_I		222, 224	234	202, 214	232	178, 182, 186	168, 176	168, 174	264	233, 245	251, 266
		2I_J		222, 224	234	202, 214	232	178, 182, 186	168, 176	168, 174	264	233, 245	251, 266
	I-J	2I_J	40-50	222, 224	234	202, 214	232	178, 182, 186	168, 176	168, 174	264	233, 245	251, 266
		2I_J		222, 224	234	202, 214	232	178, 182, 186	168, 176	168, 174	264	233, 245	251, 266
Quadrant II		2IIA		222, 224	234	202, 214	232	178, 182, 186	168, 176	168, 174	264	233, 245	251, 266
	II-A	2IIA	40-50	222, 224	234	202, 214	232	178, 182, 186	168, 176	168, 174	264	233, 245	251, 266
		2IIA		222, 224	234	202, 214	232	178, 182, 186	168, 176	168, 174	264	233, 245	251, 266
		2IIB		222, 224	234	202, 214	232	178, 182, 186	168, 176	168, 174	264	233, 245	251, 266
	II-B	2IIB	60-70	222, 224	234	202, 214	232	178, 182, 186	168, 176	168, 174	264	233, 245	251, 266
		2IIB		222, 224	234	202, 214	232	178, 182, 186	168, 176	168, 174	264	233, 245	251, 266
		2IIC		222, 224	234	202, 214	232	178, 182, 186	168, 176	168, 174	264	233, 245	251, 266
	II-C	2IIC	70-80	222, 224	234	202, 214	232	178, 182, 186	168, 176	168, 174	264	233, 245	251, 266
		2IIC		222, 224	234	202, 214	232	178, 182, 186	168, 176	168, 174	264	233, 245	251, 266
Quadrant III		2IIIA		222, 224	234	202, 214	232	178, 182, 186	168, 176	168, 174	264	233, 245	251, 266
	III-A	2IIIA	40-50	222, 224	234	202, 214	232	178, 182, 186	168, 176	168, 174	264	233, 245	251, 266
		2IIIA		222, 224	234	202, 214	232	178, 182, 186	168, 176	168, 174	264	233, 245	251, 266
		2IIIB		222, 224	234	202, 214	232	178, 182, 186	168, 176	168, 174	264	233, 245	251, 266
	III-B	2IIIB	70-80	222, 224	234	202, 214	232	178, 182, 186	168, 176	168, 174	264	233, 245	251, 266
		2IIIB		222, 224	234	202, 214	232	178, 182, 186	168, 176	168, 174	264	233, 245	251, 266
		2IIIC		222, 224	234	202, 214	232	178, 182, 186	168, 176	168, 174	264	233, 245	251, 266
	III-C	2IIIC	60-70	222, 224	234	202, 214	232	178, 182, 186	168, 176	168, 174	264	233, 245	251, 266
		2IIIC		222, 224	234	202, 214	232	178, 182, 186	168, 176	168, 174	264	233, 245	251, 266
Quadrant IV		2IVA		222, 224	234	202, 214	232	178, 182, 186	168, 176	168, 174	264	233, 245	251, 266
	IV-A	2IVA	30-40	222, 224	234	202, 214	232	178, 182, 186	168, 176	168, 174	264	233, 245	251, 266
		2IVA		222, 224	234	202, 214	232	178, 182, 186	168, 176	168, 174	264	233, 245	251, 266
		2IVB		222, 224	234	202, 214	232	178, 182, 186	168, 176	168, 174	264	233, 245	251, 266
	IV-B	2IVB	30-40	222, 224	234	202, 214	232	178, 182, 186	168, 176	168, 174	264	233, 245	251, 266
		2IVB		222, 224	234	202, 214	232	178, 182, 186	168, 176	168, 174	264	233, 245	251, 266
		2IVC		222, 224	234	202, 214	232	178, 182, 186	168, 176	168, 174	264	233, 245	251, 266
	IV-C	2IVC	40-50	222, 224	234	202, 214	232	178, 182, 186	168, 176	168, 174	264	233, 245	251, 266
		2IVC		222, 224	234	202, 214	232	178, 182, 186	168, 176	168, 174	264	233, 245	251, 266
		2IVD		222, 224	234	202, 214	232	178, 182, 186	168, 176	168, 174	264	233, 245	251, 266
	IV-D	2IVD	20-30	222, 224	234	202, 214	232	178, 182, 186	168, 176	168, 174	264	233, 245	251, 266
		2IVD		222, 224	234	202, 214	232	178, 182, 186	168, 176	168, 174	264	233, 245	251, 266
		2IVE		222, 224	234	202, 214	232	178, 182, 186	168, 176	168, 174	264	233, 245	251, 266
	IV-E	2IVE	30-40	222, 224	234	202, 214	232	178, 182, 186	168, 176	168, 174	264	233, 245	251, 266
		2IVE		222, 224	234	202, 214	232	178, 182, 186	168, 176	168, 174	264	233, 245	251, 266
		2IVF		222, 224	234	202, 214	232	178, 182, 186	168, 176	168, 174	264	233, 245	251, 266
	IV-F	2IVF	20-30	222, 224	234	202, 214	232	178, 182, 186	168, 176	168, 174	264	233, 245	251, 266
		2IVF		222, 224	234	202, 214	232	178, 182, 186	168, 176	168, 174	264	233, 245	251, 266
		2IVG		222, 224	234	202, 214	232	178, 182, 186	168, 176	168, 174	264	233, 245	251, 266
	IV-G	2IVG	50-60	222, 224	234	202, 214	232	178, 182, 186	168, 176	168, 174	264	233, 245	251, 266
		2IVG		222, 224	234	202, 214	232	178, 182, 186	168, 176	168, 174	264	233, 245	251, 266
		2IVH		222, 224	234	202, 214	232	178, 182, 186	168, 176	168, 174	264	233, 245	251, 266
	IV-H	2IVH	30-40	222, 224	234	202, 214	232	178, 182, 186	168, 176	168, 174	264	233, 245	251, 266
		2IVH		222, 224	234	202, 214	232	178, 182, 186	168, 176	168, 174	264	233, 245	251, 266
		2IV_I		222, 224	234	202, 214	232	178, 182, 186	168, 176	168, 174	264	233, 245	251, 266
	IV-I	2IV_I	50-60	222, 224	234	202, 214	232	178, 182, 186	168, 176	168, 174	264	233, 245	251, 266
		2IV_I		222, 224	234	202, 214	232	178, 182, 186	168, 176	168, 174	264	233, 245	251, 266

Table S3: Colony 6

GPS: N09º 08' 17.0" = N09.1381°
W79º 44' 09.8" = W79.7361°

Mound diameter: 25m x 11.5m

Nest Quadrant	Garden ID	Sample Name	Excavation Depth (cm)	A128 $(AC)_n$	A435 $(GT)_n$	A1132 $(TCA)_n$	B12 $(GA)_n$	B150 $(CT)_n$	B312 $(GA)_n$	B430 $(TC)_n$	C117 $(CAG)_n(CAC)_n$	C1133 $(TGC)_n$-A-$(GGA)_n$	D115 $(TCA)_n$-TCG-$(TCA)_n$
Quadrant I	I-A	6IA	40-50	222, 226	234	202, 214	232	178, 186	168, 176	164, 174	263	233, 245	251, 266
		6IA		222, 226	234	202, 214	232	178, 186	168, 176	164, 174	263	233, 245	251, 266
		6IA		222, 226	234	202, 214	232	178, 186	168, 176	164, 174	263	233, 245	251, 266
	I-B	6IB	50-60	222, 226	234	202, 214	232	178, 186	168, 176	164, 174	263	233, 245	251, 266
		6IB		222, 226	234	202, 214	232	178, 186	168, 176	164, 174	263	233, 245	251, 266
		6IB		222, 226	234	202, 214	232	178, 186	168, 176	164, 174	263	233, 245	251, 266
	I-C	6IC	30-40	222, 226	234	202, 214	232	178, 186	168, 176	164, 174	263	233, 245	251, 266
		6IC		222, 226	234	202, 214	232	178, 186	168, 176	164, 174	263	233, 245	251, 266
		6IC		222, 226	234	202, 214	232	178, 186	168, 176	164, 174	263	233, 245	251, 266
	I-D	6ID	20-30	222, 226	234	202, 214	232	178, 186	168, 176	164, 174	263	233, 245	251, 266
		6ID		222, 226	234	202, 214	232	178, 186	168, 176	164, 174	263	233, 245	251, 266
		6ID		222, 226	234	202, 214	232	178, 186	168, 176	164, 174	263	233, 245	251, 266
	I-E	6IE	60-70	222, 226	234	202, 214	232	178, 186	168, 176	164, 174	263	233, 245	251, 266
		6IE		222, 226	234	202, 214	232	178, 186	168, 176	164, 174	263	233, 245	251, 266
		6IE		fail	234	202, 214	232	178, 186	168, 176	164, 174	263	233, 245	251, 266
	I-F	6IF	30-40	222, 226	234	202, 214	232	178, 186	168, 176	164, 174	263	233, 245	251, 266
		6IF		222, 226	234	202, 214	232	178, 186	168, 176	164, 174	263	233, 245	251, 266
		6IF		222, 226	234	202, 214	232	178	168, 176	164, 174	263, 266	233, 245	251, 266
	I-G	6IG	50-60	222, 226	234	202, 214	232	178, 186	168, 176	164, 174	263	233, 245	251, 266
		6IG		222, 226	234	202, 214	232	178	168, 176	164, 174	263	233, 245	251, 266
		6IG		222, 226	234	202, 214	232	178, 186	168, 176	164, 174	263	233, 245	251, 266
	I-H	6IH	40-50	222, 226	234	202, 214	232	178, 186	168, 176	164, 174	263	233, 245	251, 266
		6IH		222, 226	234	202, 214	232	178, 186	168, 176	164, 174	263	233, 245	251, 266
		6IH		222, 226	234	202, 214	232	178, 186	168, 176	164, 174	263	233, 245	251, 266
Quadrant II	II-A	6IIA	20-30	222, 226	234	202, 214	232	178, 186	168, 176	164, 174	263	233, 245	251, 266
		6IIA		222, 226	234	202, 214	232	178, 186	168, 176	164, 174	263	233, 245	251, 266
		6IIA		222, 226	234	202, 214	232	178, 186	168, 176	164, 174	263	233, 245	251, 266
	II-B	6IIB	60-70	222, 226	234	202, 214	232	178, 186	168, 176	164, 174	263	233, 245	251, 266
		6IIB		222, 226	234	202, 214	232	178, 186	168, 176	164, 174	263	233, 245	251, 266
		6IIB		222, 226	234	202, 214	232	178, 186	168, 176	164, 174	263	233, 245	251, 266
	II-C	6IIC	60-70	222, 226	234	202, 214	232	178, 186	168, 176	164, 174	263	233, 245	251, 266
		6IIC		222, 226	234	202, 214	232	178, 186	168, 176	164, 174	263	233, 245	251, 266
		6IIC		222, 226	234	202, 214	232	178, 186	168, 176	164, 174	263	233, 245	251, 266
	II-D	6IID	20-30	222, 226	234	202, 214	232	178, 186	168, 176	164, 174	263	233, 245	251, 266
		6IID		222, 226	234	202, 214	232	178, 186	168, 176	164, 174	263	233, 245	251, 266
		6IID		222, 226	234	202, 214	232	178, 186	168, 176	164, 174	263	233, 245	251, 266
	II-E	6IIE	30-40	222, 226	234	202, 214	232	178, 186	168, 176	164, 174	263	233, 245	251, 266
		6IIE		222, 226	234	202, 214	232	178, 186	168, 176	164, 174	263	233, 245	251, 266
		6IIE		222	234	202, 214	232	178, 186	168, 176	164, 174	263	233, 245	251, 266
	II-F	6IIF	40-50	222, 226	234	202, 214	232	178, 186	fail	164, 174	fail	233, 245	251, 266
		6IIF		222, 226	234	202, 214	232	178, 186	168, 176	164, 174	263	233, 245	251, 266
		6IIF		222, 226	234	202, 214	232	178	168, 176	164, 174	263	233, 245	251, 266
	II-G	6IIG	30-40	222, 226	234	202, 214	232	178, 186	168, 176	164, 174	263	233, 245	251, 266
		6IIG		222, 226	234	202, 214	232	178	168, 176	164, 174	263	233, 245	251, 266
		6IIG		222, 226	234	202, 214	232	178	168, 176	164, 174	263	233, 245	251, 266
	II-H	6IIH	50-60	222, 226	234	202, 214	232	178, 186	168, 176	164, 174	263	233, 245	251, 266
		6IIH		fail	234	202, 214	232	178, 186	168, 176	164, 174	263	233, 245	251, 266
		6IIH		222, 226	234	202, 214	232	178, 186	176, 180	164, 174	263	233, 245	251, 266
	II-I	6II_I	50-60	222, 226	234	202, 214	232	178, 186	168, 176	164, 174	263	233, 245	251, 266
		6II_I		222, 226	234	202, 214	232	178, 186	168, 176	164, 174	263	233, 245	251, 266
		6II_I		222, 226	234	202, 214	232	178, 186	168, 176	164, 174	263	233, 245	251, 266
		6II_I		222, 226	234	202, 214	232	178, 186	168, 176	164, 174	263	233, 245	251, 266
	II-J	6IIJ	50-60	222, 226	234	202, 214	232	178, 186	168, 176	164, 174	263	233, 245	251, 266
		6IIJ		222, 226	234	202, 214	232	178, 186	168, 176	164, 174	263	233, 245	251, 266
		6IIJ		222, 226	234	202, 214	232	178, 186	168, 176	164, 174	263	233, 245	251, 266
	II-K	6IIK	20-30	222, 226	234	202, 214	232	178, 186	168, 176	164, 174	263	233, 245	251, 266
		6IIK		222, 226	234	202, 214	232	178, 186	168, 176	164, 174	263	233, 245	251, 266
		6IIK		222, 226	234	202, 214	232	178, 186	168, 176	164, 174	263	233, 245	251, 266
	II-L	6IIL	50-60	222, 226	fail	202, 214	232	178, 186	168, 176	164, 174	263	233, 245	251, 266
		6IIL		222, 226	234	202, 214	232	178, 186	168, 176	164, 174	263	233, 245	251, 266
		6IIL		222, 226	234	202, 214	232	178	168, 176	164, 174	263	233, 245	251, 266
Quadrant III	III-A	6IIIA	30-40	222, 226	234	202, 214	232	178, 186	168, 176	164, 174	263	233, 245	251, 266
		6IIIA		222, 226	234	202, 214	232	178, 186	168, 176	164, 174	fail	233, 245	251, 266
		6IIIA		222, 226	234	202, 214	232	178, 186	168, 176	164, 174	263	233, 245	251, 266
	III-B	6IIIB	40-50	222, 226	234	202, 214	232	178, 186	168, 176	164, 174	263	233, 245	251, 266
		6IIIB		222, 226	234	202, 214	232	178, 186	168, 176	164, 174	263	233, 245	251, 266
		6IIIB		222, 226	234	202, 214	232	178, 186	168, 176	164, 174	263	233, 245	251, 266
	III-C	6IIIC	40-50	222, 226	234	202, 214	232	178, 186	168, 176	164, 174	263	233, 245	251, 266
		6IIIC		222, 226	234	202, 214	232	178, 186	168, 176	164, 174	263	233, 245	251, 266
		6IIIC		222, 226	234	202, 214	232	178, 186	168, 176	164, 174	263	233, 245	251, 266
	III-D	6IIID	30-40	222, 226	234	202, 214	232	178, 186	168, 176	164, 174	263	233, 245	251, 266
		6IIID		222, 226	234	202, 214	232	178, 186	168, 176	164, 174	263	233, 245	251, 266
		6IIID		222, 226	234	202, 214	232	178, 186	168, 176	164, 174	263	233, 245	251, 266
	III-E	6IIIE	40-50	222, 226	234	202, 214	232	178, 186	168, 176	164, 174	263	233, 245	251, 266
		6IIIE		222, 226	234	202, 214	232	178, 186	168, 176	164, 174	263	233, 245	251, 266
		6IIIE		222, 226	234	202, 214	232	178, 186	168, 176	164, 174	263	233, 245	251, 266

Table S3: Colony 8

GPS: N09° 08' 52.2" = N09.1478°
W79° 43' 55.4" = W79.7321°

Mound diameter: 14m x 14m

Nest Quadrant	Garden ID	Sample Name	Excavation Depth (cm)	A128 $(AC)_n$	A435 $(GT)_n$	A1132 $(TCA)_n$	B12 $(GA)_n$	B150 $(CT)_n$	B312 $(GA)_n$	B430 $(TC)_n$	C117 $(CAG)_n(CAC)_n$	C1133 $(TGC)_n$-A-$(GGA)_n$	D115 $(TCA)_n$-TCG-$(TCA)_n$
Quadrant I	I-A	8IA	40-50	222, 226	234	202, 214	232	178, 186	168,176	168, 174	264	233, 245	251, 266
		8IA		222, 226	234	202, 214	232	178, 186	168,176	168, 174	264	233, 245	251, 266
		8IA		222, 226	234	202, 214	232	178, 186	168,176	168, 174	264	233, 245	251, 266
	I-B	8IB	40-50	222, 226	234	202, 214	232	178, 186	168,176	168, 174	264	233, 245	251, 266
		8IB		222, 226	234	202, 214	232	178, 186	168,176	168, 174	264	233, 245	251, 266
		8IB		222, 226	234	202, 214	232	178	168,176	168, 174	264	233, 245	251, 266
	I-C	8IC	40-50	222, 226	234	202, 214	232	178, 186	168,176	168, 174	264	233, 245	251, 266
		8IC		222, 226	234	202, 214	232	178, 186	168,176	168, 174	264	233, 245	251, 266
		8IC		222, 226	234	202, 214	232	178, 186	168,176	168, 174	264	233, 245	251, 266
	I-D	8ID	40-50	222, 226	234	202, 214	232	178, 186	168,176	168, 174	264	233, 245	251, 266
		8ID		222, 226	234	202, 214	232	178, 186	168,176	168, 174	264	233, 245	251, 266
		8ID		222, 226	234	202, 214	232	178, 186	168,176	168, 174	264	233, 245	251, 266
	I-E	8IE	50-60	222, 226	234	202, 214	232	178, 186	168,176	168, 174	264	233, 245	251, 266
		8IE		222, 226	234	202, 214	232	178	168,176	168, 174	264	233, 245	251, 266
		8IE		222, 226	234	202, 214	232	178, 186	168,176	168, 174	264	fail	251, 266
	I-F	8IF	40-50	222, 226	234	202, 214	232	178, 186	168,176	168, 174	264	233, 245	251, 266
		8IF		222, 226	234	202, 214	232	178, 186	168,176	168, 174	264	233, 245	251, 266
		8IF		222, 226	234	202, 214	232	178, 186	168,176	168, 174	264	233, 245	251, 266
	I-G	8IG	30-40	222, 226	234	202, 214	232	178, 186	168,176	168, 174	264	233, 245	251, 266
		8IG		222, 226	234	202, 214	232	178, 186	168,176	168, 174	264	233, 245	251, 266
		8IG		222, 226	234	202, 214	232	178, 186	168,176	168, 174	264	233, 245	251, 266
	I-H	8IH	10-20	222, 226	234	202, 214	232	178, 186	168,176	168, 174	264	233, 245	251, 266
		8IH		222, 226	234	202, 214	232	178, 186	168,176	168, 174	264	233, 245	251, 266
		8IH		222, 226	234	202, 214	232	178, 186	168,176	168, 174	264	233, 245	251, 266
	I-I	8I_I	20-30	222, 226	234	202, 214	232	178, 186	168,176	168, 174	264	233, 245	251, 266
		8I_I		222, 226	234	202, 214	232	178, 186	168,176	168, 174	264	233, 245	251, 266
	I-K	8IK	80-90	222	234	202, 208	232	178	168,176	168, 174	264	233, 245	251, 266
		8IK		222	234	202, 214	232	178, 186	168,176	168, 174	264	233, 245	251, 266
		8IK		222, 226	234	202, 214	232	178	168,176	168, 174	264	233, 245	251, 266
	I-J	8IJ	80-90	224, 226	232	202, 214	232	178	168,176	168, 174	264	233, 245	251, 266
		8IJ		222, 226	234	202, 208	232	178, 186	168	168, 174	264	233, 245	251, 266
		8IJ		222, 226	234	202, 214	232	178, 186	168,176	168, 174	264	233, 245	251, 266
Quadrant II	II-A	8IIA	20-30	222, 226	234	202, 214	232	178, 186	168,176	168, 174	264	233, 245	251, 266
		8IIA		222, 226	234	202, 214	232	178, 186	168,176	168, 174	264	233, 245	251, 266
		8IIA		222, 226	234	202, 214	232	178, 186	168,176	168, 174	264	233, 245	251, 266
	II-B	8IIB	20-30	222, 226	234	202	232	178	168,176	fail	264	233, 245	251, 266
		8IIB		222, 226	234	202, 214	232	178, 186	168,176	168, 174	264	233, 245	251, 266
		8IIB		222, 226	234	202, 214	232	178	168,176	168, 174	264	233, 245	251, 266
	II-C	8IIC	30-40	222, 226	234	202, 214	232	178	168,176	168, 174	264	233, 245	251, 266
		8IIC		222, 226	234	202, 214	232	178, 186	168,176	168, 174	264	233, 245	251, 266
		8IIC		222, 226	234	202, 214	232	178, 186	168,176	168, 174	264	233, 245	251, 266
	II-D	8IID	20-30	222, 226	234	202, 214	232	178, 186	168,176	168, 174	264	233, 245	251, 266
		8IID		222, 226	234	202, 214	232	178	168,176	168, 174	264	233, 245	251, 266
		8IID		222, 226	234	202, 214	232	178, 186	168,176	168, 174	264	233, 245	251, 266
	II-E	8IIE	30-40	222, 226	234	202	232	178, 186	168,176	168, 174	264	233, 245	251, 266
		8IIE		222, 226	234	202, 214	232	178, 186	168,176	168, 174	264	233, 245	251, 266
		8IIE		222, 226	234	202, 214	fail	178, 186	168,176	168, 174	264	233, 245	251, 266
	II-F	8IIF	50-60	222, 226	234	202, 214	232	178, 186	168,176	168, 174	264	233, 245	251, 266
		8IIF		222, 226	234	202, 214	232	178, 186	168,176	168, 174	264	233, 245	251, 266
		8IIF		222, 226	234	202, 214	232	178, 186	168,176	168, 174	264	233, 245	251, 266
Quadrant III	III-A	8IIIA	30-40	222, 226	234	202, 214	232	178, 186	168,176	168, 174	264	233, 245	251, 266
		8IIIA		222, 226	234	202, 214	232	178, 186	168,176	168, 174	264	233, 245	251, 266
		8IIIA		222, 226	234	202, 214	232	178, 186	168,176	168, 174	264	233, 245	251, 266
	III-B	8IIIB	30-40	222, 226	234	202, 214	232	178	168,176	168, 174	264	233, 245	251, 266
		8IIIB		222, 226	234	202, 214	232	178, 186	168,176	168, 174	264	233, 245	251, 266
		8IIIB		222, 226	234	202, 214	232	178, 186	168,176	168, 174	264	233, 245	251, 266
	III-C	8IIIC	30-40	224, 226	234	202, 214	232	178, 186	168,176	168, 174	264	233, 245	251, 266
		8IIIC		222, 226	234	202, 208	232	178, 186	168,176	168, 174	264	233, 245	251, 266
		8IIIC		222, 226	234	202, 214	232	178, 186	168,176	168, 174	264	233, 245	251, 266
	III-D	8IIID	30-40	222, 226	234	202, 214	232	178, 186	168,176	168, 174	264	233, 245	251, 266
		8IIID		222, 226	234	202, 214	232	178, 186	168,176	168, 174	264	233, 245	251, 266
		8IIID		224, 226	234	202, 214	232	178, 186	168,176	168, 174	264	fail	251, 266
	III-E	8IIIE	30-40	222, 226	234	202, 214	232	178, 186	168,176	168, 174	264	233, 245	251, 266
		8IIIE		222, 226	234	202, 214	232	178, 186	168,176	168, 174	264	233, 245	251, 266
		8IIIE		222, 226	234	202, 214	232	178, 186	168,176	168, 174	264	233, 245	251, 266
	III-F	8IIIF	30-40	222, 226	234	202, 214	232	178, 186	168,176	168, 174	264	233, 245	251, 266
		8IIIF		222, 226	234	202, 214	232	178, 186	168,176	168, 174	264	233, 245	251, 266
		8IIIF		222, 226	234	202, 214	232	178, 186	168,176	168, 174	264	233, 245	251, 266

Table S3: Colony 9

GPS: N09º 09' 35.04" = N09.1597°
W79º 44' 23.46" = W79.7399°

Mound diameter: 20m x 18m

Nest Quadrant	Garden ID	Sample Name	Excavation Depth (cm)	A128 $(AC)_n$	A435 $(GT)_n$	A1132 $(TCA)_n$	B12 $(GA)_n$	B150 $(CT)_n$	B312 $(GA)_n$	B430 $(TC)_n$	C117 $(CAG)_n(CAC)_n$	C1133 $(TGC)_n$-A-$(GGA)_n$	D115 $(TCA)_n$-TCG-$(TCA)_n$
		9IA		222, 228	232, 238	202, 208	232, 248	178, 194, 202	176, 194	164, 178	260, 266	248, 251	251, 254
	I-A	9IA	30-40	222, 228	232, 238	202, 208	232, 248	178, 194, 202	176, 194	164, 178	260, 266	248, 251	251, 254
		9IA		222, 228	232, 238	202, 208	232, 248	178, 194, 202	176, 194	164, 178	260, 266	248, 251	251, 254
		9IB		222, 228	232, 238	202, 208	232, 248	178, 194, 202	176, 194	164, 178	260, 266	248, 251	251, 254
	I-B	9IB	20-30	222, 228	232, 238	202, 208	232, 248	178, 194, 202	176, 194	164, 178	260, 266	248, 251	251, 254
		9IB		222, 228	232, 238	202, 208	232, 248	178, 194, 202	176, 194	164, 178	260, 266	248, 251	251, 254
		9IC		222, 228	232, 238	202, 208	232, 248	178, 194, 202	176, 194	164, 178	260, 266	248, 251	251, 254
	I-C	9IC	20-30	222, 228	232, 238	202, 208	232, 248	178, 194, 202	176, 194	164, 178	260, 266	248, 251	251, 254
		9IC		222, 228	232, 238	202, 208	232, 248	178, 194, 202	176, 194	164, 178	260, 266	248, 251	251, 254
		9ID		222, 228	232, 238	202, 208	232, 248	178, 194, 202	176, 194	164, 178	260, 266	248, 251	251, 254
	I-D	9ID	20-30	222, 228	232, 238	202, 208	232, 248	178, 194, 202	176, 194	164, 178	260, 266	248, 251	251, 254
		9ID		222, 228	232, 238	202, 208	232, 248	178, 194, 202	176, 194	164, 178	260, 266	248, 251	251, 254
		9IE		222, 228	232, 238	202, 208	232, 248	178, 194, 202	176, 194	164, 178	260, 266	248, 251	251, 254
Quadrant I	I-E	9IE	20-30	222, 228	232, 238	202, 208	232, 248	178, 194, 202	176, 194	164, 178	260, 266	248, 251	251, 254
		9IE		222, 228	232, 238	202, 208	232, 248	178, 194, 202	176, 194	164, 178	260, 266	248, 251	251, 254
		9IF		222, 228	232, 238	202, 208	232, 248	178, 194, 202	176, 194	164, 178	260, 266	248, 251	251, 254
	I-F	9IF	20-30	222, 228	232, 238	202, 208	232, 248	178, 194, 202	176, 194	164, 178	260, 266	248, 251	251, 254
		9IF		222, 228	232, 238	202, 208	232, 248	178, 194, 202	176, 194	164, 178	260, 266	248, 251	251, 254
		9IG		222, 228	232, 238	202, 208	232, 248	178, 194, 202	176, 194	164, 178	260, 266	248, 251	251, 254
	I-G	9IG	20-30	222, 228	232, 238	202, 208	232, 248	178, 194, 202	176, 194	164, 178	260, 266	248, 251	251, 254
		9IG		222, 228	232, 238	202, 208	232, 248	178, 194, 202	176, 194	164, 178	260, 266	248, 251	251, 254
		9IH		222, 228	232, 238	202, 208	232, 248	178, 194, 202	176, 194	164, 178	260, 266	248, 251	251, 254
	I-H	9IH	20-30	222, 228	232, 238	202, 208	232, 248	178, 194, 202	176, 194	164, 178	260, 266	248, 251	251, 254
		9IH		222, 228	232, 238	202, 208	232, 248	178, 194, 202	176, 194	164, 178	260, 266	248, 251	251, 254
		9I_I		222, 228	232, 238	202, 208	232, 248	178, 194, 202	176, 194	164, 178	260, 266	248, 251	251, 254
	I-I	9I_I	20-30	222, 228	232, 238	202, 208	232, 248	178, 194, 202	176, 194	164, 178	260, 266	248, 251	251, 254
		9I_I		222, 228	232, 238	202, 208	232, 248	178, 194, 202	176, 194	164, 178	260, 266	248, 251	251, 254
		9IIA		222, 228	232, 238	202, 208	232, 248	178, 194, 202	176, 194	164, 178	260, 266	248, 251	251, 254
	II-A	9IIA	20-30	222, 228	232, 238	202, 208	232, 248	178, 194, 202	176, 194	164, 178	260, 266	248, 251	251, 254
		9IIA		222, 228	232, 238	202, 208	232, 248	178, 194, 202	176, 194	164, 178	260, 266	248, 251	251, 254
		9IIB		222, 228	232, 238	202, 208	232, 248	178, 194, 202	176, 194	164, 178	260, 266	248, 251	251, 254
	II-B	9IIB	15-25	222, 228	232, 238	202, 208	232, 248	178, 194, 202	176, 194	164, 178	260, 266	248, 251	251, 254
		9IIB		222, 228	232, 238	202, 208	232, 248	178, 194, 202	176, 194	164, 178	260, 266	248, 251	251, 254
		9IIC		222, 228	232, 238	202, 208	232, 248	178, 194, 202	176, 194	164, 178	260, 266	248, 251	251, 254
	II-C	9IIC	surface to 10	222, 228	232, 238	202, 208	232, 248	178, 194, 202	176, 194	164, 178	260, 266	248, 251	251, 254
		9IIC		222, 228	232, 238	202, 208	232, 248	178, 194, 202	176, 194	164, 178	260, 266	248, 251	251, 254
		9IID		222, 228	232, 238	202, 208	232, 248	178, 194, 202	176, 194	164, 178	260, 266	248, 251	251, 254
Quadrant II	II-D	9IID	20-30	222, 228	232, 238	202, 208	232, 248	178, 194, 202	176, 194	164, 178	260, 266	248, 251	251, 254
		9IID		222, 228	232, 238	202, 208	232, 248	178, 194, 202	176, 194	164, 178	260, 266	248, 251	251, 254
		9IIE		222, 228	232, 238	202, 208	232, 248	178, 194, 202	176, 194	164, 178	260, 266	248, 251	251, 254
	II-E	9IIE	20-30	222, 228	232, 238	202, 208	232, 248	178, 194, 202	176, 194	164, 178	260, 266	248, 251	251, 254
		9IIE		222, 228	232, 238	202, 208	232, 248	178, 194, 202	176, 194	164, 178	260, 266	248, 251	251, 254
		9IIF		222, 228	232, 238	202, 208	232, 248	178, 194, 202	176, 194	164, 178	260, 266	248, 251	251, 254
	II-F	9IIF	20-30	222, 228	232, 238	202, 208	232, 248	178, 194, 202	176, 194	164, 178	260, 266	248, 251	251, 254
		9IIF		222, 228	232, 238	202, 208	232, 248	178, 194, 202	176, 194	164, 178	260, 266	248, 251	251, 254
		9IIG		222, 228	232, 238	202, 208	232, 248	178, 194, 202	176, 194	164, 178	260, 266	248, 251	251, 254
	II-G	9IIG	20-30	222, 228	232, 238	202, 208	232, 248	fail	176, 194	164, 178	260, 266	248, 251	251, 254
		9IIG		222, 228	232, 238	202, 208	232, 248	178, 194, 202	176, 194	164, 178	260, 266	248, 251	251, 254
	III-A	9IIIA	30-40	222, 228	232, 238	202, 208	232, 248	178, 194, 202	176, 194	164, 178	260, 266	248, 251	251, 254
		9IIIA		222, 228	232, 238	202, 208	232, 248	178, 194, 202	176, 194	164, 178	260, 266	248, 251	251, 254
	III-B	9IIIB	30-40	222, 228	232, 238	202, 208	232, 248	178, 194, 202	176, 194	164, 178	260, 266	248, 251	251, 254
		9IIIB		222, 228	232, 238	202, 208	232, 248	178, 194, 202	176, 194	164, 178	260, 266	248, 251	251, 254
	III-C	9IIIC	50-60	222, 228	232, 238	202, 208	232, 248	178, 194, 202	176, 194	164, 178	260, 266	248, 251	251, 254
		9IIIC		222, 228	232, 238	202, 208	232, 248	178, 194, 202	176, 194	164, 178	260, 266	248, 251	251, 254
	III-D	9IIID	50-60	222, 228	232, 238	202, 208	232, 248	178, 194, 202	176, 194	164, 178	260, 266	248, 251	251, 254
		9IIID		222, 228	232, 238	202, 208	232, 248	178, 194, 202	176, 194	164, 178	260, 266	248, 251	251, 254
Quadrant III	III-E	9IIIE	20-30	222, 228	232, 238	202, 208	232, 248	178, 194, 202	176, 194	164, 178	260, 266	248, 251	251, 254
		9IIIE		222, 228	fail	202, 208	232, 248	178, 194, 202	176, 194	164, 178	260, 266	248, 251	251, 254
		9IIIF		222, 228	fail	202, 208	232, 248	178, 194, 202	176, 194	164, 178	260, 266	248, 251	251, 254
	III-F	9IIIF	20-30	222, 228	232, 238	202, 208	232, 248	178, 194, 202	176, 194	164, 178	260, 266	248, 251	251, 254
		9IIIF		222, 228	232, 238	202, 208	232, 248	178, 194, 202	176, 194	164, 178	260, 266	248, 251	251, 254
		9IIIG		222, 228	232, 238	202, 208	232, 248	fail	176, 194	164, 178	260, 266	248, 251	251, 254
	III-G	9IIIG	20-30	222, 228	232, 238	202, 208	232, 248	178, 194, 202	176, 194	164, 178	260, 266	248, 251	251, 254
		9IIIG		222, 228	232, 238	202, 208	232, 248	178, 194, 202	176, 194	164, 178	260, 266	248, 251	251, 254
		9IVA		222, 228	232, 238	202, 208	232, 248	178, 194, 202	176, 194	164, 178	260, 266	248, 251	251, 254
	IV-A	9IVA	surface to 10	222, 228	232, 238	202, 208	232, 248	178, 194, 202	176, 194	164, 178	260, 266	248, 251	251, 254
		9IVA		222, 228	232, 238	202, 208	232, 248	178, 194, 202	176, 194	164, 178	260, 266	248, 251	251, 254
		9IVB		222, 228	232, 238	202, 208	232, 248	178, 194, 202	176, 194	164, 178	260, 266	248, 251	251, 254
Quadrant IV	IV-B	9IVB	20-30	222, 228	232, 238	202, 208	232, 248	178, 194, 202	176, 194	164, 178	260, 266	248, 251	251, 254
		9IVB		222, 228	232, 238	202, 208	232, 248	178, 194, 202	176, 194	164, 178	260, 266	248, 251	251, 254
		9IVC		222, 228	232, 238	202, 208	232, 248	178, 194, 202	176, 194	164, 178	260, 266	248, 251	251, 254
	IV-C	9IVC	surface to 10	222, 228	232, 238	202, 208	232, 248	178, 194, 202	176, 194	164, 178	260, 266	248, 251	251, 254
		9IVC		222, 228	232, 238	202, 208	232, 248	178, 194, 202	176, 194	164, 178	fail	248, 251	251, 254

Table S3: Colony 12

GPS: N09º 09' 27.7" = N09.1577°
W79º 44' 51.1" = W79.7475°

Mound diameter: 15m x 11m

Nest Quadrant	Garden ID	Sample Name	Excavation Depth (cm)	A128 (AC)$_n$	A435 (GT)$_n$	A1132 (TCA)$_n$	B12 (GA)$_n$	B150 (CT)$_n$	B312 (GA)$_n$	B430 (TC)$_n$	C117 (CAG)$_n$(CAC)$_n$	C1133 (TGC)$_n$-A-(GGA)$_n$	D115 (TCA)$_n$-TCG-(TCA)$_n$
		12IA		222, 228	232, 238	202, 208	232, 252	194, 202	176, 194	164, 178	264, 267	248, 251	251, 254
	I-A	12IA	40-50	222, 228	232, 238	202, 208	232, 252	194, 202	176, 194	164, 178	264, 267	248, 251	251, 254
		12IA		222, 228	232, 238	202, 208	232, 252	194, 202	176, 194	164, 178	264, 267	248, 251	251, 254
		12IB		222, 228	232, 238	202, 208	232, 252	194, 202	176, 194	164, 178	264, 267	248, 251	251, 254
	I-B	12IB	40-50	222, 228	232, 238	202, 208	232, 252	194, 202	176, 194	164, 178	264, 267	248, 251	251, 254
		12IB		222, 228	232, 238	202, 208	232, 252	194, 202	176, 194	164, 178	264, 267	248, 251	251, 254
		12IC		222, 228	232, 238	202, 208	232, 252	194, 202	176, 194	164, 178	264	248, 251	251, 254
	I-C	12IC	50-60	222, 228	232, 238	202, 208	232, 252	194, 202	176, 194	164, 178	264	248, 251	251, 254
Quadrant I		12IC		222, 228	232, 238	202, 208	232, 252	194, 202	176, 194	164, 178	264, 267	248, 251	251, 254
		12ID		222, 228	232, 238	202, 208	232, 252	194, 202	176, 194	164, 178	264, 267	248, 251	251, 254
	I-D	12ID	30-40	222, 228	232, 238	202, 208	232, 252	194, 202	176, 194	164, 178	264, 267	248, 251	251, 254
		12ID		222, 228	232, 238	202, 208	232, 252	194, 202	176, 194	164, 178	264, 267	248, 251	251, 254
		12IE		222, 228	232, 238	202, 208	232, 252	194, 202	176, 194	164, 178	264, 267	248, 251	251, 254
	I-E	12IE	30-40	222, 228	232, 238	202, 208	232, 252	194, 202	176, 194	164, 178	264, 267	248, 251	251, 254
		12IE		222, 228	232, 238	202, 208	232, 252	194, 202	176, 194	164, 178	264, 267	248, 251	251, 254
		12IF		222, 228	232, 238	202, 208	232, 252	194, 202	176, 194	164, 178	264, 267	248, 251	251, 254
	I-F	12IF	30-40	222, 228	232, 238	202, 208	232, 252	194, 202	176, 194	164, 178	264, 267	248, 251	251, 254
		12IF		222, 228	232, 238	202, 208	232, 252	194, 202	176, 194	164, 178	264, 267	248, 251	251, 254
		12IIA		222, 228	232, 238	202, 208	232, 252	194, 202	176, 194	164, 178	264, 267	248, 251	251, 254
	II-A	12IIA	30-40	222, 228	232, 238	202, 208	232, 252	194, 202	176, 194	164, 178	264, 267	248, 251	251, 254
		12IIA		222, 228	232, 238	202, 208	232, 252	194, 202	176, 194	164, 178	264, 267	248, 251	251, 254
		12IIB		222, 228	232, 238	202, 208	232, 252	194, 202	176, 194	164, 178	264, 267	248, 251	251, 254
	II-B	12IIB	50-60	222, 228	232, 238	202, 208	232, 252	194, 202	176, 194	164, 178	264, 267	248, 251	251, 254
		12IIB		222, 228	232, 238	202, 208	232, 252	194, 202	176, 194	164, 178	264, 267	248, 251	251, 254
		12IIC		222, 228	232, 238	202, 208	232, 252	194, 202	176, 194	164, 178	264, 267	248, 251	251, 254
	II-C	12IIC	30-40	222, 228	232, 238	202, 208	232, 252	194, 202	176, 194	164, 178	264, 267	248, 251	251, 254
		12IIC		222, 228	232, 238	202, 208	232, 252	194, 202	176, 194	164, 178	264, 267	248, 251	251, 254
		12IID		222, 228	232, 238	202, 208	232, 252	194, 202	176, 194	164, 178	264, 267	248, 251	251, 254
	II-D	12IID	40-50	222, 228	232, 238	202, 208	232, 252	194, 202	176, 194	164, 178	264, 267	248, 251	251, 254
		12IID		222, 228	232, 238	202, 208	232, 252	194, 202	176, 194	164, 178	264, 267	248, 251	251, 254
		12IIE		222, 228	232, 238	202, 208	232, 252	194, 202	176, 194	164, 178	264, 267	248, 251	251, 254
Quadrant II	II-E	12IIE	50-60	222, 228	232, 238	202, 208	232, 252	194, 202	176, 194	164, 178	264, 267	248, 251	251, 254
		12IIE		222, 228	232, 238	202, 208	232, 252	194, 202	176, 194	164, 178	264, 267	248, 251	251, 254
		12IIF		222, 228	232, 238	202, 208	232, 252	194, 202	176, 194	164, 178	264, 267	248, 251	251, 254
	II-F	12IIF	30-40	222, 228	232, 238	202, 208	232, 252	194, 202	176, 194	164, 178	264, 267	248, 251	251, 254
		12IIF		222, 228	232, 238	202, 208	232, 252	194, 202	176, 194	164, 178	264, 267	248, 251	251, 254
		12IIG		222, 228	232, 238	202, 208	232, 252	194, 202	176, 194	164, 178	264, 267	248, 251	251, 254
	II-G	12IIG	20-30	222, 228	232, 238	202, 208	232, 252	194, 202	176, 194	164, 178	264, 267	248, 251	251, 254
		12IIG		222, 228	232, 238	202, 208	232, 252	194, 202	176, 194	164, 178	264, 267	248, 251	251, 254
		12IIH		222, 228	232, 238	202, 208	232, 252	194, 202	176, 194	164, 178	264, 267	248, 251	251, 254
	II-H	12IIH	30-40	222, 228	232, 238	202, 208	232, 252	194, 202	176, 194	164, 178	264, 267	248, 251	251, 254
		12IIH		222, 228	232, 238	202, 208	232, 252	194, 202	176, 194	164, 178	264, 267	248, 251	251, 254
		12II_I		222, 228	232, 238	202, 208	232, 252	194, 202	176, 194	164, 178	264, 267	248, 251	251, 254
	II-I	12II_I	20-30	222, 228	232, 238	202, 208	232, 252	194, 202	176, 194	164, 178	264, 267	248, 251	251, 254
		12II_I		222, 228	232, 238	202, 208	232, 252	194, 202	176, 194	164, 178	264, 267	248, 251	251, 254
		12IIIA		222, 228	232, 238	202, 208	232, 252	194, 202	176, 194	164, 178	264, 267	248, 251	251, 254
	III-A	12IIIA	20-30	222, 228	232, 238	202, 208	232, 252	194, 202	176, 194	164, 178	264, 267	248, 251	251, 254
		12IIIA		222, 228	232, 238	202, 208	232, 252	194, 202	176, 194	164, 178	264, 267	248, 251	251, 254
		12IIIB		222, 228	232, 238	202, 208	232, 252	194, 202	176, 194	164, 178	264, 267	248, 251	251, 254
	III-B	12IIIB	30-40	222, 228	232, 238	202, 208	232, 252	194, 202	176, 194	164, 178	264, 267	248, 251	251, 254
		12IIIB		222, 228	232, 238	202, 208	232, 252	194, 202	176, 194	164, 178	264, 267	248, 251	251, 254
		12IIIC		222, 228	232, 238	202, 208	232, 252	194, 202	176, 194	164, 178	264, 267	248, 251	251, 254
	III-C	12IIIC	40-50	222, 228	232, 238	202, 208	232, 252	194, 202	176, 194	164, 178	264, 267	248, 251	251, 254
		12IIIC		222, 228	232, 238	202, 208	232, 252	194, 202	176, 194	164, 178	264, 267	248, 251	251, 254
		12IIID		222, 228	232, 238	202, 208	232, 252	194, 202	176, 194	164, 178	264, 267	248, 251	251, 254
	III-D	12IIID	30-40	222, 228	232, 238	202, 208	232, 252	194, 202	176, 194	164, 178	264, 267	248, 251	251, 254
		12IIID		222, 228	232, 238	202, 208	232, 252	194, 202	176, 194	164, 178	264, 267	248, 251	251, 254
		12IIIE		222, 228	232, 238	202, 208	232, 252	194, 202	176, 194	164, 178	264, 267	248, 251	251, 254
Quadrant III	III-E	12IIIE	20-30	222, 228	232, 238	202, 208	232, 252	194, 202	176, 194	164, 178	264, 267	248, 251	251, 254
		12IIIE		222, 228	232, 238	202, 208	232, 252	194, 202	176, 194	164, 178	264, 267	248, 251	251, 254
		12IIIF		222, 228	232, 238	202, 208	232, 252	194, 202	176, 194	164, 178	264, 267	248, 251	251, 254
	III-F	12IIIF	20-30	222, 228	232, 238	202, 208	232, 252	194, 202	176, 194	164, 178	264, 267	248, 251	251, 254
		12IIIF		222, 228	232, 238	202, 208	232, 252	194, 202	176, 194	164, 178	264, 267	248, 251	251, 254
		12IIIG		222, 228	232, 238	202, 208	232, 252	194, 202	176, 194	164, 178	264, 267	248, 251	251, 254
	III-G	12IIIG	20-30	222, 228	232, 238	202, 208	232, 252	194, 202	176, 194	164, 178	264, 267	248, 251	251, 254
		12IIIG		222, 228	232, 238	202, 208	232, 252	194, 202	176, 194	164, 178	264, 267	248, 251	251, 254
		12IIIH		222, 228	232, 238	202, 208	232, 252	194, 202	176, 194	164, 178	264, 267	248, 251	251, 254
	III-H	12IIIH	30-40	222, 228	232, 238	202, 208	232, 252	194, 202	176, 194	164, 178	264, 267	248, 251	251, 254
		12IIIH		222, 228	232, 238	202, 208	232, 252	194, 202	176, 194	164, 178	264, 267	248, 251	251, 254
		12III_I		222, 228	232, 238	202, 208	232, 252	194, 202	176, 194	164, 178	264, 267	248, 251	251, 254
	III-I	12III_I	40-50	222, 228	232, 238	202, 208	232, 252	194, 202	176, 194	164, 178	fail	248, 251	251, 254
		12III_I		222, 228	232, 238	202, 208	232, 252	194, 202	176, 194	164, 178	264	248, 251	251, 254

Table S3: Colony 13

GPS: N09º 09' 30.3" = N09.1584°
W79º 44' 49.5" = W79.7471°

Mound diameter: 20m x 40m

Nest Quadrant	Garden ID	Sample Name	Excavation Depth (cm)	A128 $(AC)_n$	A435 $(GT)_n$	A1132 $(TCA)_n$	B12 $(GA)_n$	B150 $(CT)_n$	B312 $(GA)_n$	B430 $(TC)_n$	C117 $(CAG)_n(CAC)_n$	C1133 $(TGC)_n$-A-$(GGA)_n$	D115 $(TCA)_n$-TCG-$(TCA)_n$
		13IA		222, 226	234	202, 214	232	178, 186	168, 176	168, 174	264	233, 245	252, 267
	I-A	13IA	50-60	222, 226	234	202, 214	232	178, 186	168, 176	168, 174	264	233, 245	252, 267
		13IA		222, 226	234	202, 214	232	178, 186	168, 176	168, 174	264	233, 245	252, 267
	I-B	13IB	50-60	222, 226	234	202, 214	232	178, 186	168, 176	168, 174	264	233, 245	252, 267
		13IB		222, 226	234	202, 214	232	178, 186	168, 176	168, 174	264	233, 245	252, 267
		13IC		222, 226	234	202, 214	232	178, 186	168, 176	168, 174	264	233, 245	252, 267
	I-C	13IC	20-30	222, 226	234	202, 214	232	178, 186	168, 176	168, 174	264	233, 245	252, 267
		13IC		fail	fail	202, 214	232	178, 186	168, 176	168, 174	264	233, 245	252, 267
		13ID		222, 226	234	202, 214	232	178, 186	168, 176	168, 174	264	233, 245	252, 267
Quadrant I	I-D	13ID	50-60	222, 226	234	202, 214	232	178, 186	168, 176	168, 174	264	233, 245	252, 267
		13ID		222, 226	234	202, 214	232	178, 186	168, 176	168, 174	264	233, 245	252, 267
		13IE		222, 226	234	202, 214	232	178, 186	168, 176	168, 174	264	233, 245	252, 267
	I-E	13IE	40-50	222, 226	234	202, 214	232	178, 186	168, 176	168, 174	264	233, 245	252, 267
		13IE		222, 226	234	202, 214	232	178, 186	168, 176	168, 174	264	233, 245	252, 267
		13IF		222, 226	fail	202, 214	232	178, 186	168, 176	168, 174	264	233, 245	252, 267
	I-F	13IF	50-60	222, 226	234	202, 214	232	178, 186	168, 176	168, 174	264	233, 245	252, 267
		13IF		222, 226	234	202, 214	232	178, 186	168, 176	168, 174	264	233, 245	252, 267
		13IG		226	234	202, 214	232	178, 186	168, 176	168, 174	264	233, 245	252, 267
	I-G	13IG	30-40	222, 226	234	202, 214	232	178, 186	168, 176	168, 174	264	233, 245	252, 267
		13IG		222, 226	234	202, 214	232	178, 186	168, 176	168, 174	264	233, 245	252, 267
		13IIA		222, 226	234	202, 214	232	178, 186	168, 176	168, 174	264	233, 245	252, 267
	II-A	13IIA	50-60	222, 226	234	202, 214	232	178, 186	168, 176	168, 174	264	233, 245	252, 267
		13IIA		222, 226	234	202, 214	232	178, 186	168, 176	168, 174	264	233, 245	252, 267
		13IIB		222, 226	234	202, 214	232	178, 186	168, 176	168, 174	264	233, 245	252, 267
	II-B	13IIB	40-50	222, 226	234	202, 214	232	178, 186	168, 176	168, 174	264	233, 245	252, 267
		13IIB		222, 226	234	202, 214	232	178, 186	168, 176	168, 174	264	233, 245	252, 267
		13IIC		222, 226	234	202, 214	232	178, 186	168, 176	168, 174	264	233, 245	252, 267
	II-C	13IIC	30-40	222, 226	234	202, 214	232	178, 186	168, 176	168, 174	264	233, 245	252, 267
		13IIC		222, 226	234	202, 214	232	178, 186	168, 176	168, 174	264	233, 245	252, 267
		13IID		222, 226	234	202, 214	232	178, 186	168, 176	168, 174	264	233, 245	252, 267
	II-D	13IID	30-40	222, 226	234	202, 214	232	178, 186	168, 176	168, 174	264	233, 245	252, 267
		13IID		222, 226	234	202, 214	232	178, 186	168, 176	168, 174	264	233, 245	252, 267
		13IIE		222, 226	234	202, 214	232	178, 186	168, 176	168, 174	264	233, 245	252, 267
Quadrant II	II-E	13IIE	40-50	222, 226	234	202, 214	232	178, 186	168, 176	168, 174	264	233, 245	252, 267
		13IIE		222, 226	234	202, 214	232	178, 186	168, 176	168, 174	264	233, 245	252, 267
		13IIF		222, 226	234	202, 214	232	178, 186	168, 176	168, 174	264	233, 245	252, 267
	II-F	13IIF	40-50	222, 226	234	202, 214	232	178, 186	168, 176	168, 174	264	233, 245	252, 267
		13IIF		222, 226	234	202, 214	232	178, 186	168, 176	168, 174	264	233, 245	252, 267
		13IIG		222, 226	234	202, 214	232	178, 186	168, 176	168, 174	264	233, 245	252, 267
	II-G	13IIG	40-50	222, 226	234	202, 214	232	178, 186	168, 176	168, 174	264	233, 245	252, 267
		13IIG		222, 226	234	202, 214	232	178, 186	168, 176	168, 174	264	233, 245	252, 267
		13IIH		222, 226	234	202, 214	232	178, 186	168, 176	168, 174	264	233, 245	252, 267
	II-H	13IIH	40-50	222, 226	234	202, 214	232	178, 186	168, 176	168, 174	264	233, 245	252, 267
		13IIH		222, 226	234	202, 214	232	178, 186	168, 176	168, 174	264	233, 245	252, 267
		13II_I		222, 226	234	202, 214	232	178, 186	168, 176	168, 174	264	233, 245	252, 267
	II-I	13II_I	50-60	222, 226	234	202, 214	232	178, 186	168, 176	168, 174	264	233, 245	252, 267
		13II_I		222, 226	234	202, 214	232	178, 186	168, 176	168, 174	264	233, 245	252, 267
		13IIIA		222, 226	234	202, 214	232	178, 186	168, 176	168, 174	264	233, 245	252, 267
	III-A	13IIIA	40-50	222, 226	234	202, 214	232	178, 186	168, 176	168, 174	264	233, 245	252, 267
		13IIIA		222, 226	234	202, 214	232	178, 186	168, 176	168, 174	264	233, 245	252, 267
		13IIIB		222, 226	234	202, 214	232	178, 186	168, 176	168, 174	264	233, 245	252, 267
	III-B	13IIIB	30-40	222, 226	234	202, 214	232	178, 186	168, 176	168, 174	264	233, 245	252, 267
		13IIIB		222, 226	234	202, 214	232	178, 186	168, 176	168, 174	264	233, 245	252, 267
		13IIIC		222, 226	234	202, 214	232	178, 186	168, 176	168, 174	264	233, 245	252, 267
	III-C	13IIIC	20-30	222, 226	234	202, 214	232	178, 186	168, 176	168, 174	264	233, 245	252, 267
		13IIIC		222, 226	234	202, 214	232	178, 186	168, 176	168, 174	264	233, 245	252, 267
		13IIID		222, 226	234	202, 214	232	178	168, 176	168, 174	264	233, 245	252, 267
	III-D	13IIID	40-50	222, 226	234	202, 214	232	178, 186	168, 176	168, 174	264	233, 245	252, 267
		13IIID		222, 226	234	202, 214	232	178, 186	168, 176	168, 174	264	233, 245	252, 267
		13IIIE		222, 226	234	202, 214	232	178, 186	168, 176	168, 174	264	233, 245	252, 267
Quadrant III	III-E	13IIIE	40-50	222, 226	234	202, 214	232	178, 186	168, 176	168, 174	264	233, 245	252, 267
		13IIIE		222, 226	234	202, 214	232	178, 186	168, 176	168, 174	264	233, 245	252, 267
		13IIIF		222, 226	234	202, 214	232	178, 186	168, 176	168, 174	264	233, 245	252, 267
	III-F	13IIIF	40-50	222, 226	234	202, 214	232	178, 186	168, 176	168, 174	264	233, 245	252, 267
		13IIIF		222, 226	234	202, 214	232	178, 186	168, 176	168, 174	264	233, 245	252, 267
		13IIIG		222, 226	234	202, 214	232	178, 186	168, 176	168, 174	264	233, 245	252, 267
	III-G	13IIIG	30-40	222, 226	234	202, 214	232	178, 186	168, 176	168, 174	264	233, 245	252, 267
		13IIIG		222, 226	234	202, 214	232	178, 186	168, 176	168, 174	264	233, 245	252, 267
		13IIIH		222, 226	234	202, 214	232	178, 186	168, 176	168, 174	264	233, 245	252, 267
	III-H	13IIIH	40-50	222, 226	234	202, 214	232	178, 186	168, 176	168, 174	264	233, 245	252, 267
		13IIIH		222, 226	234	202, 214	232	178, 186	fail	168, 174	264	233, 245	252, 267
		13III_I		222, 226	234	202, 214	232	178, 186	fail	168, 174	264	233, 245	252, 267
	III-I	13III_I	40-50	222, 226	234	202, 214	232	178, 186	168, 176	168, 174	264	233, 245	252, 267
		13III_I		222, 226	234	202, 214	232	178, 186	168, 176	168, 174	264	233, 245	252, 267
