# Supplementary material for: Monoculture of Leafcutter Ant Gardens
Source: PLoS One. 2010 Sep 10;5(9):e12668. doi: 10.1371/journal.pone.0012668 (PMC2937030; doi:10.1371/journal.pone.0012668)
Supplement: Table S4 — Attamyces garden genotyping 2004&2008 for Atta cephalotes. (0.06 MB PDF) [file pone.0012668.s005.pdf]

Table S4. Repeat microsatellite genotyping of *Attamyces* cultivars from *Atta cephalotes colonies* surveyed in 2003 and 2008.

Garden samples were collected through excavation from the same four nests of *Atta cephalotes* (see Materials and Methods and caption for Table S3)

Sample IDs from Dec2003 in the leftmost column (e.g., I-A or IV-D) refer to the the garden IDs shown in the second column in Table S3.

Samples were genotyped at 15 microsatellite loci through multiplex analysis, as described in Ishak et al. (in preparation; see also Material and Methods).

Markers were scored blind without knowledge of identity of a particular sample.

1 = marker present. 0 = marker absent. ? = unclear whether allele is present or absent.

The label "fail" indicates that the analysis failed for the locus and the analysis was not repeated.

Within each nest, no marker differed between 2003 and 2008.

Nest & Sample ID	Collection Date	C101				C606		B12				D115			B150				C117			A1030	
		108	111	120	123	138	147	193	198	202	214	235	239	250	79	86	95	103	171	174	177	196	198
**Colony 2**																							
**2 I-A Dec2003**	**December 2003**	0	1	0	?	1	0	0	1	0	0	fail	fail	fail	1	1	0	0	0	1	0	1	0
**2 IV-D Dec2003**	**December 2003**	0	1	0	1	1	0	0	1	0	0	1	0	1	1	1	0	0	0	1	0	1	0
**2-1 Jun2008**	**June 2008**	0	1	0	1	1	0	0	1	0	0	1	0	1	1	1	0	0	0	1	0	1	0
**2-2 Jun2008**	**June 2008**	0	1	0	?	fail	fail	0	1	0	0	1	0	1	1	1	0	0	0	1	0	1	0
**Colony 9**																							
**9 I-C Dec2003**	**December 2003**	1	0	?	?	1	1	1	0	1	1	1	1	0	0	0	1	0	1	1	1	fail	fail
**9 I-F Dec2003**	**December 2003**	1	0	1	1	1	1	1	0	1	1	1	1	0	0	0	1	0	1	1	1	fail	fail
**9-1 Jun2008**	**June 2008**	1	0	1	1	1	1	1	?	1	1	1	1	0	0	0	1	0	1	1	1	0	1
**9-2 Jun2008**	**June 2008**	1	0	1	1	?	1	fail	fail	fail	fail	1	1	0	0	0	1	0	1	1	1	0	1
**Colony 12**																							
**12 I-A Dec2003**	**December 2003**	1	0	1	1	1	1	fail	fail	fail	fail	1	?	0	0	0	1	1	0	1	0	0	1
**12 I-C Dec2003**	**December 2003**	1	0	1	1	1	1	fail	fail	fail	fail	1	1	0	0	0	1	1	0	1	0	0	1
**12-1 Jun2008**	**June 2008**	1	0	1	1	1	1	fail	fail	fail	fail	1	1	0	0	0	1	1	0	1	0	0	1
**12-2 Jun2008**	**June 2008**	1	0	1	1	1	1	fail	fail	fail	fail	1	1	0	0	0	1	1	0	1	0	0	1
**Colony 13**																							
**13 I-D Dec2003**	**December 2003**	0	1	0	1	1	0	fail	fail	fail	fail	1	0	1	1	1	0	0	0	1	0	1	0
**13 I-G Dec2003**	**December 2003**	0	1	0	1	1	0	fail	fail	fail	fail	1	0	1	1	1	0	0	0	1	0	1	0
**13-1 Jun2008**	**June 2008**	0	1	0	1	1	0	fail	fail	fail	fail	1	0	1	1	1	0	0	0	1	0	1	0
**13-2 Jun2008**	**June 2008**	0	1	0	1	1	0	fail	fail	fail	fail	1	0	1	1	1	0	0	0	1	0	1	0

A435				A1132			B319		A1151				A128					C126		B430				C1133				
**233**	**235**	**236**	**239**	**105**	**111**	**118**	**195**	**201**	**92**	**98**	**104**	**106**	**201**	**204**	**208**	**210**	**212**	**244**	**246**	**146**	**150**	**156**	**160**	**218**	**228**	**230**	**233**	**236**
1	1	0	0	1	0	1	1	0	1	0	1	0	1	1	1	0	0	fail	fail	0	1	1	0	fail	fail	fail	fail	fail
1	1	0	0	1	0	1	1	0	1	0	1	0	1	1	1	0	0	1	1	0	1	1	0	1	1	1	0	0
1	1	0	0	1	0	1	1	0	1	0	1	0	1	1	1	0	0	1	1	0	1	1	0	1	1	1	0	0
1	1	0	0	1	0	1	fail	fail	1	0	1	0	1	1	1	0	0	1	1	0	1	1	0	1	1	1	0	0
1	0	1	1	1	1	1	0	1	0	1	0	1	0	1	0	0	1	fail	fail	1	0	0	1	0	0	0	1	1
1	0	1	1	1	1	1	0	1	0	1	0	1	0	1	0	0	1	1	1	1	0	0	1	0	0	0	1	1
1	0	1	1	1	1	1	0	1	0	1	0	1	0	1	0	0	1	1	1	1	0	0	1	0	0	0	1	1
1	0	1	1	1	1	1	0	1	0	1	0	1	0	1	0	0	1	1	1	1	0	0	1	0	0	0	1	1
1	0	1	1	1	1	0	0	1	0	1	0	1	0	1	0	0	1	fail	fail	1	0	0	1	0	0	0	1	1
1	0	1	1	1	1	0	0	1	0	1	0	1	0	1	0	0	1	fail	fail	fail	fail	fail	fail	0	0	0	1	1
1	0	1	1	1	1	0	0	1	0	1	0	1	0	1	0	0	1	1	1	1	0	0	1	0	0	0	1	1
1	0	1	1	1	1	0	0	1	0	1	0	1	0	1	0	0	1	1	1	1	0	0	1	0	0	0	1	1
1	1	0	0	1	0	1	fail	fail	1	0	1	0	0	1	0	1	0	1	1	0	1	1	0	1	0	1	0	0
1	1	0	0	1	0	1	fail	fail	1	0	1	0	0	1	0	1	0	1	1	0	1	1	0	1	0	1	0	0
1	1	0	0	1	0	1	1	1	1	0	1	0	0	1	0	1	0	1	1	0	1	1	0	1	0	1	0	0
1	1	0	0	1	0	1	1	1	1	0	1	0	0	1	0	1	0	1	1	0	1	1	0	1	0	1	0	0
