# Supplementary material for: Monoculture of Leafcutter Ant Gardens
Source: PLoS One. 2010 Sep 10;5(9):e12668. doi: 10.1371/journal.pone.0012668 (PMC2937030; doi:10.1371/journal.pone.0012668)
Supplement: Table S5 — Attamyces colony-forming units per pellet for Atta texana. (0.06 MB PDF) [file pone.0012668.s006.pdf]

Table S5. Number of colony-forming units (CFUs) of *Attamyces* in infrabuccal pellets carried by *Atta texana* females.
Pellets were sterilely expelled from dispersing female *A. texana*, then macerated and vortexed in 1 ml buffer.
The entire suspension was plated on potato dextrose agar.
CFUs of *Attamyces* were counted after three weeks growth at room temperature.

Pellet #	Nest ID, Collection Location	CFU/ml
1	Nest 1 (UGM050509-01, Brackenridge Field Lab, Austin, TX)	800
2	Nest 1 (UGM050509-01, Brackenridge Field Lab, Austin, TX)	673
3	Nest 1 (UGM050509-01, Brackenridge Field Lab, Austin, TX)	860
4	Nest 1 (UGM050509-01, Brackenridge Field Lab, Austin, TX)	613
5	Nest 1 (UGM050509-01, Brackenridge Field Lab, Austin, TX)	600
6	Nest 1 (UGM050509-01, Brackenridge Field Lab, Austin, TX)	900
7	Nest 1 (UGM050509-01, Brackenridge Field Lab, Austin, TX)	1166
8	Nest 1 (UGM050509-01, Brackenridge Field Lab, Austin, TX)	686
9	Nest 1 (UGM050509-01, Brackenridge Field Lab, Austin, TX)	1500
10	Nest 1 (UGM050509-01, Brackenridge Field Lab, Austin, TX)	893
11	Nest 1 (UGM050509-01, Brackenridge Field Lab, Austin, TX)	155
12	Nest 1 (UGM050509-01, Brackenridge Field Lab, Austin, TX)	195
13	Nest 1 (UGM050509-01, Brackenridge Field Lab, Austin, TX)	280
14	Nest 1 (UGM050509-01, Brackenridge Field Lab, Austin, TX)	280
15	Nest 1 (UGM050509-01, Brackenridge Field Lab, Austin, TX)	220
16	Nest 1 (UGM050509-01, Brackenridge Field Lab, Austin, TX)	190
17	Nest 1 (UGM050509-01, Brackenridge Field Lab, Austin, TX)	535
18	Nest 1 (UGM050509-01, Brackenridge Field Lab, Austin, TX)	720
19	Nest 1 (UGM050509-01, Brackenridge Field Lab, Austin, TX)	690
20	Nest 1 (UGM050509-01, Brackenridge Field Lab, Austin, TX)	60
	**AVERAGE =**	**600.8**
	**STANDARD DEVIATION =**	**371.7**
	**RANGE =**	**60-1500**
21	Nest A (UGM060121-01, Hornsby Bend, Austin, TX)	225
22	Nest A (UGM060121-01, Hornsby Bend, Austin, TX)	350
23	Nest A (UGM060121-01, Hornsby Bend, Austin, TX)	735
24	Nest A (UGM060121-01, Hornsby Bend, Austin, TX)	940
25	Nest A (UGM060121-01, Hornsby Bend, Austin, TX)	200
26	Nest A (UGM060121-01, Hornsby Bend, Austin, TX)	315
27	Nest A (UGM060121-01, Hornsby Bend, Austin, TX)	248
28	Nest A (UGM060121-01, Hornsby Bend, Austin, TX)	188
	**AVERAGE =**	**400.1**
	**STANDARD DEVIATION =**	**280.9**
	**RANGE =**	**188-940**
	**OVERALL UNWEIGHTED AVERAGE =**	**543.464**
	**OVERALL STANDARD DEVIATION =**	**355.21**
	**OVERALL RANGE =**	**60-1500**
